# Aerobic physical activity, cardiorespiratory fitness, and non-communicable diseases risk in older adults: a systematic review

**DOI:** 10.1186/s12877-026-07541-4

**Published:** 2026-04-30

**Authors:** Andrea M. Madden, Larske M. Soepnel, Chad Africa, Janice L. Rhoda, Zeena Morar, Estelle V. Lambert, Sam Gutterman, Francois Millard, Howard Bolnick, Jessica C. Davies, Lara R. Dugas

**Affiliations:** 1 Division of Epidemiology and Biostatistics, School of Public Health, Faculty of Health Sciences, University of Cape Town, Cape Town, South Africa; 2 Department of Global Public Health and Bioethics, Julius Center for Health Sciences and Primary Care, University Medical Center Utrecht, Utrecht, the Netherlands; 3 Research Centre for Health through Physical Activity, Lifestyle and Sport (HPALS), Faculty of Health Sciences, University of Cape Town, Cape Town, South Africa; 4 School of Health & Medical Sciences, Faculty of Health, Engineering & Sciences, University of Southern Queensland, Toowoomba, Australia; 5 Human Kinetics and Ergonomics, Rhodes University, Makhanda, South Africa; 6 Consulting Actuary, Chicago, IL USA; 7 Vitality Group, Chicago, IL USA; 8 Public Health Sciences, Parkinson School of Health Sciences and Public Health, Loyola University Chicago, Maywood, IL USA

**Keywords:** Aerobic physical activity, Cardiorespiratory fitness, Older adults, Non-communicable diseases, Cardiovascular disease, Mortality, Frailty/falls

## Abstract

**Background:**

Non-communicable diseases (NCDs) account for an estimated 80% of the healthcare burden among older adults globally. Aerobic physical activity (PA) contributes to functional mobility, increased bone and muscle strength, prevention of illness and quality of life; however, PA is not yet a fully realised aspect of geriatric care.

**Objective:**

To review recent evidence for the impact of aerobic PA and cardiorespiratory fitness (CRF) on mortality and NCD outcomes in adults aged ≥ 65 years.

**Methods:**

Following PRISMA guidelines, we performed a systematic search of the Scopus, Cochrane Library, PubMed and Web of Science databases, published between 2016 and February 2024.

**Results:**

The 94 included studies (reported in 104 individual manuscripts) included 46 cross-sectional, 24 longitudinal, 7 cross-sectional/longitudinal, 1 intervention/cross-sectional, and 16 interventions studies. PA and CRF were consistently associated with reduced all-cause mortality in longitudinal studies. Most included studies reported inverse associations between measures of PA and cardiovascular disease (CVD, *n* = 14 of 16 studies) risk and measures of dysglycaemia (*n* = 12 of 16 studies). Obesity/adiposity, blood pressure, lipids, and metabolic syndrome (MetS) were not consistently associated with aerobic PA. While aerobic PA interventions generally improved depression outcomes, longitudinal studies showed inconclusive results. Observational evidence suggests an association between PA and frailty, but less consistently with falls.

**Conclusion:**

This review suggests a beneficial association between aerobic PA and all-cause mortality, CVD, dysglycaemia, and frailty, with inconsistent evidence for associations with obesity, dyslipidaemia, hypertension, MetS, depression, and falls. Given the inconsistent findings for key metabolic outcomes, future research is needed to address outstanding and understudied questions around risk reversibility and time to benefit for PA interventions, as well as identifying target groups who benefit more from PA and tailoring interventions to these groups. Further longitudinal studies are also needed to determine the optimal frequency, duration, and intensity of aerobic PA for mitigating NCD risk in older adults.

**Supplementary Information:**

The online version contains supplementary material available at 10.1186/s12877-026-07541-4.

## Highlights

We found inconsistent evidence for the association between aerobic PA and obesity/adiposity, blood pressure, lipid profiles, and MetS.

All included studies reported inverse associations between all-cause mortality, and at least some measures of PA and CRF.

Generally, aerobic PA interventions improved depression risk and depression scores.

At-home-based exercise interventions may be less effective in reducing frailty.

**Supplementary Information:**

The online version contains supplementary material available at 10.1186/s12877-026-07541-4.

## Introduction

Over the last 50 years, the number of adults aged 65 years and older (older adults) has tripled globally, and this number is projected to further double by 2050, from 761 million to an estimated 1.6 billion older adults [1]. Since ageing increases the susceptibility to chronic NCDs, this demographic shift is interwoven with the exponential global rise in NCDs [1, 2]. Data from the Global Burden of Disease Study 2021 indicates that an estimated 64.5% of deaths worldwide are attributable to NCDs [3], while in older adults, NCDs account for about 80% of the healthcare burden [4]. In addition, while more than half of older adults are estimated to suffer from a single chronic illness, a third are compromised by two or more chronic diseases [5]. Low- and middle-income countries are no longer an exception to the rise in global NCDs, including among ageing populations [4], resulting in additional strain on limited healthcare resources. As such, NCDs pose a major threat to the physical and mental health, social participation, quality of life, life expectancy and financial security among older adults [6].

Numerous studies support the benefits of PA on NCDs, such as CVD and type 2 diabetes mellitus (T2DM) [7–9]. Indeed, PA, and improved levels of CRF, have been shown to beneficially impact known NCD risk factors, such as insulin resistance and hypertension [10, 11]. In particular, aerobic exercise, defined as a repetitive and rhythmic activity that engages the larger muscle groups in the body [12], has been shown to play an important role and can improve CRF, an indicator of overall cardiopulmonary efficiency [13]. Moreover, aerobic PA can contribute to maintenance of mobility, by increasing bone and muscle strength, preventing illness, and stabilising metabolic health [14]. Despite the evidence in favour of regular PA, the World Health Organization (WHO) estimates that 27.5% of the world’s adult population do not meet the recommended levels of PA [15]. Moreover, older adults tend to be more sedentary and engage in less PA than younger population groups, and PA is not yet a fully realised aspect of geriatric or preventative care among older adults [16, 17].

Established guidelines for PA in older adults tend to be based on health recommendations for the prevention of NCDs in the general adult population [18, 19]. These recommendations, and the evidence they are based on, may not be universally applicable to older adults, due to the increased presence of chronic multimorbidity, differences in functional abilities, and the biological ageing process, including the accumulation of life course risks [20, 21]. However, there is a growing body of evidence, including two (2) recent systematic reviews [22, 23], suggesting that PA and exercise interventions positively contribute to well-being, quality of life and physical functional ability among older adults, for example, living in long-term care facilities (LTCF) [22, 23]. As a result, consensus statements [24] and taskforce reports for older adults in LTCF [21] from international expert groups have called for the use of individualised, tailored PA and exercise programmes for disease prevention, management, and improvement of functional status in older adults.

Evidence for an impact of PA on mortality [25] and NCD outcomes among older adults has been less conclusive, with a 2017 systematic review in ≥ 70-year-olds finding positive effects of aerobic PA interventions in outcomes such as CRF, quality of life, and glucose metabolism; but inconclusive results for MetS, blood pressure measures, or body composition [14]. Furthermore, the optimal intensity, frequency and duration of aerobic exercise among older adults remains unknown [14], and is unlikely to be a “one-size-fits-all” dose-response. Therefore, a better understanding of the impact of aerobic PA on specific NCDs and mortality outcomes is needed, to help integrate, individualise, and adjust the use of PA as a preventative and treatment modality in older adults. As an update and expansion of existing reviews, and to identify remaining evidence gaps, this systematic review assessed the impact of and associations between aerobic PA and CRF on mortality and NCD outcomes in those aged ≥ 65 years, including both intervention studies and observational epidemiological evidence published since 2016.

## Methods

### Study design

The systematic review was guided by and reported in accordance with the PRISMA statement [26] (Supplementary File 1). We searched for articles in Scopus, Cochrane Library, PubMed and Web of Science, that reported the effects of CRF and/or aerobic physical activity on mortality and common NCDs (CVD risk, obesity, dysglycaemia, dyslipidaemia, hypertension, MetS, depression, cancer, and frailty and/or falls) among older adults.

### Eligibility criteria

Studies evaluating the impact of or association between CRF and/or aerobic PA on mortality and common NCDs in participants over 65 years old were included in this review. In terms of study design, both intervention (randomised controlled trials, RCTs) and observational studies (cohort, cross-sectional, and case-control studies) were included. Studies including participants < 65 years old and not stratifying results by age to allow for the extraction of data for participants ≥ 65 years only, were excluded. Studies in a language other than English, as well as letters, commentaries, reviews, mathematical modelling studies, editorials, and animal studies were excluded.

Aerobic PA was defined as any exercise involving movement of large muscle groups for a period of time (treadmill walking/running, jogging, cycling, rowing, dancing) [12], and included both objectively measured PA, through accelerometery, and subjectively self-reported measures using the International or Global Physical Activity Questionnaires (IPAQ, GPAQ). Aerobic PA included any form of movement including during leisure time, for transport to get to and from places, or as part of a person’s work. Studies using an aerobic PA intervention were additionally included. No specific threshold was set for inclusion in terms of the frequency, duration, and intensity of training session. Where applicable, we used each study’s definition of vigorous PA (VPA), moderate PA (MPA), moderate-vigorous (MVPA), and low or light-intensity PA (LPA), usually defined as less strenuous activity that does not cause sweating or shortness of breath, or based on a MET level of 1.6–2.9 kcal/kg/h [27]. We excluded studies solely exploring the impact of non-aerobic activities such as strength training, yoga, Pilates, and stretching. We also excluded intervention studies without a control group (for example, those comparing aerobic PA to combined aerobic PA and diet). CRF measures included estimated VO2max based on the exercise treadmill, cycle ergometry, 6-minute walking test (6MWT) or the 2-minute step test.

Studies reporting on any of the following outcomes were eligible for inclusion: (all cause) mortality; cardiovascular events and risk (heart failure, coronary heart disease, stroke, cardiovascular risk scores, etc.); obesity or adiposity (including body mass index [BMI], body fat mass [FM], visceral adiposity, waist circumference [WC], body fat percentage [BF%]); hypertension (including systolic blood pressure [SBP], diastolic blood pressure [DBP], mean arterial pressure); dysglycaemia and glucose metabolism (including fasting glucose, glycosylated haemoglobin [HbA1c], insulin resistance, insulin sensitivity, and T2DM); dyslipidaemia (including total cholesterol [TC], high-density-lipoprotein cholesterol [HDL-c], low-density-lipoprotein cholesterol [LDL-c], triglycerides [TG]); MetS; depression; frailty and/or falls; and cancers.

### Search strategy

A search strategy that employed medical subject headings (MeSH) and keywords was developed and used while searching for literature from selected databases. The following key terms were used in the search strategy; “cardio-respiratory fitness”, “aerobic physical activity”, “hypertension”, “high blood pressure”, “high cholesterol”, “diabetes”, “mental health”, “depression”, “cardiovascular disease”, “cancer”, “frailty or falls”, “mortality”, and “adults or elderly”. Boolean terms (AND; OR) were used to separate keywords, and MeSH was conducted in advanced searching of articles. The final search strategy for PubMed can be found in Supplementary Table 1. The same search strategy was adopted and tailored for use for each database. A secondary search of relevant articles from reference lists of included studies was also undertaken. The initial search was conducted in the proposed databases from January 2016 until February 2024.

### Study screening

Retrieved articles were uploaded into Rayyan [28, 29], and duplicates were removed. The articles were then independently screened based on title and abstract by at least two reviewers per study (CA, AM, JR, ZM, LMS). The full text of studies that passed the initial stage of screening were retrieved and full texts were screened in duplicate by two reviewers to verify their conformance with the inclusion criteria. Any uncertainty or disagreements on whether selected studies should be included or not were discussed and resolved by group consensus between CA, AM, JR, ZM, and LMS.

### Data extraction and synthesis

For all included manuscripts, three reviewers extracted data using a data extraction tool designed for this study (Supplementary Table 2). For studies measuring multiple outcomes of interest, data extraction was performed individually for each outcome. The following information was extracted: Name of first author and year of publication, study location (country), study population (sample size, age, gender), study design, data collection period, exposure type (CRF or PA, observed or intervention), outcome measurement method, main findings in terms of measure of association used (risk ratio [RR], odds ratio [OR], hazard ratio [HR], rate ratio, mean/median difference). All results compatible with each outcome domain were extracted per study; any missing or unclear information pertaining to study characteristics was reported as such and taken into account during the risk of bias (RoB) assessments. To ensure a comprehensive overview, all manuscripts that met the inclusion criteria, irrespective of assessed quality, were included in this systematic review. Articles whose full text could not be obtained were excluded if the authors could not be reached.

A narrative approach was used for data synthesis with studies grouped, first by outcome measured, and then by exposure (CRF or PA). Meta-analysis was not performed because of the heterogeneity of the PA exposure and of the various outcomes. Effect measures of interest included RR, mean difference (between groups), HR, OR, and linear regression coefficients, and iso-temporal substitution model results. Where applicable per outcome, we additionally included a synthesis of within-study comparison of age groups > 65 years (e.g. 65–75 vs. ≥ 75), if this was reported by included studies. The term “some evidence” is used to reflect variability in associations across and within studies, including differences related to statistical adjustment, between group differences, different outcome measures, PA intervention and type, and/or participant characteristics such as age, sex, and comorbidities.

### Risk of bias and quality assessment

Following study selection, a detailed RoB assessment was performed using the Cochrane Collaboration RoB tool 2.0 [29] for RCTs and the Newcastle-Ottawa scale (NOS) for non-randomised studies [30]. We rated the quality of studies (good, fair, poor) based on the NOS guidelines. A “good” quality score required 3 or 4 stars in selection, 1 or 2 stars in comparability, and 2 or 3 stars in outcomes. A “fair” quality score required 2 stars in selection, 1 or 2 stars in comparability, and 2 or 3 stars in outcomes. A “poor” quality score reflected 0 or 1 star(s) in selection, or 0 stars in comparability, or 0 or 1 star(s) in outcomes [31]. RoB assessment was done by CA, AM, JLR, ZM, with any uncertainties or conflicts discussed between these reviewers and LMS, until consensus was reached.

## Results

**Fig. 1:**
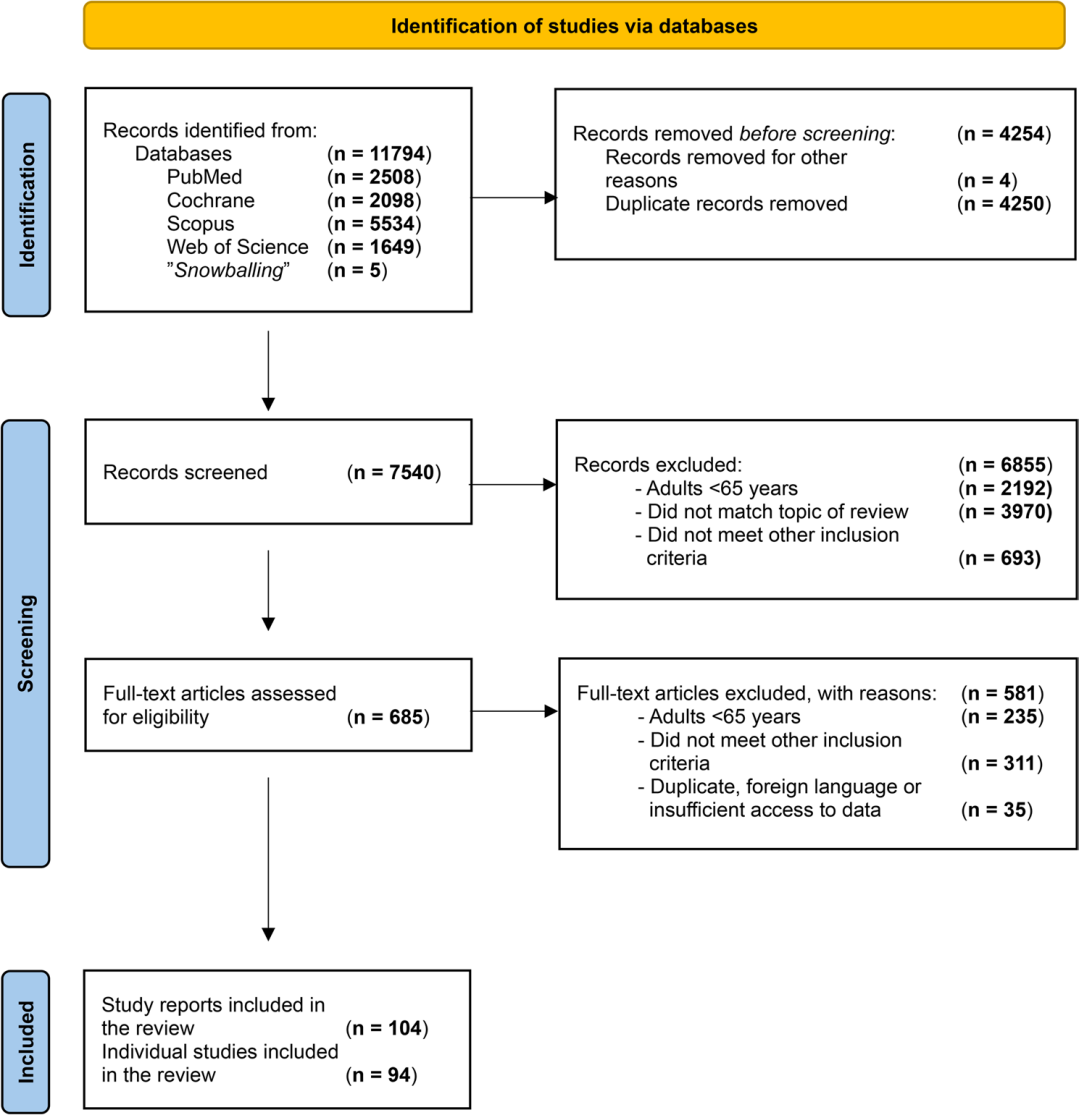
Flow diagram indicating studies identified via searches of databases

**Fig. 2:**
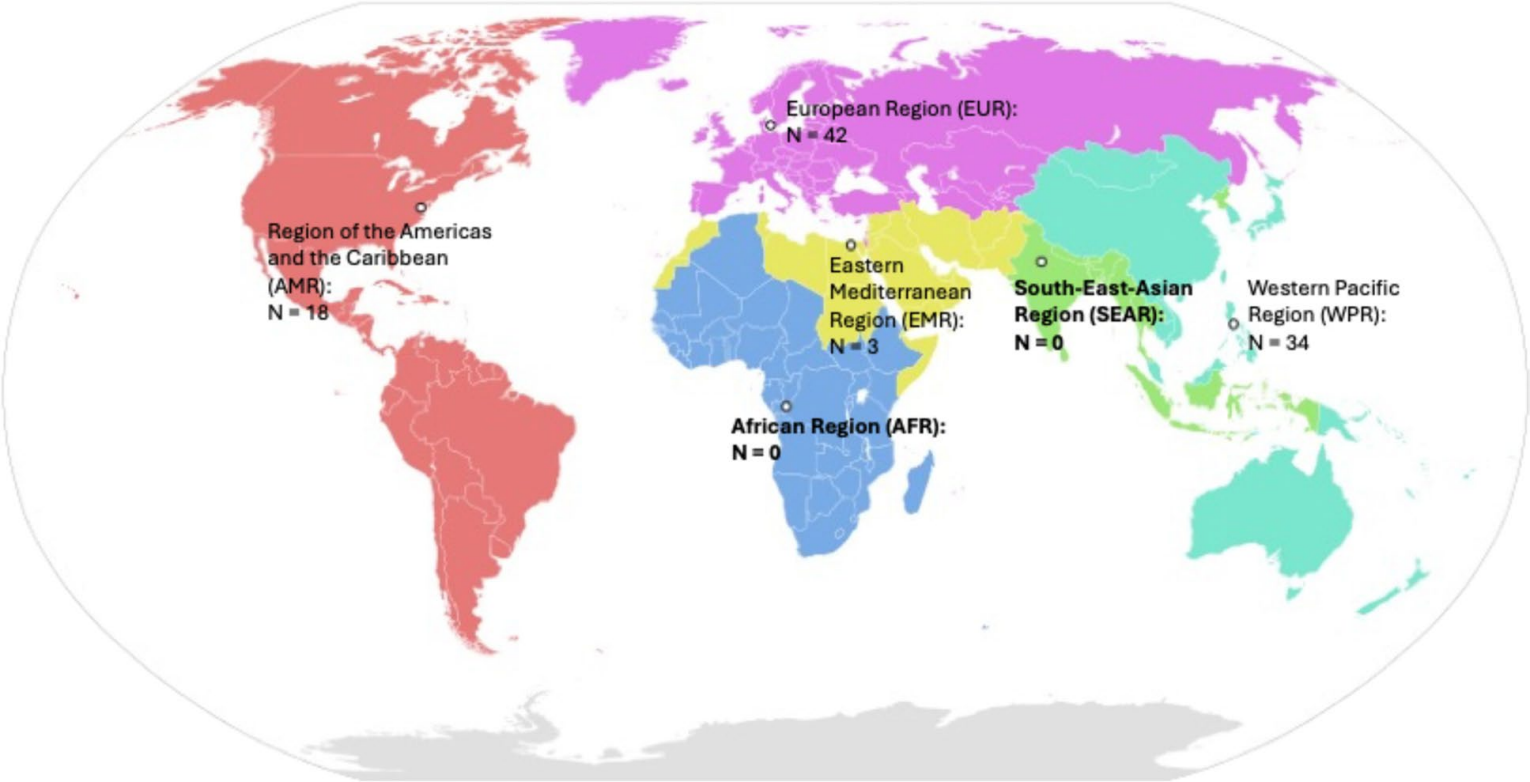
Geographical overview of included studies (*n* = 94). Map: Derfel73; Canuckguy et al., Public domain, via Wikimedia Commons

**Fig. 3:**
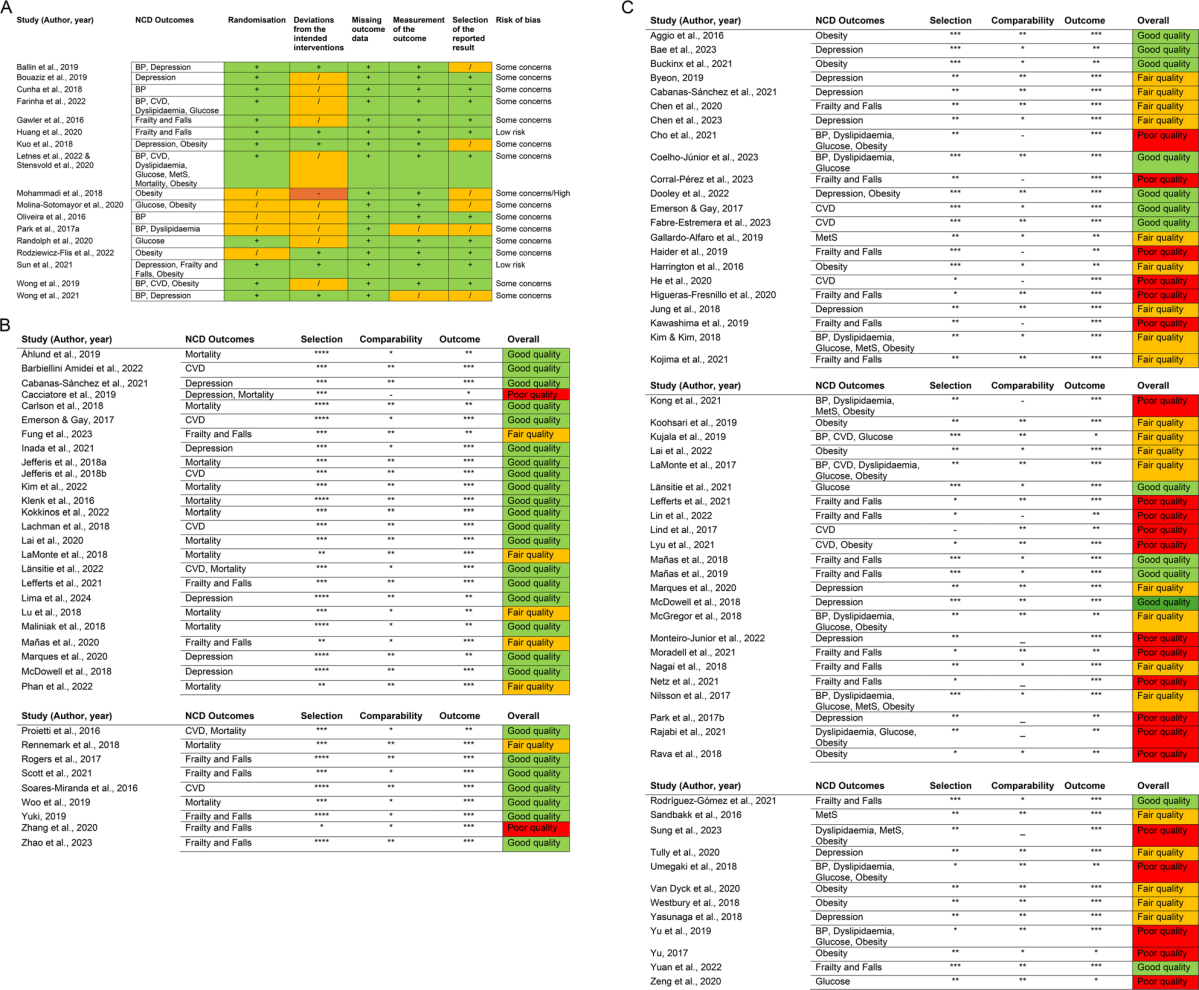
**A **Results from the Risk of Bias assessments (RCT). **B **Results from the Risk of Bias assessments (Cohort). **C **Results from the ROB assessments (Cross-sectional). Good quality: 3 or 4 stars in selection domain AND 1 or 2 stars in comparability domain AND 2 or 3 stars in outcome/exposure domain. Fair quality: 2 stars in selection domain AND 1 or 2 stars in comparability domain AND 2 or 3 stars in outcome/exposure domain. Poor quality: 0 or 1 star in selection domain OR 0 stars in comparability domain OR 0 or 1 stars in outcome/exposure domain.

The search resulted in 11794 manuscripts identified, of which 4250 were duplicates, and 4 Cochrane Clinical Answers or Special Collection publications. The remaining 7540 studies were screened on the basis of the title and abstract, with 6855 records being excluded based on the reasons listed in Fig. 1, such as wrong study design and wrong exposure or outcome. Subsequently, 685 manuscripts were retrieved and subjected to a full text review, including 5 through snowball search procedures, resulting in 104 manuscripts [32–135], reporting on 94 individual studies (Fig. 1). Table 1 provides a summary overview of these included studies. No studies exploring the association between PA/CRF and cancer in populations ≥ 65 years were identified. The majority of included studies were cross-sectional in design (*n* = 54); 7 of these studies additionally reported a prospective analysis for at least one outcome of interest. In terms of regions represented, the majority of studies took place in the European region, American region, and West Pacific region (including Japan, Korea, and China), with only 3 studies from the East Mediterranean region (Israel and Iran) (Fig. 2). There were no studies representing the African or South East Asian regions. Ten (10) of the included studies enrolled participants with comorbidities, which included heart disease, existing obesity, breast cancer, and frail participants admitted to hospital [32, 33, 43, 61, 66, 71, 75, 92, 102, 119, 121]. The majority of studies reporting objectively measured PA used accelerometery (*n* = 70), while the 34 studies used self-report. Results are presented in detail per outcome below and in Tables 2, 3, 4, 5, 6, 7, 8, 9 and 10. The RoB assessments can be found in Fig. 3A, B and C, and are summarised per outcome, below.

**Table 1:** Overview of included studies (*n* = 94)

	Study design			Sample size			Outcome									WHO Region						Self-reported PA	In comorbid population
	Cross- sectional	Longitudinal Cohort	RCT/ intervention	< 100	100–500	> 500	Mortality	CVD events/ risk	Obesity	Dysglycaemia	Dyslipidaemia	BP/Hypertension	MetS	Depression	Frailty/ Falls	EUR	AFR	AMR	SEAR	EMR	WPR		
Aggio et al., 2016 [62]	X					X			X							X						-	-
Åhlund et al., 2019 [32]		X			X		X									X						-	X
Bae et al., 2023 [98]	X				X									X							X	-	-
Ballin et al., 2019 [92]			X	X								X		X		X						-	X
Barbiellini Amidei et al., 2022 [49]		X				X		X								X						X	-
Bouaziz et al., 2019 [99]			X	X										X		X						-	-
Buckinx et al., 2021 [63]	X				X				X									X				X	-
Byeon et al., 2019 [100]	X				X									X							X	X	-
Cabanas-Sánchez et al., 2021 [101]	X	X				X								X		X						-	-
Cacciatore et al., 2019 [33]	X	X			X		X							X		X						X	X
Carlson et al., 2018 [34]		X				X	X											X				X	-
Chen et al., 2020 [112]	X					X									X						X	-	-
Study at National Taiwan University Hospital Chen et al., 2023 [102] Lai et al., 2022 [71]	X				X				X					X							X	-	X
Cho et al., 2021 [64]	X				X				X				X								X	X	-
Coelho-Júnior et al., 2023 [87]	X					X				X	X	X				X						X	-
Corral-Pérez et al., 2023 [113]	X				X										X	X						-	-
Cunha et al., 2018 [93]			X	X								X						X				-	-
Dooley et al., 2022 [65]	X				X				X					X				X				-	-
Emerson & Gay, 2017 [50]	X					X		X										X				-	-
Fabre-Estremera et al., 2023 [51]	X					X		X								X						-	-
Farinha et al., 2022 [52]			X		X			X		X	X	X				X						-	-
Fung et al., 2023 [114]		X				X									X			X				X	-
Gallardo-Alfaro et al., 2019 [96]	X				X								X			X						X	-
Gawler et al., 2016 [115]			X			X									X	X						-	-
Haider et al., 2019 [116] (country-level correlations)	X														X	X						X	-
Harrington et al., 2016 [66]	X				X				X							X						-	X
He et al., 2020 [53]	X				X			X													X	-	-
Higueras-Fresnillo et al., 2020 [117]	X				X										X	X						-	-
TOYOTA Trial Umegaki et al., 2018 [82] Huang et al., 2020 [118]	X		X		X				X	X	X	X			X						X	-	-
Inada et al., 2021 [103]		X			X									X							X	-	-
British Regional Heart Study Jefferis et al., 2018a [35] Jefferis et al., 2018b [54]		X				X	X	X								X						-	-
Jung et al., 2018 [104]	X					X								X							X	-	-
Kawashima et al., 2019 [119]	X			X											X						X	-	X
Kim & Kim, 2018 [67]	X					X			X	X			X								X	X	-
Kim et al., 2022 [36]		X				X	X														X	X	-
Klenk et al., 2016 [37]		X				X	X									X						-	-
Kojima et al., 2021 [120]	X				X										X						X	-	-
Kokkinos et al., 2022 [38]		X				X	X											X				-	-
Kong et al., 2021 [68]	X			X					X		X	X	X								X	-	-
Matsuda cohort Koohsari et al., 2019 [69] Yasunaga et al., 2018 [111]	X				X				X					X							X	-	-
Kujala et al., 2019 [55]	X					X		X		X		X				X						X	-
Kuo et al., 2018 [70]			X	X					X					X							X	-	-
Lachman et al., 2018a [16]		X				X		X								X						X	
Lai et al., 2020 [39]		X				X	X														X	X	-
OPACH Study LaMonte et al., 2017 [57] LaMonte et al., 2018 [40]	X	X				X	X	X	X	X	X	X						X				-	-
Oulu-45 Länsitie et al., 2021 [88] Länsitie et al., 2022 [41]	X					X	X	X		X						X						-	-
Lefferts et al., 2021 [121]	X	X			X										X			X				-	X
Generation 100 randomized trial Letnes et al., 2022 [72] Stensvold et al., 2020 [48]			X			X	X	X	X	X	X	X	X			X						-	-
Lima et al., 2024 [105]		X				X								X		X		X			X	X	-
Lin et al., 2022 [122]	X					X									X						X	-	-
Lind et al., 2017 [58]	X					X		X								X						X	-
Lu et al., 2018 [42]		X				X	X														X	X	-
Lyu et al., 2021 [59]	X					X		X													X	X	-
Maliniak et al., 2018 [43]		X				X	X											X				X	X
Toledo Study for Healthy Aging Mañas et al., 2018 [125] Mañas et al., 2019 [123] Mañas et al., 2020 [124] Rodríguez-Gómez et al., 2021 [129]	X	X			X	X									X	X						-	-
Marques et al., 2020 [106]	X	X				X								X		X						X	-
McDowell et al., 2018 [107]	X	X				X								X		X						X	-
McGregor et al., 2018 [73]	X					X			X	X	X	X						X				-	-
Mohammadi et al., 2018 [74]			X	X					X											X		-	-
Molina-Sotomayor et al., 2020 [75]			X		X				X	X								X				-	X
Monteiro-Junior et al., 2022 [108]	X			X										X				X				-	-
Moradell et al., 2021 [126]	X			X											X	X						-	-
Nagai et al., 2018 [127]	X					X									X						X	-	-
Netz et al., 2021 [128]	X				X										X					X		-	-
Nilsson et al., 2017 [76]	X				X				X	X	X	X	X			X						-	-
Oliveira et al., 2016 [94]			X	X								X				X						-	-
Park et al., 2017a [91]			X	X							X	X									X	-	-
Park et al., 2017b [109]	X			X										X		X						-	-
Phan et al., 2022 [44]		X				X	X											X				-	-
Proietti et al., 2016 [45]		X				X	X	X								X						X	-
Rajabi et al., 2021 [77]	X				X				X	X	X									X		X	-
Randolph et al., 2020 [89]			X	X						X								X				-	-
Rava et al., 2018 [78]	X			X					X							X						-	-
Rennemark et al., 2018 [46]		X				X	X									X						X	-
Rodziewicz-Flis et al., 2022 [79]			X	X					X							X						-	-
Rogers et al., 2017 [130]		X				X									X	X						X	-
Sandbakk et al., 2016 [97]	X					X							X			X						-	-
Scott et al., 2021 [131]		X		X											X	X						-	-
Soares-Miranda et al., 2016 [60]		X				X		X										X				X	-
Sun et al., 2021 [80]			X		X				X					X	X						X	-	-
Sung et al., 2023 [81]	X					X			X		X		X								X	X	-
Tully et al., 2020 [110]	X					X								X		X						-	-
Van Dyck et al., 2020 [83]	X					X			X							X					X	-	-
Westbury et al., 2018 [84]	X				X				X							X						-	-
Wong et al., 2019 [61]			X		X			X	X			X									X	-	X
Wong et al., 2021 [95]			X		X							X		X							X	-	-
Woo et al., 2019 [47]		X				X	X														X	-	-
Yu, 2017 [85]	X					X			X									X				X	-
Yu et al., 2019 [86]	X					X			X	X	X	X									X	X	-
Yuan et al., 2022 [132]	X					X									X						X	X	-
Yuki et al., 2019 [133]		X			X										X						X	-	-
Zeng et al., 2020 [90]	X					X				X											X	X	-
Zhang et al., 2020 [134]		X				X									X	X						X	-
Zhao et al., 2023 [135]		X			X										X						X	X	-

*RCT* randomised controlled trial, *CVD* cardiovascular disease, *BP* blood pressure, *MetS* metabolic syndrome, *PA* physical activity, *EUR* European region, *AFR* African region, *AMR* Region of the Americas and the Caribbean, *SEAR* South-East Asian region, *EMR* Eastern Mediterranean region, *WPR* Western Pacific region, In comorbid population: includes studies enrolling only frail participants/participants with obesity

**Table 2:** Summary of studies reporting on all-cause mortality

Author/ Reference	Country/ Territory	Follow-up (months)	Sex	*N* (additional population description)	Age (years)	Description of intervention/exposure measure	Outcome measured	Main findings	Association PA & outcome
PHYSICAL ACTIVITY INTERVENTION VS CONTROL									
Stensvold et al., (2020) [48] Generation 100 Randomized Trial	Norway	60 (5 years)	M, F	1567 (777 M, 790 F)	70–77	5-year two weekly sessions of (i) High-Intensity aerobic interval training (walking/running, cross-country skiing and aerobics), or (ii) moderate-intensity continuous training (together, these exercise groups formed the “ExComb” group) vs. “control” (advised to follow national PA recommendations)	All-cause mortality from Cause of Death Registry, Norway	No differences found in all-cause mortality at follow-up between the intervention groups. High-intensity exercise was associated with lower risk of mortality compared to the moderate-intensity group (HR 0.49, 95%CI 0.25 to 0.99), but not in a fully adjusted model. (Note, the control group was advised to follow national PA recommendations and performed high levels of moderate-to high intensity exercise.)	**S**
OBSERVATIONAL STUDIES WITH PHYSICAL ACTIVITY EXPOSURE									
Carlson et al., 2018 [34]	USA	263 (22 years)	M, F	9450	≥ 70	Survey of any exercises, sports, or physically active hobbies in the past 2 weeks at baseline; categorised as inactive, insufficiently active, sufficiently active, and highly active.	Mortality obtained from the National Death Index	7.8% of deaths were attributed to inadequate levels of physical activity. Compared to active adults, adults aged 70 and older who were inactive (HR [95% CI]: 1.16 [1.09–1.23]) and/or insufficiently active (HR [95% CI]: 1.12 [1.04–1.20]) had increased risk of premature death, even when excluding those who died in the first 2 years of follow-up. No significant difference in mortality was found between sufficiently vs. highly active groups.	**Y**
Cacciatore et al., 2019 [33]	Italy	Mean 44.1 months, SD 20.7	M, F	300 (224 M, 76 F) participants with symptomatic Heart Failure	≥ 65	Self-reported PA before the onset of heart failure, using Scale for the elderly (PASE) – categorised into tertiles	Mortality among hospitalised participants	Patients who died had a significantly lower PASE score (86.0 [SD 60.6] vs. 20.9 [SD 29.8], *p* < 0.001); in Cox regression, PA predicted mortality independently of sex, age, and other measures of cognitive and functional fitness. Lowest PASE tertiles had a higher hazard rate compared to the highest PASE tertile (HR 7.25, 95% CI 2.7–19.5, *p* < 0.001).	**Y**
Rennemark et al., 2018 [46]	Sweden	132 (11 years), aged 81–96	M, F	2025	> 80	Survey of moderate physical activity during the past 12 months. Dichotomised for Cox regression: MPA ≤1 times / month vs. >/=MPA 2–3 times / month.	Mortality from the Swedish National Death Register	Those still alive at the 11-year follow-up measure were more often physically active at baseline compared to those no longer alive (86.3% active at least once per month vs. 69.3%, *p* < 0.001). Among older participants (80–96 years), the risk of dying among those who engaged in MPA once per month or fewer was 29% lower (RR = 0.71, 95% CI 0.61–0.82, *p* < 0.01) compared to those engaged in MPA 2–3 times per month or more.	**Y**
Proietti et al., 2016 [45] EURP-AF	9 European countries	12	M, F	835 participants with atrial fibrillation	≥ 75	Survey of PA over past 2 years, categorised as none, occasional, regular, or intense by time spent irrespective of activity	Mortality from physician’s letters, hospital files, report	Among participants > 75 years, higher event rates of all-cause death among patients in the “No PA” category (16.3% vs. 6.2% in occasional; 3.5% in regular; and 4.8% in intense PA), *p* < 0.001.	**Y**
Lu et al., 2018 [42]	China	84 (7 years)	M, F	1242 (754 M, 488 F)	69–94	PA Scale for the elderly (PASE) - quartiles	Mortality from Hong Kong Death Registry	Being least active was significantly associated with risk of all-cause mortality (HR 3.49, 95% CI 1.92–6.37) compared to highest PA quartile; still significant in the fully adjusted model (HR 2.93, 95% CI 1.58–5.42); A significant linear relationship was found between higher levels of PA and increased mortality risk in the unadjusted model and fully adjusted model (*p* < 0.05). Compared with those physically active plus cardiorespiratory fit, physically inactive plus unfit people had the highest risk of all-cause mortality.	**Y**
LaMonte et al., 2018 [40] OPACH Study	USA	Mean 37.2 (3.1 years; range 0.5–4.5)	F	3171	≥ 80; 80–89 vs. ≥ 90	Accelerometer (ActiGraph); low-light, high-light, MVPA	All-cause mortality from outcome questionnaire, national death index, obituaries, hospital records	Among women ≥ 80 years, the relative risk of overall mortality was lower with 30-minutes per day increase in total PA, LPA, and MVPA after adjusting for relevant mortality predictors and covariates. Absolute rates of all-cause mortality were 44% and 15% lower, respectively, when comparing the middle and lowest total PA tertile. No difference in results for the subgroup aged ≥ 90 vs. 80–89-year age group.	**Y**
Lai et al., 2020 [39]	Taiwan	72 (6 years)	M, F	42,047	≥ 65	Survey asking about exercise > 20 min in the past 6 months.	Mortality from Taiwan National Registration of Death	Older people with higher exercise frequency had a significantly decreased risk of mortality (exercise 1–2 times/week HR = 0.74, 95% CI 0.68–0.81, exercise 3–5 times/week HR = 0.65, 95% CI 0.59–0.70) after adjusting for the potential confounders. This remained significant after excluding 526 people who died within the first year of follow-up. Among those who did not exercise, 22.6% died, compared to 13.6% among those who exercised 1–2 times per week, and 11.3% of those who exercised 3–5 times per week.	**Y**
Kim et al., 2022 [36]	Korea	Mean 39.6 SD 14 (3.3 years)	M, F	58,537 (21057 M, 37480 F)	≥ 65	Self-report questionnaire using the 7-day recall method; sedentary, MVPA and LPA, MVPA only, LPA only	All-cause mortality from Korea National Statistical Office	When stratified by PA intensity, the crude death rate (per 1000 person-years) was 40.5, 22.6, 19.0, and 16.2 in the total sedentary, LPA only, LPA and MVPA, and MVPA only groups, respectively. Even LPA 1x/week was associated with significantly lower risks of all-cause mortality than the total sedentary group. A non-linear relationship between LPA and mortality-risk showed a continuous decrease in mortality risk until 360 MET-min/week (120 min/week: HR 0.63, 95% CI 0.57–0.71) followed by a plateau. For the same exercise duration, MVPA was associated with lower all-cause mortality than LPA.	**Y**
Länsitie et al., 2022 [41] Oulu45 cohort	Finland	Mean 6.2 years	M, F	660 (277 M, 383 F)	67–70	Accelerometer (Polar Active), MVPA, LPA	All-cause mortality from Digital and Population Data Services Agency, Finland	In Cox regression, every 10 min increase in LPA was associated with 6.5% lower mortality risk (HR 0.935, 95% CI 0.884–0.990, *p* = 0.020), this remained significant when adjusting for sex, WC, and Framingham risk score. No significant association was found between MVPA and mortality.	**S**
Jefferis et al., 2018a [35] British Regional Heart Study	UK	Median 60 (5 years, range 0.2–6.1)	M	1181	71–92	Accelerometer (ActiGraph); LPA, MVPA, steps per day.	All-cause mortality from British National Health Service central registers	For each additional 30 min in LPA or 10 min in MVPA, HRs for all-cause mortality were respectively 0.83 (95% CI 0.77–0.90) (Table 3) and 0.90 (95% CI 0.84–0.96). For each additional 1000 steps/day, the HR was 0.84 (95% CI 0.78–0.91). No changes after adjustment for covariates. LPA remained significant upon mutual adjustment for each PA intensity. There was a dose-response across quartiles of activity, with lower risk in higher quartiles of MVPA and steps. Accumulation of bouts lasting ≥ 10 min did not appear important beyond accumulating 150 min MVPA/week.	**Y**
Klenk et al., 2016 [37]	Germany	48 (median 4 years)	M, F	1271 (717 M, 554 F)	≥ 65	Accelerometer; walking duration quartiles	Mortality from the local registration offices	An inverse relationship between walking duration quartiles and mortality was found even after multivariate adjustment (compared to quartile 1, HR quartile 2: 0.45, 95% CI 0.26–0.76; HR quartile 3: 0.18, 95% CI 0.08–0.41; HR quartile 4: 0.39, 95% CI 0.19–0.78), respectively.	**Y**
Maliniak et al., 2018 [43]	USA	7.5, 9.1 years for PA measured before/after diagnosis, respectively	F	4226; breast cancer survivors	≥ 65	Self-report survey of recreational MVPA	All-cause mortality and breast cancer-specific mortality among older breast cancer survivors from National Death Index	Pre- and post-diagnosis physical activities were significantly associated with reduced all-cause mortality (*p* < 0.05). Associations were stronger for postdiagnosis PA (PA measured after breast cancer diagnosis). Pre- and post-diagnosis physical activities were not associated with breast-cancer specific mortality among women aged 65 years or older.	**S**
OBSERVATIONAL STUDIES WITH CARDIO-RESPIRATORY FITNESS EXPOSURE									
Åhlund et al., 2019 [32]	Sweden	12	M, F	390 (169 M, 221 F); frail elderly adults in need of emergency care	≥ 75	6-minute walk test (6MWT); category: walking distance < 100 m vs. > 200 m	All-cause mortality from National Registry of Records, Sweden	After adjustment, 6MWT category was associated with 1-year mortality (HR 3.31, 95% CI 1.89–5.78, *p* < 0.001) for 1-year mortality. Change in 6MWT was also associated with mortality, where patients who performed worse after 3 months had higher mortality (HR 3.80, 95% CI 1.42–10.06, *p* = 0.007).	**Y**
Woo et al., 2019 [47]	China/Hong Kong	84 (7 years)	M, F	1176 (709 M, 467 F)	≥ 65	6-minute walk test (6MWT): 6MWD: 6-minute walking distance; 6MWS: 6-minute walking speed; peak oxygen uptake using cycle ergometer	All-cause mortality from Hong Kong Death Registry	For both men and women, those who died had lower VO2peak (women: 19.5 [SD 3.8] vs. 17.1 [SD 3.6]; *p* < 0.001; men: 22.5 [SD 4.4] vs. 20.3 [SD 4.6]; *p* < 0.0001), 6MWD (women: 412.3 [SD 66.5] vs. 367.1 [SD 71.2]; *p* < 0.001; men: 466.1 [SD 77.1] vs. 415.2 [SD 97.3]; *p* < 0.001), and 6MWS (women: 0.91 [SD 0.19] vs. 0.81 [SD 0.19]; *p* < 0.0001; men: 1.04 [SD 0.20] vs. 0.94 [SD 0.23]; *p* < 0.001), which remained significant after adjustment for covariates.	**Y**
Phan et al., 2022 [44]	USA	Mean 42 (3.5 years), retrospective	M, F	14,499	≥ 70; 70–79 vs. 80–90	Exercise tolerance test; low fitness METs < 5; moderate fitness METS 5–10; high fitness METs > 10	All-cause mortality from US Social Security Death Index, California State Death Master Files, and Kaiser Permanente Health Plan database	Patients aged 80–90 with low fitness had the highest risk of all-cause mortality (HR 10.6, 95% CI 7.8–14.4, *p* < 0.001); those with the highest fitness had comparable or even lower HR risk (HR 2.9, 95% CI 1.2–7.2, *p* = 0.02) compared with their younger counterparts aged 70–80 years with moderate or low fitness, and better survival compared with patients aged 60–70 years with low fitness.	**Y**
Kokkinos et al., 2022 [38] ETHOS study	USA	Median 122.4 (10,2 years)	M, F	137,626 army veterans	≥ 70; 70–79 vs. 80–95	Treadmill speed and grade; categorised into 6 categories (least fit, low fit, moderately fit, fit, extremely fit)	All-cause mortality from Veterans Affairs Beneficiary Identification Records Locator Subsystem	Mortality risk decreased with increasing level of CRF; in those aged 70–79: extremely fit participants had 19.3% mortality compared to 57.4% mortality among least fit participants (HR 0.34, 95% CI 0.32–0.37); in those aged 80–95: extremely fit participants had 40.6% mortality compared to 75.7% among least fit participants (HR 0.27, 95% CI 0.24–0.29)	**Y**

*N* sample size, *PA* physical activity, *Y* yes, *S* some, *N* no, *M* male, *F* female, *PASE* Physical Activity Scale for the Elderly, *IPAQ* International Physical Activity Questionnaire, *GPAQ* Global Physical Activity Questionnaire, *LPA* light physical activity, *MPA* moderate physical activity, *VPA* vigorous physical activity, *MVPA* moderate to vigorous physical activity, *6MWT* 6-minute walk test, *UK* United Kingdom, *USA* United States of America, *HR* hazard ratio, *95% CI* 95% confidence interval, *SD* standard deviation

**Table 3:** Summary of studies reporting on CVD risk/events

Author/ Reference	Country/ Territory	Follow-up (months)	Sex	*N* (additional population description)	Age (years)	Description of intervention/exposure measure	Outcome measured	Main findings	Association PA & outcome
PHYSICAL ACTIVITY INTERVENTION VS CONTROL									
Wong et al., 2019 [61]	China	5	F	100 (women with Stage 2 hypertension)	67–85	Intervention: The instructor supervised swimming training program (a combination of freestyle, breaststroke, and backstroke) twice/week. Control group: visited the laboratory at the same frequency for non-PA recreational activities.	PWV, AiX	Significant group x time interactions (*p* < 0.05) for crPWV and Aix. CrPWV decreased and was statistically significantly lower compared to the control group after the intervention (-1.2 [SD 0.2] m/s). Similarly, Aix significantly decreased and was statistically significantly lower compared to the control group after the intervention (-4% [SD 1%]). No significant changes were observed in the control group.	**Y**
Farinha et al., 2022 [52]	Portugal	7	M, F	102 (50 lost to follow-up)	≥ 65	Aquatic exercise Interventions of 45 min twice/week: continuous aerobic (AerG), interval aerobic exercise (IntG), and combined aerobic and muscle strength (ComG). Control: age-matched, no systematic exercise.	IMT	No statistically significant changes in IMT observed after the intervention for any of the groups (*p* > 0.05).	**N**
Stensvold et al., (2020) [48] Generation 100 Randomised Trial	Norway	60 (5 years)	M, F	1567 (777 M, 790 F)	70–77	5-year two weekly sessions of (i) High-Intensity aerobic interval training (walking/running, cross-country skiing and aerobics), or (ii) moderate-intensity continuous training (together, these exercise groups formed the “ExComb” group) vs. “control” (advised to follow national PA recommendations)	CVD events from ICD codes in patient archive system; CV death from Cause of Death Registry, Norway	No differences observed for CVD events or death between the intervention groups. (Note, the control group was advised to follow national PA recommendations and performed high levels of moderate-to high-intensity exercise.)	**N**
OBSERVATIONAL STUDIES WITH PHYSICAL ACTIVITY EXPOSURE									
Fabre-Estremera et al., 2023 [51] Seniors-ENRICA-2	Spain	Cross-sectional	M, F	1939 (860 M, 1079 F) without major cardiovascular disease	≥ 65	Accelerometer (ActiGraph); LPA, MVPA	Cardiovascular disease risk using high-sensitivity cardiac troponin T (hs-cTnT) and N-terminal pro-brain natriuretic peptide (NT-proBNP) biomarkers of myocardial infarction and heart failure.	Overall, the association between PA and cardiac markers differed by the presence of subclinical cardiac damage, level of PA, and sex. In less active individuals with subclinical cardiac damage, PA was associated with lower hs-cTnT ad NT-proBNP.	**S**
Jefferis et al., 2018b [54] British Regional Heart Study	UK	Median 4.9 years	M	1181	71–92	Accelerometer (ActiGraph)	Fatal or non- fatal myocardial infarction, stroke or heart failure event (herein referred to as CVD) from hospital records and British National health Services Central Registers.	The risk of CVD events was lower in the higher quartiles of steps and MVPA, even in the fully adjusted model (total steps, quartile 4: HR 0.34, 95% CI 0.17–0.67, *p* < 0.05; MVPA, quartile 4: HR 0.34 95% CI 0.16–0.74, *p* < 0.05). Although associations were found between CVD events and LPA, these were no longer significant when adjusting for MVPA and additional covariates.	**S**
Proietti et al., 2016 [45] EURP-AF	9 European countries	12	M, F	835 participants with atrial fibrillation (admitted and outpatient)	≥ 75	Survey of PA over past 2 years, categorised as none, occasional, regular, or intense by time spent irrespective of activity	CV death, stroke/TIA, Bleeding, thromboembolic event; from self-report, physician’s letters, or hospital discharge summaries.	Cardiovascular death was significantly higher in the group doing no PA vs. occasional, regular, or intense PA (7.0% vs. 1.5%, 1.2%, 0%, respectively; *p* = 0.002); no significant difference across PA groups was found for stroke/TIA, Bleeding, or TE. In regression analysis, doing occasional PA resulted in lower odds of the combined outcome of CV death/thromboembolic event/bleeding (OR 0.46, 95% CI 0.26–0.83, *p* = 0.008), but no significant association was found for regular or intense PA in this age.	**S**
Lachman et al., 2018b [56] EPIC Norfolk	UK	Median 18.0 years in entire population (aged 39–79)	M, F	7467	> 65	Self-reported using short EPIC PA questionnaire; categorised into 4 levels: active, moderately active, moderately inactive, inactive.	CVD events from ICD-10 codes of hospitalisation or death records using the East Norfolk Health Authority (ENCORE) database	The adjusted HR for CVD events according to Cox regressions was 0.86 (95% CI 0.78–0.96), 0.87 (95% CI 0.77–0.99), and 0.88 (95% CI 0.77–1.02) for moderately inactive, moderately active, and active participants, respectively, when compared to inactive participants and adjusted for age, sex, lifestyle and metabolic risk factors.	**Y**
Lind et al., 2017 [58] PIVUS study	Sweden	Cross-sectional	M, F	989	70	Self-reported: physically active= regular heavy exercise for more than 30 min once a week	LVMI, EF, IMT; by obesity status	For LVMI and EF: Compared to physically active/normal weight participants, increased LVMI and reduced EF in both the physically active/obese and non-physically active/obese groups (*p* < 0.05); For IMT: physically active/overweight and non-physically active/obese groups (but not physically active/obese group) had significantly higher IMT compared to the physically active/normal weight group. Overall, an increased level of self-reported physical activity does not fully eliminate CVD risk in the presence of overweight/obesity.	**S**
Barbiellini Amidei et al., 2022 [49]	Italy	8.1–14.1	M, F	2321	≥ 65; PA at 70 vs. 75 vs. 80 vs. 85	Physical activity by self-reported questionnaire; MVPA categorised as ‘inactive’ ≤ 20 min/day and ‘active’ >20 min/day^2^	Incident CVD (CHD, heart failure, stroke)	Active men had significantly lower risk of CVD (HR 0.74, 95% CI 0.58–0.94), CHD (HR 0.66, 95% CI 0.50–0.87) and heart failure (HR 0.72, 95% CI 0.53–0.98), after adjustment for socio-demographics, other health behaviours, and comorbid conditions. (Not significant in women). This risk reduction in men was significant for PA measured at 70–75 years, but not at ≥ 80 years. In men, trajectories of stable-high PA associated with reduced CVD risk compared to stable-low trajectories. In a dose-response analysis for MVPA, a reduction in risk of incident CHD and heart failure was found, greatest for 20–40 min per day (no significant correlation for PA measured at 80 or 85 years).	**S**
Lyu et al., 2021 [59] Northern Shanghai study	China	Cross-sectional	M, F	2830 (1259 M, 1571 F)	≥ 65	Self-report (IPAQ)	Carotid-femoral PWV, CIMT, peripheral artery disease	Weekly walking activity was significantly associated with arterial stiffness (OR 0.75, 95% CI 0.60–0.94, *p* = 0.010), high CIMT (OR 0.70, 95% CI 0.51–0.96, *p* = 0.03) and peripheral artery disease (OR 0.72, 95% CI 0.57–0.91, *p* = 0.005). In addition, walking ≥ 1 h/day (compared to non-walking) was significantly associated with arterial stiffness (0.29, 95% CI 0.15–0.58) and the presence of peripheral arterial disease (0.53, 95% CI 0.28–0.99) after adjustment for confounders. Walking frequency < 3 days/week vs. ≥ 3 days per week) was not associated with CVD markers in the fully adjusted model.	**Y**
LaMonte et al., 2017 [57] OPACH	USA	Cross-sectional	F	2484	≥ 80	Accelerometer (ActiGraph); low-light PA, high-light PA, MVPA	Reynolds Risk Score (RRS) for 10-year predicted CVD risk; RRS ≥ 20	Among women ≥ 80 years, significantly lower adjusted odds of having cardiovascular risk were found for low-light PA (OR 0.89, 95% CI 0.83–0.95), high-light PA (0.76, 95% CI 0.69–0.84), and MVPA (0.73, 95% CI 0.65–0.83). In linear regression, adjusting for covariates and mutually adjusting for each type of PA, low-light, high-light, and MVPA were each negatively associated with RRS.	**Y**
Länsitie et al., 2022 [41] Oulu45 cohort	Finland	Mean 6.2 years	M, F	660 (277 M, 383 F)	67–70	Accelerometer (Polar Active), MVPA, LPA	Framingham Risk Score (FRS)	An increase in MVPA and LPA (by 10 min) was negatively associated with FRS (β= -0.779, 95% CI -1.186, -0.371, *p* < 0.001; and β= -0.293, 95% CI -0.448, -0.138, *p* < 0.001, respectively). However, after adjusting for waist circumference, only sedentary time remained significantly associated with FRS.	**S**
Kujala et al., 2019 [55]	Finland	Cross-sectional	M, F	779 (378 M, 401 F; included 276 twin pairs)	71–75	Accelerometer (Hookie); MVPA, daily steps Self-reported disease restricting mobility	Self-reported questionnaire of diseases, including coronary heart disease and heart failure	MVPA was lower in those with coronary heart disease compared to those without (25 [IQR 18–32] vs. 36 [IQR 34–38], *p* < 0.001), after adjusting for age and sex; MVPA was also lower among participants with heart failure vs. without (18 [IQR 18–32] vs. 36 [IQR 34–38], *p* < 0.001). Similarly, daily steps were statistically significantly lower in those with coronary heart disease or heart failure than those without.	**Y**
Soares-Miranda et al., 2016 [60] CV Health Study	USA	Max 120 (10 years)	M, F	4207 (1641 M, 2566 F)	≥ 65; 65–74 vs. ≥ 75	Self-reported (Minnesota Leisure-Time Activities questionnaire) exercise intensity (using prior 2 weeks);	CVD event from medical records, consisting of: MI, coronary heart disease, Stroke; walking habits including speed.	Greater leisure-time activity and exercise intensity were associated with lower risk of coronary heart disease, stroke and CVD; Results were generally similar when stratified by sex and age group (65–74 vs. ≥ 75), although in the ≥ 75 group only moderate and high (not low) exercise intensity significantly reduced risk of stroke compared to no exercise. Greater walking pace, distance, and overall walking score were each associated with a lower risk of CVD; Compared with a pace < 2 mph, those that habitually walked at a pace above 3 mph had 50%, 53% and 50% lower risk of coronary heart disease, stroke, and CVD respectively. In the ≥ 75 group, only the highest walking distance (≥ 49 blocks/week) was associated with lower coronary heart disease compared to 0–5 blocks/week.	**S**
Emerson & Gay, 2017 [50] NHANES 2003; 2005	USA	Cross-sectional	M, F	1171	≥ 65; 65–74 vs. ≥ 75	Accelerometer (ActiGraph), LPA, MVPA	Framingham Risk Score (10 years)	MVPA, but not LPA, was significantly and negatively associated with CVD risk (B -0.04, *p* = 0.041). No significant interaction between physical activity and ethnicity was found for CVD risk. No significant difference in the effect of MVPA on CVD risk by age subgroup (65–74 vs. ≥ 75).	**S**
OBSERVATIONAL STUDIES WITH CARDIO-RESPIRATORY FITNESS EXPOSURE									
He et al., 2020 [53]	China	Cross-sectional	M, F	257 (127 M, 130 F)	≥65	Cardiopulmonary exercise test (CPET) for peak oxygen update (VO2 max) on a bicycle ergometer (VO2 max); maximal metabolic equivalent (MET max).	Coronary artery disease ≥ 50% vs. coronary artery stenosis < 50%, hospital records	Participants with coronary artery disease had significantly lower VO2 max compared to those without (0.98 [IQR 0.85–1.17] vs. 1.27 [IQR 1.13–1.55], *p* < 0.001). VO2max was significantly associated with coronary artery disease (OR 0.003, 95% CI 0.001–0.014, *p* < 0.001). However, when adjusting for skeletal muscle index, VO2max was no longer statistically significant (*p* > 0.05)	**S**

*N* sample size, *PA* physical activity, *Y* yes, *S* some, *N* no, *M* male, *F* female, *CVD* cardiovascular disease, *CHD* chronic heart disease, *TIA* transient ischemic attack, *FRS* Framingham Risk Score, *RRS* Reynolds Risk Score, *CIMT* carotid intima-media thickness, *IMT* intima media thickness, *EF* ejection fraction, *LVMI* left ventricle mass index, *PWV* pulse wave velocity, *Aix* augmentation index, *IPAQ* International Physical Activity Questionnaire, *GPAQ* Global Physical Activity Questionnaire, *LPA* light physical activity, *MPA* moderate physical activity, *VPA* vigorous physical activity, *MVPA* moderate to vigorous physical activity, *MET* metabolic equivalent of task, *UK* United Kingdom, *USA* United States of America, *OR* odds ratio, *HR* hazard ratio, *95% CI* 95% confidence interval, *SD* standard deviation

**Table 4:** Summary of studies reporting on obesity/adiposity

Author/ Reference	Country/ Territory	Follow-up (months)	Sex	*N* (additional population description)	Age (years)	Description of intervention/exposure measure	Outcome measured	Main findings	Association PA & outcome
PHYSICAL ACTIVITY INTERVENTION VS CONTROL									
Kuo et al., 2018 [70]	Taiwan	2	M, F	36 (6 M, 30 F)	≥65	30-minute prescribed stepper walking program twice per week for 8 weeks. Control: usual routine activities	BMI, WtHR, FM,	The intervention group showed a decrease in BMI (F = 4.58, *p* = 0.04), WtHR [F = 4.60, *p* = 0.04], FM [F = 4.61, *p* = 0.04] from baseline to post-intervention, and these changes were statistically significantly different from the control; the control group showed a non-significant increase from baseline to follow-up for the same indicators.	**Y**
Sun et al., 2021 [80]	Taiwan	3	M, F	122 (27 M, 95 F)	≥65	Music therapy with physical activity consisting of warm-up followed by main body movement (cardiorespiratory endurance using the 2-minute step test) vs. control	BMI	The intervention had no significant effect on BMI (F = 0.000, *p* = 0.997). There was no significant difference in BMI between the pre- and post-test results in the intervention group; in the control group, there was a significant increase in mean ± SD from 23.82 ± 5.10 to 23.90 ± 5.09 (t = 3.113, *p* = 0.003).	**N**
Rodziewicz-Flis et al., 2022 [79]	Poland	3	F	30	65–82	50 min of dance training to Polish folk music, 3 times a week for 12 weeks. Each training session lasted for 50 min and included a 10 min warm-up, 30 min of folk-dance training and fitness exercises vs. control	BMI, BW, FFM, FM (using BIA)	No significant differences were found between groups for BMI and body composition outcomes. Repeated measures (pretest to post-test) showed no differences between the groups.	**N**
Wong et al., 2019 [61]	China	5	F	100 (women with Stage 2 hypertension)	67–85	Instructor supervised swimming training program (combination of free style, breaststroke, and backstroke) for twice/week vs. control: visited the laboratory at the same frequency for non-PA recreational activities	BMI; BW, FM, BF%, (BIA)	Significant group x time interaction found for FM, BF%. Statistically significant decrease in BF%, FM and increase in FFM in the intervention group (-8 SD 1%, -3.2 SD 1.5 kg, + 2.4 SD 1.7 kg, respectively, *p* < 0.05), statistically different to control after intervention. In the control group, BF% increased (+ 4 SD 1%, *p* < 0.05), with no significant difference between timepoints for FM and FFM. No significant changes between time points were found in weight or BMI in either group.	**S**
Mohammadi et al., 2018 [74]	Iran	2	M	24, (Overweight men)	67–77	Intervention: three aerobic exercise sessions of 45–60 min per week, with the intensity of sessions gradually increased to 90 to 95% of the stored maximum heart rate vs. control: continued inactive way of life	BW, BMI, BF%.	Only in the intervention group, statistically significant reduction in: - BW: 78.16 ± 4.80 to 77.67 ± 4.50 (*p* = 0.03); - BMI: 28.43 ± 2.05 kg/m2 to 28.26 ± 2.13 kg/m2 (*p* = 0.02); and - BF%: 44.71 ± 2.38% to 43.16 ± 2.63% (*p* = 0.006). There were significant between-group differences for BW, BMI, and BF% (*p* < 0.05).	**S**
Molina-Sotomayor et al., 2020 [75]	Chile	1.5	F	107	≥65	Walking-based intervention, in elderly women with diabetes and hypoglycaemic treatment vs. control	BMI	In the intervention group, there was a significant decrease in BMI (within-group effect): -1.41 (-1.85-0.96); *p* < 0.001. The difference in BMI pre- and post-intervention was significantly different between the groups (*p* < 0.001)	**Y**
Letnes et al., 2022 [72] Generation 100 Randomized Trial	Norway	60 (5 years)	M, F	1567	70–77	5-year two weekly sessions of (i) High-Intensity aerobic interval training (walking/running, cross-country skiing and aerobics), or (ii) moderate-intensity continuous training (together, these exercise groups formed the “ExComb” group) vs. “control” (advised to follow national PA recommendations)	BMI, WC	Only high-intensity training significantly improved BMI at year 1 (-0.18 kg/m2, 99% CI -0.37, -0, *p* = 0.011) and year 5 of follow-up (-0.24 kg/m2, 99% CI -0.44, -0.04, *p* = 0.002), compared to the control group (of note, control group performed higher levels of unsupervised exercise compared to the moderate-intensity group; significance was assumed at *p* < 0.01). There were no significant group differences in WC at follow-up.	**S**
OBSERVATIONAL STUDIES WITH PHYSICAL ACTIVITY EXPOSURE									
Dooley et al., 2022 [65] Atherosclerosis risk in communities (ARIC) study	USA	Cross-sectional	M, F	459 (185 M, 274 F)	71–92	Accelerometer (ActiGraph): low-light, high light, MVPA	BMI, BMI categories	Participants with overweight had significantly lower minutes of daily low-light intensity activity vs. normal weight participants (β = -20.8, 95% CI -33.3, -8.4). Participants with obesity had significantly lower minutes low-light intensity activity (β = -48.6, 95% CI -62.9, -34.3), lower high-light intensity activity (β = -22.7, 95% CI -32.5, -13.0) and daily steps (β = -589.0, 95% CI -1029.2, -148.9) vs. normal weight participants. No significant differences in total MVPA for overweight or obese vs. normal weight participants.	**S**
Kong et al., 2021 [68]	South Korea	Cross-sectional	M	77	≥65	Accelerometer (Actical), LPA, MPA, MVPA; categorised: low PA (MVPA < 60), moderate PA (MVPA 60–120), and high PA (MVPA > 120)	BW, BF, WC, HC, BF%, FM (impedance scale)	The high PA group showed a significantly lower FM (14.2 kg [SD 3.7] vs. 18.5 [SD 5.1] in low PA) and BF% (24.8% [SD 6.0] vs. 30.6 [SD 6.8] in low PA) than did the low and middle PA groups (*p* < 0.05). However, no significant differences in the PA group were found between those with BF% ≥ 25% vs. < 25% when adjusting for age (*p* > 0.05). No significant differences were observed for BMI.	**S**
Umegaki et al., 2018 [82]	Japan	Cross-sectional	M, F	388 (198 M, 190 F)	65–85	Accelerometer (Lifecorder)	BMI, WC	LPA was negatively associated with WC (-0.162, *p* = 0.001) and BMI (-0.159, *p* = 0.002); total steps were negatively associated with WC (-0.219, *p* = 0.000) and BMI (-0.115, *p* = 0.021); however, differences in MVPA (log) were not statistically significant after adjustment for age and sex. When adjusting for additional significant covariates including MVPA and HDL-C, WC, but not BMI, was significantly inversely associated with LPA (-0.189, *p* = 0.04).	**S**
LaMonte et al., 2017 [57] OPACH	USA	Cross-sectional	F	2484	≥ 80	Accelerometer (ActiGraph); low-light PA, high-light PA, MVPA	BMI	Among women > 80 years, a significant inverse association was found between obesity (BMI ≥ 30) and low-light PA, high-light PA, and MVPA.	**Y**
Westbury et al., 2018 [84]	UK	Cross-sectional	M, F	131 (32 M, 99 F)	74–84	Accelerometer (GENEActiv)	BW, FM (DEXA)	Higher levels of PA were associated with reduced BW (-0.41, 95% CI -0.56, -0.26, *p* < 0.001), BMI (-0.45, 95% CI -0.61, -0.29, *p* < 0.001), and FM (-0.51, 95% CI -0.67, -0.35, *p* < 0.001), after adjustment for gender, age, height, smoking, alcohol and employment type.	**Y**
Rajabi et al., 2021 [77]	Iran	Cross-sectional	M, F	220 (117 M, 103 F)	≥ 65	IPAQ; participants classified into low (< 3.0 METs), moderate (≥ 3.0 METs) or high PA (≥ 6.0 METs) categories.	BMI, BW, WC, HC, WtHR, BF% (skinfold)	WC, HC, and BF% were significantly lower in the high PA group vs. the low PA group in both males and females (*p* < 0.05). No significant differences across PA groups were found for WtHR, BMI, or weight.	**S**
Lyu et al., 2021 [59] Northern Shanghai study	China	Cross-sectional	M, F	2830 (1259 M, 1571 F)	≥ 65	Self-report (IPAQ)	BMI	Compared to those doing no walking activity, participants with walking activity did not have a significantly different BMI (*p* = 0.35).	**N**
Rava et al., 2018 [78]	Estonia	Cross-sectional	F	81	65–91	Accelerometer (ActiGraph)	BW, BMI, FM, BF% (DEXA)	No differences in body composition parameters (BW, BMI, BF%, FM) were observed between the studied groups (using *p* > 0.0025).	**N**
Yu et al., 2019 [86]	China	Cross-sectional	M, F	1744 (885 M, 859 F)	≥ 65	Validated survey of professional and leisure-time physical activity; categorised into light PA, moderate PA, and high PA.	BMI, BMI category	The prevalence of obesity varied but was not statistically significantly different by PA group after adjustment for covariates: Light PA: 41.1% vs. Moderate PA: 39.2% (OR 0.91, 95% CI 0.73–1.13) vs. High PA: 35.0% (OR 0.70, 95% CI 0.73–1.13), *p* > 0.05.	**N**
McGregor et al., 2018 [73] Canadian Health Measure Survey	Canada	Cross-sectional	M, F	1454	65–79	Accelerometer (Actical), LPA, MVPA	BMI, WC	There was a negative association between BMI and time spent in MVPA (coefficient − 0.032, *p* < 0.001) but not LPA. Associations between PA and health indicators weaker in older populations compared to younger adults (18–64 years).	**S**
Aggio et al., 2016 [62]	UK	Cross-sectional	M	1286	70–92	Accelerometer (ActiGraph)	BMI, BMI categories, sarcopenic obesity	In adjusted multinomial logistic regression, MVPA was associated with a reduced risk of (non-sarcopenic) obesity (B = 0.69, 95% CI 0.57–0.84) and sarcopenic obesity (B = 0.47, 95% CI 0.27–0.84).	**Y**
Nilsson et al., 2017 [76]	Sweden	Cross-sectional	F	120	65–70	Accelerometer (ActiGraph)	WC	A significant inverse association between MVPA and WC was observed, independent of sedentary time (β =-1.86 (95% CI -2.60, -1.11) *p* < 0.01). In isotemporal substitution analysis, found a significant increase in WC (*p* < 0.01) when replacing a 10-min time block of MVPA with either LPA or time in sedentary activities.	**Y**
Sung et al., 2023 [81] Korean NHNES 2016–2018	South Korea	Cross-sectional	M, F	893 (220 M, 673 F)	≥ 65	GPAQ: Inactive (0–249 MET min/week), somewhat active (250–499 MET min/week), active (500–999 MET min/week), and very active (> 1,000 MET min/week).	WC, WC>90 cm	Compared to the inactive group, the active group had lower odds of having a large WC (OR 0.40, 95% CI 0.21–0.76, *p* < 0.01). The same association was not significant for the ‘somewhat active’ and ‘very active’ groups.	**S**
Yu, 2017 [85]	USA	Cross-sectional	M, F	7714	≥ 65	Survey of leisure-time physical activity: vigorous, moderate; categorised as meeting sufficient vigorous (75 + min); and moderate (150 + min) activity.	BMI, BMI categories	Older adults were less likely to be obese if they met the recommended level of moderate physical activity (RR = 0.78, 95% CI 0.70–0.86, *p* < 0.001).	**Y**
Koohsari et al., 2019 [69] Matsudo cohort	Japan	Cross-sectional	M, F	297 (186 M, 111 F)	65–84	Accelerometers (Omron)	BMI	Greater LPA and MVPA were associated with lower BMI.	**Y**
Buckinx et al., 2021 [63] YMCA study	Canada	Cross-sectional	M, F	186	≥ 65	Structured interview of PA (resistance training, aerobic training, body and mind practices).- current and in past 5 years	BF%, FM (DEXA)	Reported time spent on aerobic activities currently and in the past 5 years were not significantly associated with any body composition parameters (*p* > 0.05)	**N**
Van Dyck et al., 2020 [83]	China, Belgium	Cross-sectional	M, F	829 (320 M, 509 F)	≥ 65	Accelerometer (ActiGraph)	BMI, BMI categories	Overall, no significant association between MVPA and either BMI or being overweight or obese (*p* > 0.05); In the Hong Kong site only, a significant interaction effect on BMI was found for sedentary time and MVPA.	**N**
Harrington et al., 2016 [66]	UK	Cross-sectional	M, F	100, participants at risk of T2D	≥ 65	Accelerometer (ActiGraph)	FM, android fat mass, gynoid fat mass (DEXA)	Associations between MVPA and FM, android FM, and gynoid FM did not remain statistically significant after adjustment for sex, age, and lifestyle risk factors, group allocation, total lean mass, and sedentary time.	**N**
Kim & Kim, 2018 [67] KNHANES 2010–2012	South Korea	Cross-sectional	M, F	3554	≥ 65	Regular walking, determined by standardised questionnaire as > 30 min per day, > 5 days per week	WC	WC was significantly lower in the “regular walking” group compared to the non-regular walking groups (83.90 ± 0.12 vs. 84.24 ± 0.10, *p* = 0.034).	**Y**
Cho et al., 2021 [64]	South Korea	Cross-sectional	M, F	358	≥ 70	Shortened, Korean version of IPAQ; MVPA	WC, WC ≥ 90 cm in men and ≥ 85 cm in women	For men ≥ 70 years, engaging in < 150 MVPA (compared to engaging in ≥ 150 min of MVPA per week, was significantly associated with odds of having high WC, (OR = 3.42, 95% CI 1.90–6.16); the association was not statistically significant for women.	**S**
Lai et al., 2022 [71] *Study at National Taiwan University Hospital (Chen et al., 2023 [* 102 *])*	Taiwan	Cross-sectional	M, F	199	65–98; 65–74 vs. ≥ 75	Accelerometer; LPA, MVPA	BMI; WC; BF% (BIA)	After adjusting for covariates, reallocating 30 min of sedentary behaviour per day with MVPA was associated with lower BF% (B = -1.408, 95% CI -2.552, -0.264), BMI (B = -0.681, 95% CI -1.300, -0.061), and WC (B = -2.301, 95% CI -4.062, -0.539). Reallocating 30 min of LPA per day with MVPA was also associated with lower WC (B = -2.230, 95% CI -4.173, -0.287). When stratifying by age, these associations were only significant in those aged 65–74, not ≥ 75 years.	**S**

*N* sample size, *PA* physical activity, *Y* yes, *S* some, *N* no, *M* male, *F* female, *WC* waist circumference, *BMI* body mass index, *BF%* body fat percentage, *BW* body weight, *BIA* bioelectrical impedance analysis, *HC* hip circumference, *WtHR* weight to height ratio, *FM* fat mass, *FFM* fat-free mass, *DEXA* dual-energy X-ray absorptiometry, *IPAQ* International Physical Activity Questionnaire, *GPAQ* Global Physical Activity Questionnaire, *PA* physical activity, *LPA* light physical activity, *MPA* moderate physical activity, *VPA* vigorous physical activity, *MVPA* moderate to vigorous physical activity, *UK* United Kingdom, *USA* United States of America, *OR* odds ratio, *HR* hazard ratio, *95% CI* 95% confidence interval, *SD* standard deviation

**Table 5:** Summary of studies reporting on measures of glucose metabolism

Author/ Reference	Country/ Territory	Follow-up (months)	Sex	*N* (additional population description)	Age (years)	Description of intervention/exposure measure	Outcome measured	Main findings	Association PA & outcome
PHYSICAL ACTIVITY INTERVENTION VS CONTROL									
Molina-Sotomayor et al., 2020 [75]	Chile	6	F	107, with diabetes and medication	≥ 65	Walking-based intervention to control group. vs. control group	HbA1c	In the intervention group, there was a significant decrease in Hb1Ac following the intervention (pre-test: 8.1; post-test: 7.7; *p* < 0.001), whereas in the control group, hbA1c increased at the end of the intervention period (pre-test: 8.1; post-test: 8.4, *p* < 0.001); there was a statistically significant difference in change over intervention period between the groups (*p* < 0.001, effect size 2.2).	**Y**
Farinha et al., 2022 [52]	Portugal	7	M, F	102 (50 lost to follow-up)	≥ 65	Aquatic exercise Interventions of 45 min twice/week: continuous aerobic (AerG), interval aerobic exercise (IntG), and combined aerobic and muscle strength (ComG). Control: age-matched, no systematic exercise.	Fasting glucose	In both the continuous and interval aquatic exercise groups, significant differences between pre- and post-test fasting glucose (AerG: -5.3%, *p* = 0.006; IntG: -5.3%, *p* = 0.041). No significant difference was found for the control group or the combined aerobic and muscle strength group.	**S**
Randolph et al., 2020 [89]	USA	5.5	M, F	42 (13 M, 29 F)	65–85	i) Progressive aerobic exercise training intervention consisting of 10–15 min of warmup and 45 min of treadmill walking, followed by a five-minute cooldown period, three times per week vs. ii. essential amino acid supplement vs. iii. supplement + exercise vs. iv. control	Fasting glucose, fasting insulin, Insulin sensitivity (Matsuda index)	Exercise training exerted a positive effect on insulin sensitivity (Matsuda index) (*p* = 0.0121) post-intervention; insulin sensitivity in the exercise + supplement group did not change. Fasting insulin (*p* = 0.0116) and fasting glucose (*p* = 0.0493) decreased after the exercise intervention; but no significant within-group changes in fasting plasma glucose were observed.	**S**
Letnes et al., 2022 [72] Generation 100 Randomized Trial	Norway	60 (5 years)	M, F	1567	70–77	5-year two weekly sessions of (i) High-Intensity aerobic interval training (walking/running, cross-country skiing and aerobics), or (ii) moderate-intensity continuous training (together, these exercise groups formed the “ExComb” group) vs. “control” (advised to follow national PA recommendations)	HbA1c, fasting glucose	No significant between-group differences. (Note, control group was given advice to follow national PA guidelines, and performed high levels of PA.)	**N**
OBSERVATIONAL STUDIES WITH PHYSICAL ACTIVITY EXPOSURE									
Nilsson et al., 2017 [76]	Sweden	Cross-sectional	F	120	65–70	Accelerometer (ActiGraph)	Fasting glucose	No relations were observed between plasma glucose and time in LPA or MVPA.	**N**
Kim & Kim, 2018 [67] Korean NHANES 2010–2012	South Korea	Cross-sectional	M, F	3554	≥ 65	Regular walking, determined by standardised questionnaire as > 30 min per day, > 5 days per week	Fasting glucose	In the “regular walking” group, fasting glucose levels were statistically significantly lower in comparison to the non-regular walking groups (101.41 ± 0.50 vs. 102.78 ± 0.41, *p* = 0.038). The high-BMI non-walked group had a significantly higher fasting glucose compared to the high-BMI regular walking group, the low-BMI non-walking group, and the low-BMI regular walking group.	**Y**
Cho et al., 2021 [64]	South Korea	Cross-sectional	M, F	358	≥ 70	Shortened, Korean version of IPAQ; MVPA	Fasting glucose, fasting glucose ≥100 mg	No significant association between fasting glucose and engaging in at least 150 min MVPA per week.	**N**
Zeng et al., 2020 [90]	China	Cross-sectional	M, F	2075	≥ 65	Self-report questionnaire; MPA, LPA, VPA.	T2DM: self-reported, fasting glucose ≥ 126 mg/dL or HbA1c ≥ 6.5%.	In older participants, conducting VPA 3–5 days/week (but no other frequency) and LPA of ≥ 240 min/day (but no other duration) was associated with a lower odds of T2DM (OR 0.35, 95% CI 0.16–0.77; OR 0.33, 95% CI 0.15–0.69, respectively) ); in addition, a significant association was found between LPA volume of > 1260 min/week and T2D (OR 0.32, 95% CI 0.15–0.69). No significant associations were found for any other frequency, type, or duration of PA and T2D.	**S**
Coelho-Júnior et al., 2023 [87]	Italy	Cross-sectional	M, F	3219 (1441 M, 1778 F); with BMI > 18.5	≥ 65	Self-report survey, physically inactive: did not practice ≥ 60 min at least twice per week during previous year	Capillary blood glucose	Adherence to aerobic training was inversely associated with glucose (-3.2, 95% CI -6.0, -0.4), but not after adjustment for age, sex, BMI, and other relevant covariates.	**N**
LaMonte et al., 2017 [57] OPACH	USA	Cross-sectional	F	2484	≥ 80	Accelerometer (ActiGraph); low-light PA, high-light PA, MVPA	Fasting glucose	Among women > 80 years, a significant inverse association was found with glucose levels ≥ 100 and MVPA (OR 0.88, 95% CI 0.79–0.98), but not low-light PA, high-light PA.	**S**
Länsitie et al., 2021 [88] Oulu45 cohort	Finland	Cross-sectional	M, F	702 (293 M, 409 F)	67–70	Accelerometer (Polar Active) Survey for self-reported lifetime PA	By WC tertile: Fasting glucose, Oral glucose tolerance test, insulin, insulin sensitivity (Matsuda index), HbA1c	In the lowest WC tertile: MVPA was negatively associated with 30 min and 120 min serum insulin levels (*p* = 0.040 and *p* = 0.001, respectively) and positively associated with the Matsuda index (*p* = 0.017); LPA was negatively associated with 120-minute insulin levels. In the Highest WC tertile: LPA was negatively associated with 0 min insulin levels (*p* = 0.009) and HOMA-IR (*p* = 0.026), and HOMA-β (*p* = 0.013) values.	**Y**
Umegaki et al., 2018 [82] TOPICS trial	Japan	Cross-sectional	M, F	388 (198 M, 190 F)	65–85	Accelerometer (Lifecorder)	Fasting glucose, HbA1c, insulin, insulin resistance (HOMA-IR)	LPA was negatively associated with HOMA-IR (-0.240, *p* < 0.001). HOMA-IR was significantly associated with LPA after adjustment for other significant cardiometabolic factors and MVPA (-0.138, *p* = 0.009). HOMA-IR was not significantly associated with MVPA after adjusting for cardiometabolic factors and LPA, but was significantly associated with total daily steps (-0.148, *p* = 0.008)	**S**
Rajabi et al., 2021 [77]	Iran	Cross sectional	M, F	220 (117 M, 103 F)	≥ 65	Questionnaire (IPAQ); participants classified into low (< 3.0 METs), moderate (≥ 3.0 METs) or high PA (≥ 6.0 METs) categories	Fasting glucose	Fasting glucose was significantly lower in high PA vs. low PA group within both males and females (*p* ≤ 0.05).	**Y**
Yu et al., 2019 [86]	China	Cross-sectional	M, F	1744 (885 M, 859 F)	≥ 65	Validated survey of professional and leisure-time physical activity; categorised into Low PA, MPA, and high PA.	Fasting glucose, diabetes: fasting glucose ≥7mmol/L (126 mg/dL) and/or being treated for T2DM	The MPA group had a statistically significantly lower odds of T2DM, compared to the low PA group (12.3% vs. 17.5%, OR 0.70, 95% CI 0.52–0.94, *p* < 0.05). the prevalence of T2DM was also non-significantly lower in the high PA group (9%, OR 0.54, 95% CI 0.26–1.11, *p* > 0.05).	**Y**
Kujala et al., 2019 [55]	Finland	Cross-sectional	M, F	779 (378 M, 401 F; included 276 twin pairs)	71–75	Accelerometer (Hookie); MVPA, daily steps Self-reported disease restricting mobility	Self-reported questionnaire of diseases, including T2DM	MVPA was lower in those with T2DM compared to without (25 [IQR 15–35] vs. 36 [IQR 34–38], *p* < 0.001), after adjusting for age and sex. Similarly, daily steps were statistically significantly lower in those with T2DM. T2DM was also significantly higher among participants reporting a disease restricting mobility (21.2% vs. 11.8%, *p* = 0.001)	**Y**
McGregor et al., 2018 [73] Canadian Health Measure Survey	Canada	Cross-sectional	M, F	1454	65–79	Accelerometer (Actical)	Fasting glucose, insulin	A statistically significant inverse association was found between MVPA and fasting glucose ([log] -0.021, *p* = 0.019) and insulin level ([log] -0.116, *p* < 0.001). No statistically significant association was found for LPA with glucose or insulin level.	**S**

*N* sample size, *Y* yes, *S* some, *N* no, *M* male, *F* female, *HbA1c* Haemoglobin A1c, *T2DM* Type 2 diabetes, *HOMA-IR* Homeostatic Model Assessment for Insulin Resistance, *WC* waist circumference, *IPAQ* International Physical Activity Questionnaire, *GPAQ* Global Physical Activity Questionnaire, *PA* physical activity, *LPA* light physical activity, *MPA* moderate physical activity, *VPA* vigorous physical activity, *MVPA* moderate to vigorous physical activity, *MET* metabolic equivalent of task, *UK* United Kingdom, *USA* United States of America, *OR* odds ratio, *95% CI* 95% confidence interval, *SD* standard deviation

**Table 6:** Summary of studies reporting on dyslipidaemia

Author/ Reference	Country/ Territory	Follow-up (months)	Sex	*N* (additional population description)	Age (years)	Description of intervention/exposure measure	Outcome measured	Main findings	Association PA & outcome
PHYSICAL ACTIVITY INTERVENTION VS CONTROL									
Farinha et al., 2022 [52]	Portugal	7	M, F	102 (50 lost to follow-up)	≥ 65	Aquatic exercise Interventions of 45 min twice/week continuous aerobic (AerG), interval aerobic exercise (IntG), and combined aerobic and muscle strength (ComG). Control: age matched, no systematic exercise.	Total cholesterol, HDL-c, LDL-c, and triglycerides from fasting blood samples	No significant changes found in total cholesterol, HDL-c, LDL-c, and triglycerides following the intervention (*p* > 0.05).	**N**
Park et al., 2017a [91]	South Korea	1.7	F	21	≥ 70	15-session, twice-weekly gardening intervention developed as a low-to moderate intensity intervention.	Total cholesterol, HDL-c, LDL-c	Participants in the intervention group had significantly increased HDL-c (33.1 vs. 40,7, *p* = 0.01) and total cholesterol (136.5 vs. 164.0, *p* = 0.03) after the intervention, with no significant difference in the control group. No significant difference was found between pre- and post-test in LDL-c.	**S**
Letnes et al., 2022 [72] Generation 100 Randomized Trial	Norway	60 (5 years)	M, F	1567	70–77	5-year two weekly sessions of (i) High-Intensity aerobic interval training (walking/running, cross-country skiing and aerobics), or (ii) moderate-intensity continuous training (together, these exercise groups formed the “ExComb” group) vs. “control” (advised to follow national PA recommendations)	Total cholesterol, HDL-c, LDL-c, triglycerides from serum samples	No significant differences for HDL-c, LDL-c, triglycerides, or total cholesterol. (Note, control group was advised about national PA guidelines and performed a high level of PA; significance was assumed at *p* < 0.01).	**N**
OBSERVATIONAL STUDIES WITH PHYSICAL ACTIVITY EXPOSURE									
Coelho-Júnior et al., 2023 [87]	Italy	Cross-sectional	M, F	3219 (1441 M, 1778 F); with BMI > 18.5	≥ 65	Self-report survey, physically inactive: did not practice ≥ 60 min at least twice per week during the previous year	Total cholesterol from fasting capillary blood sample	No significant associations were found after adjusting for age, sex, and additional covariates.	**N**
Umegaki et al., 2018 [82]	Japan	Cross-sectional	M, F	388 (198 M, 190 F)	65–85	Accelerometer (Lifecorder); LPA, MVPA.	Total cholesterol, HDL-c, triglyceride from fasting serum sample	LPA was positively associated with HDL and negatively associated with triglycerides. Only triglycerides remained significantly associated with LPA after adjustment for other significant cardiometabolic factors and MVPA (-0.128, *p* = 0.007). Triglycerides were negatively and HDL was positively associated with total daily steps. Triglycerides were associated with MVPA, but not after adjustment for other cardiometabolic factors.	**S**
LaMonte et al., 2017 [57] OPACH	USA	Cross-sectional	F	2484	≥ 80	Accelerometer (ActiGraph); low-light PA, high-light PA, MVPA	Total cholesterol (> 240), HDL-c (< 50), triglycerides (≥ 150).	Among women > 80 years, a significant inverse association was found between high triglycerides as well as low HDL-c and low-light PA, high-light PA, and MVPA. No significant association was found for total cholesterol ≥ 240.	**S**
Rajabi et al., 2021 [77]	Iran	Cross sectional	M, F	220 (117 M, 103 F)	≥ 65	Questionnaire (IPAQ); participants classified into low (< 3.0 METs), moderate (≥ 3.0 METs) or high PA (≥ 6.0 METs) categories	Total cholesterol, LDL-c, HDL-c, triglyceride from fasting blood sample	For both males and females, triglyceride and LDL-C were significantly lower in the High PA vs. low PA group (*p* < 0.05); HDL-C was significantly higher in the high PA vs. low PA group.	**Y**
Yu et al., 2019 [86]	China	Cross-sectional	M, F	1744 (885 M, 859 F)	≥ 65	Validated survey of professional and leisure-time physical activity; categorised into light PA, moderate PA, and high PA.	Total cholesterol, LDL-c, HDL-c, triglycerides from fasting blood; dyslipidaemia: HDL-c < 1.02mmol/L; total cholesterol ≥ 6.21mmol/L; triglycerides ≥1.69mmol/L; LDL-C ≥ 3.38mmol/L.	The prevalence of dyslipidaemia was not significantly lower in the moderate or high PA groups compared to the low PA group (*p* > 0.05), in adjusted logistic regression.	**N**
McGregor et al., 2018 [73] Canadian Health Measure Survey	Canada	Cross-sectional	M, F	1454	65–79	Accelerometer (Actical)	LDL-c, HDL-c, triglycerides from fasting blood samples.	A statistically significant inverse association was found between triglycerides and time spent in MVPA ([log] coefficient − 0.038, *p* = 0.006), but no significant association was found for HDL-C or LDL-C and either MVPA or LPA.	**S**
Nilsson et al., 2017 [76]	Sweden	Cross-sectional	F	120	65–70	Accelerometer (ActiGraph)	HDL-c, Triglycerides	A significant association was found between HDL-c and MVPA, independent of sedentary time (β = 0.03 (95% CI 0.01–0.06), *p* < 0.05). No relations were observed between triglycerides and time in LPA and/or MVPA.	**S**
Sung et al., 2023 [81] Korean NHNES 2016–2018	Korea	Cross-sectional	M, F	893 (220 M, 673 F)	≥ 65	Questionnaire (GPAQ): inactive (0–249 MET min/week), somewhat active (250–499 MET min/week), active (500–999 MET min/week), and very active (> 1,000 MET min/week).	HDL-c, HDL-c < 40 mg/dL (male) or < 50 mg/dL (female), Triglycerides?	Compared to the inactive group, the “very active” group had a lower odds of having low HDL-C (OR 0.55, 95% CI 0.37–0.83, *p* < 0.01). No significant difference across PA groups was found for having high triglycerides.	**S**
Kong et al., 2021 [68]	Korea	Cross-sectional	M	77	≥ 65	Accelerometer (Actical), LPA, MPA, MVPA; categorised: low PA (MVPA < 60), moderate PA (MVPA 60–120), and high PA (MVPA > 120)	HDL-c, Triglycerides	HDL-C was significantly higher in the high PA group, but not the middle PA group, compared to the low PA group (59.4 [SD 14.2] vs. 43.8 [SD 7.8], *p* < 0.05). No significant differences were found for triglycerides.	**S**
Kim & Kim, 2018 [67] Korean NHANES 2010–2012	South Korea	Cross-sectional	M, F	3554 (1581 M, 1973 F)	≥ 65	Regular walking, determined by a standardised questionnaire as > 30 min per day, > 5 days per week. Analysis stratified across BMI category (high ≥23, low < 23 kg/m2)	triglycerides, HDL-c	Triglycerides were significantly lower (134.2 [SD 2.24] vs. 140.9 [SD 1.9]) and HDL-C was significantly higher (50.6 [SD 0.3] vs. 48.9 [SD 0.3]) in the regular walking vs. non-regular walking group.	**Y**
Cho et al., 2021 [64]	South Korea	Cross-sectional	M, F	358	≥ 70	Shortened, Korean version of IPAQ; MVPA^2^	HDL-c, HDL-c < 40 mg/dL in men, < 50 mg/dL in women; Triglycerides ≥ 150 mg/dL	For men and women ≥ 70 years, engaging in < 150 MVPA/week (compared to engaging in ≥ 150 min of MVPA/week), was significantly associated with odds of having high triglycerides; for HDL-c, this association was only significant for women.	**S**

*N* sample size, *Y* yes, *S* some, *N* no, *M* male, *F* female, *HDL-c* high-density lipoprotein cholesterol, *LDL-c* low-density lipoprotein cholesterol, *IPAQ* International Physical Activity Questionnaire, *GPAQ* Global Physical Activity Questionnaire, *PA* physical activity, *LPA* light physical activity, *MPA* moderate physical activity, *VPA* vigorous physical activity, *MVPA* moderate to vigorous physical activity, *MET* metabolic equivalent of task, *6MWT* 6-minute walking test, *UK* United Kingdom, *USA* United States of America, *OR* odds ratio, *HR* hazard ratio, *95% CI* 95% confidence interval, *SD* standard deviation

**Table 7:** Summary of studies reporting on blood pressure and hypertension

Author/ Reference	Country/ Territory	Follow-up (months)	Sex	*N* (additional population description)	Age (years)	Description of intervention/exposure measure	Outcome measured	Main findings	Association PA & outcome
PHYSICAL ACTIVITY INTERVENTION VS CONTROL									
Oliveira et al., 2016 [94]	Portugal	Single exercise session	M, F	18 (5 M, 13 F)	80–90	Single session of aerobic exercise consisting of 2 periods of 10 min of walking at an intensity of 40% to 60% of the heart rate reserve. 5 min of warm-up and cool-down were performed before and after the 2 periods of walking. Control: no physical exercise during this period	Post-exercise hypotension; Automated oscillometric	In the exercise group, the systolic blood pressure (SBP) at 20 (127.3 ± 20.9 mm Hg) and 40 min (123.7 ± 21.0 mmHg) postexercise was significantly lower in comparison with baseline (135.6 ± 20.6 mm Hg); SBP showed a significant interaction for group x time (*p* = 0.002); No differences were observed in diastolic blood pressure (DBP) in either group.	**S**
Wong et al., 2021 [95] CU-AEROBIC	China	6	M, F	107 (36 M, 71 F; 10 lost to follow-up)	≥ 65	Intervention: Aerobic dance training led by a physiotherapist, once per week for the first 2 months and twice per week thereafter; control group: stretching and health education	No information	Significant reduction from baseline in SBP in the intervention group compared to the control group (mean % change intervention − 5.9 [SD 17.7] vs. control 0.6 [SD 14.0], *p* = 0.038). There was no between-group difference in DBP.	**S**
Wong et al., 2019 [61]	China	5	F	100 (women with Stage 2 hypertension)	67–85	Intervention: instructor supervised swimming training program (combination of free style, breaststroke, and backstroke) for twice/week. Control group: visited the laboratory at the same frequency for non-PA recreational activities.	Automated oscillometric	There were significant group x time interactions (*P* < 0.05) for SBP and DBP. SBP (-9 SD 1 mm Hg) and DBP (-9 SD 1 mm Hg) significantly decreased (*p* < 0.05) in the SWM group, while no significant changes were observed in the control group.	**Y**
Cunha et al., 2018 [93]	Brazil	Single exercise session	F	24	65–80	Single continuous aquatic exercise session including dynamic warm-up period (5 min), an active exercise period (35 min), and a cooldown period (5 min) to total 45 min. Control: 45-minute session with no exercise	Post-exercise hypotension; Ambulatory blood pressure monitor	Post-exercise hypotension (PEH) was demonstrated for SBP and DBP in the intervention group, with major reductions seen 11 to 13 h following exercise. Compared to the control group, SBP was reduced after the intervention by 5.1 ± 1.0 mm Hg over 21 h (*p* < 0.001), by 5.7 ± 1.1 mm Hg during awake hours (*p* < 0.001), and by 4.5 ± 0.4 mm Hg during sleep hours (*p* = 0.004) (Fig. 3). In turn, DBP was lower following the intervention compared to control: 1.2 ± 0.3 mm Hg over 21 h (*p* = 0.043); 0.9 ± 0.6 mm Hg over awake hours (*p* = 0.101); and 1.4 ± 0.9 mm Hg over sleep hours (*p* = 0.039)	**Y**
Farinha et al., 2022 [52]	Portugal	7	M, F	102 (50 lost to follow-up)	≥ 65	Aquatic exercise Interventions of 45 min twice/week: continuous aerobic (AerG), interval aerobic exercise (IntG), and combined aerobic and muscle strength (ComG). Control: age-matched, no systematic exercise.	Automated oscillometric	No significant differences found between the groups before and after the intervention (*p* > 0.05). Within the continuous aquatic exercise group, a significant change was found in SBP (-4.5%, *p* = 0.013) and DBP (-5.2%, *p* = 0.002); in the interval exercise group, only DBP was significantly lower after the intervention (-3.9%, *p* = 0.046), whereases in the combined aerobic and muscle strength group, SBP (-3.6%, *p* = 0.018) and DBP (-5.1%, *p* = 0.004) were significantly lower, with no pre-post-test difference in the control group.	**S**
Ballin et al., 2019 [92]	Sweden	2.5	M, F	77	70	Compare effect of 10 week progressively vigorous interval training compared to a control group, in centrally obese elderly participants.	Automated oscillometric (Omron)	Participants in the intervention group significantly lowered SBP with − 7.3 ± 11.8 mmHg and DBP with 4.2 ± 6.8 mmHg from pre- to post-intervention (both *p* < 0.001). However, the change from baseline was not statistically significant between exercise and control groups.	**S**
Letnes et al., 2022 [72] Generation 100 Randomized Trial	Norway	60 (5 years)	M, F	1567	70–77	5-year two weekly sessions of (i) High-Intensity aerobic interval training (walking/running, cross-country skiing and aerobics), or (ii) moderate-intensity continuous training (together, these exercise groups formed the “ExComb” group) vs. “control” (advised to follow national PA recommendations)	Automated oscillometric (Philips Medizin Systeme), mean arterial pressure (MAP)	MAP was significantly improved in the high-intensity aerobic group at year 3 follow-up compared to the “control” group instructed to exercise according to national guidelines (-1.58 mmHg, 99% CI -3.1 to -0.06), but this was no longer significant at year 5. There were no significant group differences for SBP or DBP. (Note, significance was assumed at < 0.01)	**S**
Park et al., 2017a [91]	South Korea	1.7	F	21	≥ 70	15-session, twice-weekly gardening intervention developed as a low-to moderate intensity intervention.	Automated oscillometric, (Omron)	In pre- post-intervention comparison, participants in the intervention group had significantly lower SBP (148.0 [SD 13.2] vs. 135.5 [SD 11.5], *p* = 0.01) and DBP (84.6 [SD 10] vs. 77.7 [SD 8.4], *p* = 0.03) after the intervention, with no significant difference in the control group. No group x time interaction was tested.	**Y**
OBSERVATIONAL STUDIES WITH PHYSICAL ACTIVITY EXPOSURE									
Kong et al., 2021 [68]	Korea	Cross-sectional	M	77	≥ 65	Accelerometer (Actical), LPA, MPA, MVPA; categorised: low PA (MVPA < 60), moderate PA (MVPA 60–120), and high PA (MVPA > 120)	Automated oscillometric	SBP was lowest in the middle PA group (125.8 [SD 15.7] vs. 141.8 [SD 6.5] in low PA group, *p* < 0.05). SBP in the high PA group was non-significantly lower than in the low PA group. There was no significant between-group difference in DBP.	**S**
Kim & Kim, 2018 [67] Korean NHANES 2010–2012	South Korea	Cross-sectional	M, F	3554 (1581 M, 1973 F)	≥ 65	Regular walking, determined by standardised questionnaire as > 30 min per day, > 5 days per week. Analysis stratified across BMI category (high ≥23, low < 23 kg/m^2^)	No information	No significant difference in SBP or DBP between regular walking and non-regular walking groups.	**N**
Cho et al., 2021 [64]	South Korea	Cross-sectional	M, F	358	≥ 70	Shortened, Korean version of IPAQ; MVPA	Hypertension: ≥130 mmHg or DBP ≥ 85 mmHg	For women ≥ 70 years, but not men, engaging in < 150 MVPA (compared to engaging in ≥ 150 min of MVPA per week), was significantly associated with odds of having high BP (OR 1.84, 95% CI 1.27–2.67, *p* = 0.001)	**S**
Coelho-Júnior et al., 2023 [87]	Italy	Cross-sectional	M, F	3219 (1441 M, 1778 F); with BMI > 18.5	≥ 65	Self-report survey, physically inactive: did not practice ≥ 60 min at least twice per week during the previous year	Combined automated oscillometric, Omron and manual standard sphygmomanometer	In the univariate and fully adjusted model, aerobic training alone was not significantly associated with SBP or DBP. However, combined aerobic training and high protein intake were negatively associated with SBP after adjustment for covariates (-4.98, 95% CI -9.8, -0.08, *p* < 0.05)	**S**
LaMonte et al., 2017 [57] OPACH	USA	Cross-sectional	F	2484	≥ 80	Accelerometer (ActiGraph); low-light PA, high-light PA, MVPA	Manual standard sphygmomanometer; SBP ≥ 135.	Among women > 80 years, no significant inverse association was found between SBP ≥ 135 and low-light PA, high-light PA, or MVPA.	**N**
Umegaki et al., 2018 [82]	Japan	Cross-sectional	M, F	388 (198 M, 190 F)	65–85	Accelerometer (Lifecorder)	Standard sphygmomanometer	LPA, but not MVPA for total steps, was negatively associated with SBP (-0.136, *p* = 0.007) and DBP (-0.134, *p* = 0.008); SBP and DBP were not significantly associated with LPA or MVPA when adjusting for insulin resistance and other significant cardiometabolic factors.	**S**
Yu et al., 2019 [86]	China	Cross-sectional	M, F	1744 (885 M, 859 F)	≥ 65	Validated survey of professional and leisure-time physical activity; categorised into light PA, moderate PA, and high PA.	Automated oscillometric; hypertension: ≥140/90 mmHg.	The moderate PA group had a statistically significantly lower odds of hypertension, compared to the low PA group (70.1% vs. 79.4%, OR 0.62, 95% CI 0.49–0.79, *p* < 0.001). The prevalence of hypertension was also non-statistically significantly lower in the high PA group (69%, OR 0.64, 95% CI 0.39–1.03, *p* > 0.05).	**Y**
Kujala et al., 2019 [55]	Finland	Cross-sectional	M, F	779 (378 M, 401 F; included 276 twin pairs)	71–75	Accelerometer (Hookie); MVPA, daily steps Self-reported disease restricting mobility	Self-reported questionnaire of diseases, including hypertension	MVPA was lower in those with hypertension compared to those without (29 [IQR 26–33] vs. 39 [IQR 35–44], *p* < 0.001), after adjusting for age and sex. Similarly, daily steps were statistically significantly lower in those with hypertension than those without. Hypertension was also significantly higher among participants reporting a disease restricting mobility (60.3% vs. 44%, *p* = 0.001)	**Y**
McGregor et al., 2018 [73] Canadian Health Measure Survey	Canada	Cross-sectional	M, F	1454	65–79	Accelerometer (Actical)	Automated oscillometric	No significant associations were found between MVPA or LPA and blood pressure in older adults (*p* > 0.05)	**N**
Nilsson et al., 2017 [76]	Sweden	Cross-sectional	F	120	65–70	Accelerometer (ActiGraph)	Mercury sphygmomanometer	No significant associations were observed between SBP or DBP and time in LPA or MVPA.	**N**

*N* sample size, *Y* yes, *S* some, *N* no, *M* male, *F* female, *DBP* diastolic blood pressure, *SBP* systolic blood pressure, *IPAQ* International Physical Activity Questionnaire, *GPAQ* Global Physical Activity Questionnaire, *PA* physical activity, *LPA* light physical activity, *MPA* moderate physical activity, *VPA* vigorous physical activity, *MVPA* moderate to vigorous physical activity, *MET* metabolic equivalent of task, *UK* United Kingdom, *USA* United States of America, *OR* odds ratio, *HR* hazard ratio, *95% CI* 95% confidence interval, *SD* standard deviation

**Table 8:** Summary of studies reporting on MetS

Author/ Reference	Country/ Territory	Follow-up (months)	Sex	*N* (additional population description)	Age (years)	Description of intervention/exposure measure	Outcome measured	Main findings	Association PA & outcome
PHYSICAL ACTIVITY INTERVENTION VS CONTROL									
Letnes et al., 2022 [72] Generation 100 Randomized Trial	Norway	60 (5 years)	M, F	1567	70–77	5-year two weekly sessions of (i) High-Intensity aerobic interval training (walking/running, cross-country skiing and aerobics), or (ii) moderate-intensity continuous training (together, these exercise groups formed the “ExComb” group) vs. “control” (advised to follow national PA recommendations)	Continuous “cardiovascular disease risk score”, using MetS-components: sex-specific z-scores for waist circumference, MAP, inverse HDL-C, fasting glucose, triglycerides	High-intensity supervised aerobic training had a lower risk score compared to “control” group instructed to perform PA according to national guidelines, at year 3 of follow-up (-0.34, 99% CI -0.66, -0.02), but this was no longer significant at year 5 (-0.32, 99% CI -0.64-0.01). (Note, significance assumed at *p* < 0.001)	**S**
OBSERVATIONAL STUDIES WITH PHYSICAL ACTIVITY EXPOSURE									
Kim & Kim, 2018 [67] Korean NHANES 2010–2012	South Korea	Cross-sectional	M, F	3554 (1581 M, 1973 F)	≥ 65	Regular walking, determined by a standardised questionnaire as > 30 min per day, > 5 days per week. Analysis stratified across BMI category (high ≥ 23, low < 23 kg/m2)	MetS: 2001 NCEP ATP III	The prevalence of MetS was not significantly higher in the “non-regular” walking group compared to the regular walking group (35.8% vs. 30.7%, *p* = 0.468). However, non-regular walkers with a high BMI had a statistically significantly higher prevalence of MetS (47.6%), compared to those with high BMI who did regular walking (41%), low-BMI non-walking participants (17.5%) and low-BMI regular walking participants (15.3%), (OR compared to low-BMI walking group: 4.36, 95% CI 3.37–5.63).	**S**
Kong et al., 2021 [68]	South Korea	Cross-sectional	M	77	≥ 65	Accelerometer (Actical), LPA, MPA, MVPA; categorised: low PA (MVPA < 60), moderate PA (MVPA 60–120), and high PA (MVPA > 120)	MetS: 2001 NCEP ATP III	MetS was inversely associated with PA group; after adjusting for age, those in the high PA but not middle PA group had lower odds of having MetS compared to the low PA group (OR High PA = 0.09, 95% CI 0.01–0.82, *p* < 0.05).	**S**
Nilsson et al., 2017 [76]	Sweden	Cross-sectional	F	120	65–70	Accelerometer (ActiGraph)	Metabolic risk score (zMS) based on the assessed metabolic risk outcome variables (level of triglycerides, HDL-c; plasma glucose, waist circumference and mean blood pressure).	Time in MVPA was inversely associated with metabolic risk score independently of sedentary time [β = -0.06 (95% CI -0.11, -0.02) *p* < 0.05]. Isotemporal substitution analysis revealed a significant increase in clustered metabolic risk score (*p* < 0.05) when replacing a 10-min time block of MVPA with either LPA or time in sedentary activities. Associations were attenuated when WC was removed the metabolic risk score.	**Y**
Sung et al., 2023 [81] Korean NHANES 2016–2018	South Korea	Cross-sectional	M, F	893 (220 M, 673 F)	≥ 65	Questionnaire (GPAQ): inactive (0–249 MET min/week), somewhat active (250–499 MET min/week), active (500–999 MET min/week), and very active (> 1,000 MET min/week).	MetS: NCEP ATP III	Found a significant difference in mean recreational moderate PA between Mets (24.0 ± 9.2) and non-MetS participants (90.4 ± 18.0, *p* < 0.01) and in total PA between Mets (368.0 ± 43.8) and non-MetS participants (509.9 ± 44.8, *p* < 0.05) Compared to inactive participants, very active participants had a lower odds of having MetS (OR 0.51, 95% CI 0.33–0.81, *p* < 0.01.) This association was not significant for the active and “somewhat active” group of participants.	**S**
Gallardo-Alfaro et al., 2019 [96]	Spain	Cross-sectional	M, F	477 (246 M, 231 F)	≥ 65; 65–69 vs. ≥ 70	Validated Spanish version of the Minnesota Leisure Time Physical Activity Questionnaire; total activity, light-moderate activity, vigorous activity; active > 300 MET/day)	MetS according to the updated harmonized definition of the IDF and American Heart Association and NHLBI	Total activity was lower in MetS participants compared to non-MetS participants in those aged 65–69 (median 59.6 [IQR 73.3] vs. 81.2 [IQR 43.1], *p* = 0.037) and ≥ 70 (median 51.3 [IQR 50.8] vs. 82.8 [IQR 42.5], *p* < 0.001). In addition, among those ≥ 65, the percentage of those defined as active was higher among non-MetS participants compared to those with MetS (*p* < 0.05). No adjusted results presented.	**Y**
OBSERVATIONAL STUDIES WITH CARDIO-RESPIRATORY FITNESS EXPOSURE									
Sandbakk et al., 2016 [97]	Norway	Cross-sectional	M, F	874	70–77	Respiratory gas analysis during treadmill walking/running until voluntary exhaustion (VO2max)	MetS according to the updated harmonized definition of the IDF and American Heart Association and NHLBI: “Cardiovascular risk factor clustering” (CV-RF).	Decrease in VO2max corresponded to 57% higher odds in women (OR 1.57, 95% CI 1.34–1.84) and 67% higher odds in men (OR 1.67, 95% CI 1.44–1.95) of having CV-RF. High cardiorespiratory fitness attenuated the adverse effect of sedentary behaviour on CV-RF.	**Y**

*N* sample size, *Y* yes, *S* some, *N* no, *M* male, *F* female, *MetS* metabolic syndrome, *HDL-c* high-density lipoprotein-cholesterol, *MAP* mean arterial pressure, *NCEP ATP III* US National Cholesterol Education Programme Adult Treatment Panel III, *IDF* International Diabetes Federation, *NHLBI* National Heart, Lung, and Blood Institute, *NHANES* National Health and Nutrition Examination Survey, *IPAQ* International Physical Activity Questionnaire, *GPAQ* Global Physical Activity Questionnaire, *PA* physical activity, *LPA* light physical activity, *MPA* moderate physical activity, *VPA* vigorous physical activity, *MVPA* moderate to vigorous physical activity, *MET* metabolic equivalent of task, *UK* United Kingdom, *USA* United States of America, *OR* odds ratio, *95% CI* 95% confidence interval, *SD* standard deviation

**Table 9:** Summary of studies reporting on depression

Author/ Reference	Country/ Territory	Follow-up (months)	Sex	*N* (additional population description)	Age (years)	Description of intervention/exposure measure	Outcome measured	Main findings	Association PA & outcome
PHYSICAL ACTIVITY INTERVENTION VS CONTROL									
Kuo et al., 2018 [70]	Taiwan	2	M, F	36 (6 M, 30 F)	≥ 65	30-minute prescribed stepper walking program (PSWP), twice per week for 8 weeks.	GDS-15; ≥5 for depression	No statistically significant changes in GDS score were seen post-intervention, or between the group completing the intervention and the control group in terms.	**N**
Sun et al., 2021 [80]	Taiwan	3	M, F	122 (27 M, 95 F)	≥ 65	Music therapy with physical activity consisting of warm-up followed by main body movement (cardiorespiratory endurance using the 2-minute step test), ending in relaxation.	Chinese version of GDS-15 (Short Form, SF)	In the geriatric depression assessment, the intervention group showed a significant difference in the mean GDS score in the pre-test and post-test results (2.18 vs. 1.55 in the post-test, *p* < 0.001), no significant pre-post-test difference was found for the control group. Compared to the control group, ANCOVA analysis showed that the music therapy group performed significantly better in the GDS (F = 23, *p* < 0.001).	**Y**
Bouaziz et al., 2019 [99]	France	2.25	M, F	60 (16 M, 44 F)	≥ 70	30-minute Interval aerobic training programme with recovery bouts on a cycle ergometer, for 9.5 weeks; Control group: maintain their current sedentary lifestyle.	Goldberg anxiety and depression scale (GADS) depression sub-score ≥ 2	Compared to controls, the intervention group had a significantly lower percentage at risk of depression after 9.5 weeks of training (-42.8% vs. + 26.3%).	**Y**
Wong et al., 2021 [95] CU-AEROBIC	China	6; additional follow-up: 9	M, F	107 (36 M, 71 F; 10 lost to follow-up)	≥ 65	Aerobic dance training led by physiotherapist, once per week for first 2 months and twice per week thereafter; control group: stretching and health education	GDS-15	The intervention group reported significantly fewer depressive symptoms at week 12 and 24, compared to baseline, whereas the control group only improved at week 36. No significant between-group difference nor interaction effect between treatment groups for depression.	**S**
Ballin et al., 2019 [92]	Sweden	2.5	M, F	77	70	10-week progressively vigorous interval training compared to a control group, in centrally obese elderly participants.	Short Form Health Survey Questionnaire (SF-36), with a mental health subscale	The intervention group improved their mental health score by 6.0 points (95% CI 1.7–10.4, *p* = 0.007), compared to the control group.	**Y**
OBSERVATIONAL STUDIES WITH PHYSICAL ACTIVITY EXPOSURE									
Byeon et al., 2019 [100] Korea National Health and Nutrition Examination Survey	South Korea	Cross-sectional	M, F	256	≥ 65	Self-care questionnaire (days of walking, muscular strength performance, flexibility exercise, sitting, high-intensity physical activity, medium-intensity physical activity.)	PHQ-9 self-reporting screening tool; score ≥ 10 suggestive of depression	Aerobic physical activity, walking, and muscular strength exercise were not significantly associated with geriatric depression. Only flexibility exercises were significantly associated with depression scores (*p* < 0.05).	**S**
Dooley et al., 2022 [65] Atherosclerosis risk in communities (ARIC) study	USA	Cross-sectional	M, F	459 (185 M, 274 F)	71–92	Accelerometer (ActiGraph): low-light, high light, MVPA	CES-D	After adjusting for covariates, no significant differences were found in daily time spent in any of the PA types by depressive symptoms.	**N**
Bae et al., 2023 [98]	South Korea	Cross-sectional	M, F	283 (97 M, 186 F)	≥ 65	Accelerometer, MVPA	Korean version of the GDS-15 (short form)	MVPA was higher among nondepressed older adults for both genders as compared to those with depression. A significant nonlinear trend was detected for MVPA with depressive symptoms, starting from an average of 16 min per day. Individuals engaging in < 15 min MVPA/day were 2.28 times more likely to be depressed after adjusting for age, sex, drinking, and smoking (95% CI 1.00-2.86).	**Y**
Chen et al., 2023 [102] Study at National Taiwan University Hospital (Lai et al., 2022 [71])	Taiwan	Cross-sectional	M, F	180 (81 M, 99 F)	≥ 65	Accelerometer (ActiGraph), MVPA LPA	GDS-15	In adjusted regression, overall MVPA was significantly associated with lower total depressive symptom scores (B = -1.357, 95% CI -2.561, -0.153, *p* < 0.05). Overall LPA was not significantly associated with depressive symptoms, and neither was timing-specific (morning, afternoon, evening) MVPA or LPA. No significant associations were found in the logistic analysis for PA and depression (GDS-15 ≥ 5)	**S**
McDowell et al., 2018 [107] Irish Longitudinal Study on Ageing	Ireland	Cross-sectional and prospective: 24–48	M, F	1151	≥ 70; 70–79 vs. ≥ 80	Self-report (IPAQ), meeting PA guidelines	CES-D; ≥16 defined depression	Cross-sectional: in adjusted logistic regression, those aged 70–79 years (*n* = 896) meeting PA guidelines had significantly lower odds of depression (OR 0.35, 95% CI 0.18–0.68, *p* < 0.001, *p* < 0.01); this was not found to be significant in those aged > 80 years (*n* = 255). Walking tertiles were not significantly associated with depression in either age group. Prospective: meeting PA guidelines and walking tertile were not significantly associated with longitudinal odds of depression, after adjustment for covariates.	**S**
Cabanas-Sánchez et al., 2021 [101]	Spain	Cross-sectional and prospective: mean 27.7 months (SD 3.7)	M, F	Cross-sectional: 2489; Prospective: 1679	≥ 65	Accelerometer (ActiGraph), MVPA, LPA	GDS-10	Cross-sectional: Time spent in MVPA relative to other behaviours (sedentary behaviour, sleep, LPA) was beneficially associated with depression (γ = -0.397, *p* < 0.001). Replacing 30 min of sleep, sedentary behaviour, or LPA with MVPA was beneficially related to lower depression. Prospective: time spent in MVPA relative to other behaviours was not significantly associated with depression.	**S**
Marques et al., 2020 [106] Survey of Health, Ageing and Retirement in Europe	14 European countries	Cross-sectional and prospective: 48 (4 years)	M, F	- (Approx. 16000)	≥ 65; 65–79 vs. ≥ 80	Self-report of PA activities, MPA, VPA	EURO-D 12-item scale, depression score ≥ 4	For moderate and vigorous PA for both men and women aged 65–79 or ≥ 80 years, being active at least 1 time/week in the present was consistently associated with significantly lower odds of having depressive symptoms regardless of whether PA was performed at least 1/week at baseline or not (4 years prior).	**S**
Yasunaga et al., 2018 [111] Matsudo cohort	Japan	Cross-sectional	M, F	276 (171 M, 105 M)	65–85	Accelerometer, LPA, MVPA	Japanese version GDS-15	No significant associations were found for MVPA. LPA was significantly and favourably associated with the GDS-15 score (β = -0.138, *p* < 0.05). In a model adjusting for all activities simultaneously (LPA, MVPA, and sedentary behaviour) to determine the effect of increasing one activity type while keeping the others constant, no significant associations were found. Lastly, however, in an isometric substitution model, a 30-min unit of SB replaced with LPA was significantly and negatively associated with the GDS-15 score (β = -0.131, *p* < 0.05).	**S**
Lima et al., 2024 [105] ATHLOS cohort: HRS: Health and Retirement Study; SHARE: Survey of Health Ageing and Retirement in Europe; KLOSA: Korean Longitudinal Study	USA, multiple European countries, Korea	2004–2013; 24–108 (mean follow-up time not provided)	M, F	- (total 56818 for LPA and 62656 for VPA; *n* ≥ 70 years not provided)	≥ 70	Survey of self-reported light-to-moderate and VPA during the last week	CES-D and EURO-D	For those aged ≥ 70, light-to-moderate (once a week vs. never: incidence rate ratio [IRR] 0.561, 95% CI 0.527–0.598; ≥2 per week vs. never: IRR 0.409, 95% CI 0.387–0.432) or VPA (once per week vs. never: IRR 0.609, 95% CI 0.569–0.652; >=2 per week vs. never: IRR 0.527, 95% CI 0.492–0.564), in any weekly frequency, protected against incident depression.	**Y**
Park et al., 2017b [109]	England	Cross-sectional	M, F	85 (27 M, 58 F)	65–99	Accelerometer (ActiGraph) and PA diary; three groups from latent profile analysis: group 1: low physical function and PA with a highly sedentary lifestyle; group 2: moderate physical function and PA with a moderate sedentary lifestyle; group 3: high physical function and PA with an active lifestyle.	Hospital Anxiety and Depression Scale (HADS); depression subset	Mean depression statistically significantly decreased from group 1 (low PA) to group 3 (high PA) (Cohen’s d for group 1 vs. 3: -1.67, *p* < 0.001).	**Y**
Cacciatore et al., 2019 [33]	Italy	Cross-sectional analysis (within longitudinal mortality study)	M, F	300 (224 M, 76 F) participants with symptomatic systolic Heart Failure	≥ 65	Self-reported PA before the onset of heart failure, using PASE – categorised into tertiles	GDS-15	GDS was statistically significantly higher in the lowest compared to the middle and to the highest tertile of PASE score (7.3 [SD 2.5] vs. 5.8 [SD 3.2] vs. 3.9 [SD 2.1], *p* < 0.001). Multivariate linear regression analysis on the PASE score demonstrated an inverse relationship with GDS score (B = -0.402, *p* < 0.001)	**Y**
Tully et al., 2020 [110] SITLESS Study	Denmark, Spain, Germany, Northern Ireland	Cross-sectional	M, F	1360 (520 M, 840 F)	≥ 65	Accelerometer (ActiGraph)	The Hospital Anxiety and Depression Scale (HADS), Depression subset	Inconsistent findings for PA and depression. A higher level of LPA was associated with a lower (improved) depression score (B = -0.30, 95% CI -0.51, -0.43) in the single model analysis, but MVPA and sedentary behaviour associated with a higher depression score (MVPA 0.21, 95% CI 0.10–0.32). In the isotemporal substitution model, replacing 30 min of SB with LPA (0.55), replacing 30 min of LPA with SB (0.58), or replacing 30 min of MVPA with either SB (0.38) or LPA (0.28) was associated with increased depression.	**S**
Inada et al., 2021 [103] Nakanojo Study	Japan	60 (5 years)	M, F	192 (92 M, 99 F)	65–85	Accelerometer (Lifecorder); PA trajectories for MVPA (5 trajectories) and step count (6 trajectories)	The Hospital Anxiety and Depression Scale (HADS) Depression subset	Using ANCOVA, the depression scores at follow-up differed significantly among the MVPA trajectory groups (*p* = 0.04) after adjustment for baseline scores. However, there was no significant difference in depression scores at follow-up after additionally adjusting for age. Differences in diabetes score between MVPA trajectories may therefore be partly attributable to differences in age.	**S**
Jung et al., 2018 [104]	Japan	Cross-sectional	M, F	3054	70–95	Accelerometer (GT40-020); LPA, MVPA, step counts	GDS-15; score of ≥ 5 indicative of depressive symptoms	The difference in MVPA was not statistically significantly different between groups. Participants with depressive symptoms showed a significantly lower average step count and LPA duration than participants without depressive symptoms (5110.2 ± 2667.7 vs. 4746.5 ± 2476.7, *p* < 0.005; and 39.7 ± 17.0 vs. 36.1 ± 15.4, *p* < 0.001, respectively.) Only the difference in LPA remained significant in propensity-score adjusted models.	**S**
OBSERVATIONAL STUDIES WITH CARDIO-RESPIRATORY FITNESS EXPOSURE									
Monteiro-Junior et al., 2022 [108]	Brazil	Cross-sectional	M, F	76	≥ 65	Two-minute step test, scored according to the age of the participant	GDS (Brazilian version); ≥10 indicative of depressive symptoms	Poor CRF was associated with depressive symptoms (OR 1.06, 95% CI 1.02–1.10, *p* = 0.01), after adjustment for age, gender, and being in a long-term care facility.	**Y**

*N* sample size, *Y* yes, *S* some, *N* no, *M* male, *F* female, *GDS-15* Geriatric Depression Score-15, *CES-D* Center for Epidemiological Studies Depression Scale, *GADS* Goldberg Anxiety and Depression Scale, *BDI* Beck Depression Inventory, *PHQ-9* Patient Health Questionnaire-9, *PASE* Physical Activity Scale for the Elderly, *IPAQ* International Physical Activity Questionnaire, *GPAQ* Global Physical Activity Questionnaire, *PA* physical activity, *LPA* light physical activity, *MPA* moderate physical activity, *VPA* vigorous physical activity, *MVPA* moderate to vigorous physical activity, *MET* metabolic equivalent of task, *6MWT* 6-minute walking test, *UK* United Kingdom, *USA* United States of America, *OR* odds ratio, *HR* hazard ratio, *95% CI* 95% confidence interval, *SD* standard deviation

**Table 10:** Summary of studies reporting on frailty and falls

Author/ Reference	Country/ Territory	Follow-up (months)	Sex	*N* (additional population description)	Age (years)	Description of intervention/exposure measure	Outcome measured	Main findings	Association PA & outcome
PHYSICAL ACTIVITY INTERVENTION VS CONTROL									
Gawler et al., 2016 [115]	England	24	M, F	1256 (477 M, 779 F)	≥ 65	6-month intervention types: (1) home-based exercise programme (home intervention): 30-minute, three-weekly home exercises; vs. (2) community-centre based once-weekly, 1-hour sessions supplemented with twice-weekly home exercise sessions (community intervention); vs. 3. Control group: usual care	Fall risk using Falls Risk Assessment Tool (FRAT); Daily falls diary	No change in falls during the intervention; at the 12-months follow-up, there was a 26% reduction in falls in the community intervention vs. control group (IRR 0.74, 95% CI 0.55–0.99, *p* = 0.04). No significant difference was found for the home intervention group. At 24-months follow-up, the significant effect of community intervention vs. control had been lost, but the group who continued to achieve 150 min of MVPA/week up to 24 months post-intervention had lower fall risk than those who did not maintain MPVA (IRR 0.49, 95% CI 0.30–0.79, *p* = 0.004).	**S**
Sun et al., 2021 [80]	Taiwan	3	M, F	122 (27 M, 95 F)	≥ 65	Intervention: music therapy with physical activity consisting of warm-up followed by main body movement (cardiorespiratory endurance using the 2-minute step test), ending in relaxation.	Frailty using Japanese the Kihon Checklist; 0–3: robust condition; 4–7: pre-frail condition; and 8 or more: frail condition	Only the intervention group had a significant difference in the mean score of frailty between the pretest and post-test (8.08 vs. 5.84 in the post-test, *p* < 0.001). After adjusting for sex, the results of ANCOVA analysis showed that the intervention significantly improved the Kihon Checklist results (F = 63.509, *p* < 0.001)	**Y**
Huang et al., 2020 [118] TOPICS trial	Japan	12	M, F	415 (220 M, 415 F)	65–85	6-month group and home-based aerobic training (AT) vs. resistance training (RT) vs. combined training (AT + RT) vs. control (lectures about health promotion)	95-item Frailty Index	At 26 weeks, the AT group showed reduced frailty (mean change in score compared to control: -0.02, 95% CI -0.039, -0.001) following adjustment for covariates, but this difference was not maintained at 12 months. No significant differences were found for the RT and AT + RT groups.	**S**
OBSERVATIONAL STUDIES WITH PHYSICAL ACTIVITY EXPOSURE									
Higueras-Fresnillo et al., 2020 [117]	Spain	Cross-sectional	M, F	436	65–92	Activity monitor: Intelligent Device for Energy Expenditure and Activity (IDEEA); MVPA, walking time (average or brisk).	Frailty: Fried’s criteria (weight loss, exhaustion, low physical activity, slowness, weakness)	Walking at a brisk pace was inversely associated with frailty (β = -4.72, 95% CI -6.75, -2.71) when adjusting for age, sex, sociodemographic factors, BMI, substance use, chronic conditions, time in other activities within the same category. MVPA was significantly negatively associated with frailty score (β = -3.59, 95% CI -5.74, -1.44).	**Y**
Fung et al., 2023 [114]	USA	288 (12 years)	F	12,073	≥ 70	Self-reported PA via questions on common leisure time physical activities; quintiles	FRAIL scale (5 self-reported criteria: fatigue, low strength, reduced aerobic capacity, ≥ 5 illnesses, and ≥ 5% weight loss)	In women ≥ 70 years old, a quintile of PA/week was inversely associated with a risk of frailty. For example, HR for frailty risk in the highest total PA quintile vs. lowest quintile was (HR 0.51, 95% CI 0.47–0.54). This association was not as strong as in a lower age group (60–70 years). Similar findings were found for moderate PA, vigorous PA, and activity duration.	**Y**
Rogers et al., 2017 [130] ELSA	England	120 (10 years)	M, F	3863	≥ 65; 65–69 vs. 70–74 vs. 75–79 vs. ≥80	Self-report of leisure-time vigorous, moderate, and mild physical activities	Rockwood’s Frailty index, score ≤ 0.25: non-frail	Moderate PA at least once a week was associated with improved frailty progression of the cohorts aged: 65–69: (B = -0.034, 95% CI -0.047, -0.021, *p* < 0.0001); 70–74: (B = -0.028, 95% CI -0.044, -0.012, *p* = 0.001); 75–79: (B = -0.024, 95% CI -0.042, -0.007, *p* = 0.005); and 80+ (B = -0.039, 95% CI -0.054, -0.023, *p* < 0.0001).	**Y**
Toledo Study for Healthy Aging (TSHA):									
1. Mañas et al., 2018 [125]	Spain	Cross-sectional	M, F	519 (234 M, 285 F)	≥ 65	Accelerometer (ActiGraph)	Frailty Trait Scale (FTS): energy balance and nutrition, physical activity, nervous system, vascular system, weakness, endurance, and slowness	LPA and MVPA showed inverse relationships with frailty score (B LPA = -0.014, 95% CI -0.024, -0.04, *p* < 0.01; B MVPA = -0.094, 95% CI -0.136, -0.052, *p* < 0.01). In isotemporal substitution models, replacing 30 min per day of sedentary time with MVPA was associated with a decrease in the frailty score (B = -2.460, 95% CI -3.782, -1.139); no significant change in frailty score was found for replacing sedentary time with LPA.	**S**
2. Mañas et al., 2019 [123]		Cross-sectional		749 (346 M, 403 F)	≥ 65			MVPA was found to be a moderator in the relationship between sedentary time and frailty (sedentary behaviour x MVPA interaction: -0.42, 95% CI -0.83, -0.01, *p* = 0.042); Johnson-Neyman technique: 27 min/d of moderate-to-vigorous physical activity eliminated the increased risk of frailty associated with sedentary time.	**Y**
3. Rodríguez-Gómez et al., 2021 [129]		Cross-sectional		776 (360 M, 416 F)	≥ 65			Older people who spent more time in MVPA had lower frailty than people who spent lower or medium time in MVPA.	**Y**
4. Mañas et al., 2020 [124]		48		186 (88 M, 98 F) (494 at baseline)	67–90			Lower levels of MVPA at baseline predicted later frailty (-0.126, 95% CI -0.231, -0.021, *p* < 0.05), whereas initial frailty status predicted later sedentary behaviour but not MVPA (*p* = 0.18), suggesting that less time in MVPA unidirectionally impacts frailty.	**Y**
Corral-Pérez et al., 2023 [113]	Spain	Cross-sectional	M, F	179 (66 M, 113 F)	≥ 65	Accelerometer (GENEActiv)	Fried’s frailty criteria (weight loss/shrinking, exhaustion, low physical activity, slowness, weakness)	In frail vs. pre-frail groups, significant differences in total LPA (209.35 ± 86.15 vs. 174.12 ± 93.93, *p* = 0.026) and total MVPA (45.96 ± 40.23 vs. 25.65 ± 33.40, *p* = 0.006). In an adjusted model, significant association between frailty and total LPA (OR 0.995, 95% CI 0.991-1.000, *p* < 0.034), LPA bouts > 10 min (OR 0.986, 95% CI 0.976–0.996, *p* = 0.005); total MVPA (OR 0.983, 95% CI 0.972–0.996, *p* = 0.007), and MVPA in bouts of 10 min (OR 0.954, 95% CI 0.917–0.994, *p* = 0.023).	**Y**
Lefferts et al., 2021 [121] Physical Activity and Aging Study	USA	Cross-sectional and prospective (12)	M, F	427 cross-sectional 241 prospective; older adults with hypertension	≥ 65	Accelerometer-based pedometer (Omron), categorised into tertiles of total steps per day, and none/low/high aerobic (≥ 60steps/min) steps	Fried’s frailty criteria (weight loss/shrinking, exhaustion, low physical activity, slowness, weakness)	Cross-sectional: participants with frailty had lower odds of having high total steps (vs. low total steps) and high aerobic steps (vs. no aerobic steps) (OR 0.62, 95% CI 0.5–0.78; and OR 0.48, 95% CI 0.29–0.79, respectively, *p* < 0.001). Prospective: total steps per day were not significantly associated with incident frailty, but aerobic steps (≥ 1 aerobic step per day), was associated with a 73% lower risk of developing frailty compared to those performing no aerobic steps (*p* = 0.04)	**S**
Yuki et al., 2019 [133] National Institute for Longevity Sciences-Longitudinal Study of Aging (NILSLSA)	Japan	Mean 4.2 years	M, F	401 (223 M, 178 F)	65–82	Accelerometer; LPA, MVPA, step count	Frailty: shrinking, exhaustion, low physical activity, low grip strength, and slow gait speed; Robust: none; pre-frail: 1–2; frail: ≥3	In a fully adjusted model, participants taking < 5000 steps had greater odds of frailty (OR 1.85, 95% CI 1.10–3.11); and participants doing MVPA < 7.5 min/day had greater odds of frailty (OR 1.80, 95% CI 1.05–3.09). No significant association was found for LPA.	**S**
Haider et al., 2019 [116] Correlation between datasets: - European Health Interview Survey (EHIS) - Survey of Health, Ageing, and Retirement in Europe (SHARE)	11 European countries	Cross-sectional correlation	M, F	24,590 (PA based on country-level data)	≥ 65	SHARE European Health Interview Survey Physical Activity Questionnaire (EHISPAQ); physically active: ≥150 min/week	SHARE Frailty Index; (1) robust, and (2) prefrail or frail	In European countries with a higher proportion of physically active subjects in the general population, the proportion of prefrailty or frailty was lower in the elderly population (*r* = -0.745, *p* = 0.008).	**Y**
Zhang et al., 2020 [134] Urban Health Centres Europe (UHCE) Study	5 European countries	12	M, F	1735 (675 M, 1060 F)	≥ 70	SHARE European Health Interview Survey Physical Activity Questionnaire (EHISPAQ), MPA “regular frequency” (more than once a week), “low frequency” (once a week or less). Change in frequency between baseline and follow-up categorised as: (1) “continued regular frequency” (more than once a week), (2) “decreased frequency,” (3) “continued low frequency” (once a week or less), and (4) “increased frequency.”	Tilburg Frailty Indicator (TFI): three domains physical frailty, psychological frailty, and social frailty	Compared with participants who undertook MPA with regular frequency at baseline, participants with low exercise frequency were significantly more frail at follow-up after adjustment for frailty at baseline and covariates (B = 0.28, 95% CI 0.01–0.55, *p* < 0.05). Compared to participants who undertook MPA with a continued regular frequency, decreased MPA frequency (B = 1.31, 95% CI 0.99–1.63, *p* < 0.001) and continued low MPA frequency (B = 1.16, 95% CI 0.84–1.49, *p* < 0.001) were significantly associated with frailty at follow-up in adjusted models.	**Y**
Kojima et al., 2021 [120] Japan Gerontological Evaluation Study 2016	Japan	Cross-sectional	M, F	461 (237 M, 224 F)	69–80	Accelerometer	Self-report of falls in the preceding year: falls on a frozen road, falls on an unfrozen road, indoor falls, or no falls	In elderly participants living in cold, snowy regions in Japan, a higher MVPA and walking steps were associated with falls on frozen roads. For any fall location, greater MVPA was associated with falls (adjusted prevalence ratio for highest tertile MVPA vs. lowest tertile: 1.73, 95% CI 1.04–2.87).	**Y**
Zhao et al., 2023 [135] Japan Gerontological Evaluation Study 2014–2017	Japan	36 (3 years)	M, F	475 (218 M, 257 F)	70–74	Japanese version of short-term IPAQ; MVPA, walking	Frailty: Kaigo-Yobo Checklist; Home boundness, falling, and lower nutrition. Frail vs. non-frail	A daily walking time of 0.5–1 h/d was significantly inversely associated risk of frailty (OR 0.35, 95% CI 0.12–0.98). Longer daily walking durations were not significantly associated with decreased risk of frailty. No significant association was found between PA volume and risk of frailty. A U-shaped curve was found for the association of frailty score with both PA volume and daily walking time.	**S**
Yuan et al., 2022 [132] Shandong Rural Elderly Health Cohort (SREHC)	China	Cross-sectional	M, F	743 (298 M, 445 F)	≥ 75	Chinese version of Short-form IPAQ	Falls: in-person interviews. no falls vs. falls	In participants aged 75–100 years, no significant association was found between the level of PA or MVPA and the odds of falls.	**N**
Moradell et al., 2021 [126] EXERNET-Elder 3.0 project	Spain	Cross-sectional	M, F	70 (19 M, 51 F)	≥ 65	Accelerometer (GENEActiv), LPA, MVPA	Risk of falling using Vivifrail instrument	Those with a history of falls spent less time in LPA and MVPA. Older adults who had no fear of falling showed more time in LPA, MVPA, and sedentary behaviour and spent less time sleeping compared to the whole sample. Those at risk of falling and with previous falls showed higher levels of PA.	**S**
Scott et al., 2021 [131] Healthy Ageing Initiative (HAI)	Sweden	12	M, F	3334	70	Accelerometer (ActiGraph)	Self-reported falls in the past 12 months	LPA and MVPA bouts per week were not significantly associated with incident falls.	**N**
Chen et al., 2020 [112] Itoshima Frail Study (IFS)	Japan	Cross-sectional	M, F	819 (395 M, 424 F)	65–75	Accelerometer (Active style)	Japanese version of FRAIL-scale (Fatigue, resistance, ambulation, illness, weight loss)	PA variables including total MVPA time, MVPA in bouts of ≥ 10 min, and steps were significantly associated with a lower prevalence of frailty (*p* < 0.001) after adjustment for covariates and other PA types. No significant association was found for LPA and MVPA in bouts < 10 min and frailty.	**S**
Netz et al., 2021 [128]	Israel	Cross-sectional	M, F	122 (36 M, 86 F)	≥ 65; Women: 65–74 vs. ≥75	IPAQ	Frailty Index (FI): pathologies, mental status (MMSE), emotional status (depression, anxiety), communication (hearing, vision etc.), mobility, balance, daily activities, bladder and bowel functions, sleep, and social resources	No significant associations between IPAQ and frailty in men or women (in either age group for women)	**N**
Nagai et al., 2018 [127]	Japan	Cross-sectional	M, F	886	≥ 65	Accelerometer (ActiBand); MVPA, LPA	Frailty: Fried’s criteria (weight loss/shrinking, exhaustion, low physical activity, slowness, weakness) Categorised as non-frail (0 components), pre-frail (1–2 components) and frail (> 2)	LPA and MVPA were significantly lower in the frailty group (*p* < 0.001), although only the association with LPA remained significant after adjustment for age, gender, and education (OR 0.86, 95% CI 0.80–0.92) No significant difference in MVPA or LPA was noted between the non-frail and pre-frailty group (*p* = 0.410).	**S**
OBSERVATIONAL STUDIES WITH CARDIORESPIRATORY FITNESS EXPOSURE									
Kawashima et al., 2019 [119]	Japan	Cross-sectional	M, F	92 (54 M, 38 F); stable, hospitalised patients with worsening heart failure	≥ 65	Cardiopulmonary exercise testing using cycle ergometer	Frailty using The Kihon Checklist (KCL); 0–3: robust; 4–7: pre-frail; and 8 or more: frail	Peak VO2 and peak VO2/heart rate ratio were significantly lower in the frail group than in the non-frail group (*p* = 0.002, *p* < 0.001, respectively) Peak VO2 was significantly correlated with frailty score (*r* = -0.527, *p* < 0.001).	**Y**
Netz et al., 2021 [128]	Israel	Cross-sectional	M, F	122 (36 M, 86 F)	≥ 65; Women: 65–74 vs. ≥ 75	Predicted VO2	Frailty Index (FI): pathologies, mental status (MMSE), emotional status (depression, anxiety), communication (hearing, vision etc.), mobility, balance, daily activities, bladder and bowel functions, sleep, and social resources	Among women 65–75 years old, predicted VO2 was associated with frailty. No significant association with men or women > 75 years old. In regression analysis among women overall, VO2 was inversely associated with frailty (-0.003, 95% CI -0.005, -0.001).	**S**
Lin et al., 2022 [122]	Taiwan	Cross-sectional	M, F	2130 (547 M, 1583 F)	≥ 65; 65–69 vs. 70–74 vs. 75–79 vs. 80–84 vs. 85–89 vs. ≥ 90	8-ft up-and-go test, a 30-second chair stand test, a 2-minute step test, 6MWT	Reported history of falls	Physical fitness measured by the 8-ft up- and-go test, 6MWT, or 3- second chair stand test was higher in those who had no history of falls (*p* < 0.05 in one-way ANOVA). On all the tests, the participants who had fallen three times in the previous year exhibited lower physical fitness than those who had not fallen. In groups aged 80–84 years and ≥ 90 years, poor grip strength was associated with a higher frequency of falling; in the groups aged 70–74 years, 75–79 years, and ≥ 90 years, a shorter duration of single-leg standing was associated with a higher frequency of falling	**Y**

*N* sample size, *Y* yes, *S* some, *N* no, *M* male, *F* female, *FI* frailty index, *PASE* Physical Activity Scale for the Elderly, *IPAQ* International Physical Activity Questionnaire, *GPAQ* Global Physical Activity Questionnaire, *PA* physical activity, *LPA* light physical activity, *MPA* moderate physical activity, *VPA* vigorous physical activity, *MVPA* moderate to vigorous physical activity, *MET* metabolic equivalent of task, *6MWT* 6-minute walking test, *UK* United Kingdom, *USA* United States of America, *ANOVA* analysis of variance, *OR* odds ratio, *HR* hazard ratio, *95% CI* 95% confidence interval, *SD* standard deviation

### Global summary of findings

In the longitudinal studies, PA and CRF were consistently associated with reduced all-cause mortality. Most included studies found inverse associations between measures of PA and CVD risk (*n* = 14 of 16 studies) and dysglycaemia outcomes (*n* = 12 of 16 studies). Obesity/adiposity, blood pressure/hypertension, dyslipidaemia, MetS, and frailty/falls were not consistently associated with PA. While PA interventions and cross-sectional analyses generally indicated improved depression outcomes, longitudinal studies showed inconclusive results. Results are presented in detail per outcome below and in Tables 2, 3, 4, 5, 6, 7, 8, 9 and 10.

### All-cause mortality

#### Descriptive summary of included studies

Seventeen (17) studies investigated all-cause mortality as an outcome of interest [32–48], of which thirteen (13) reported on PA while four (4) reported on CRF. All-cause mortality data were gathered from death registries/death indexes and data agencies, physician reports, hospital files, and obituaries. Four (4) studies used accelerometery to measure PA [35, 37, 40, 41]. Table 2 provides an overview of the main findings per study. Sample sizes ranged from 300 to 137626 and median or overall follow-up times ranged from 1 year to 22 years. The two (2) studies with overall or median follow-up times of < 2 years both included participants with underlying conditions, such as atrial fibrillation and frailty. In terms of the overall RoB, the one (1) RCT study on all-cause mortality showed some concerns, specifically due to deviations from the intended intervention [48] (Fig. 3A). Of the sixteen (16) observational studies, eleven (11) were good quality, four (4) were fair quality, and one (1) was rated as poor quality, due to the lack of comparability between the cases and controls and how the outcome was assessed [33], according to NOS criteria.

#### Overall findings for PA

Overall, higher levels of PA (i.e. highest quartile, time spent in moderate- to high-intensity exercise, frequency of moderate PA per month) and increased time spent in LPA were significantly associated with reduced risk of all-cause mortality across all the longitudinal studies. Compared to more active groups (categorical exposure), the HR for mortality associated with less active groups ranged from 1.1 to 7.3 [33, 41]; meanwhile, for each additional 1000 steps/day, Jefferis et al. [35] found an HR for mortality of 0.8, while Länsitie et al. [41] found an HR of 0.9 for every 10-minute increase in LPA.

#### Findings by PA intensity

Both LPA and MVPA were found to be associated with a reduced risk of all-cause mortality, even after adjusting for other PA intensities [35]. However, Länsitie et al. [41], with 660 participants and objectively measured PA found that only LPA and not MVPA was associated with lower mortality risk. This discrepancy in findings for LPA vs. MVPA, according to the authors [41], may be due to the fact that: (1) out of the 660 participants, a small sample of only 29 participant deaths (4.4%) had occurred during the 6.2-year (average; maximum 7.7 years) follow-up; and (2) the MVPA guidelines (150 min per week) may be too challenging to achieve in the older adult population, compared to the LPA threshold (2.00 and 3.49 MET), resulting in higher levels of LPA and therefore less power required to ascertain an association [41].

The only intervention study, the Generation 100 Randomised Trial reported by Stensvold et al. [48] additionally provides insight into the impact of various exercise intensities in a healthy, older population, finding no significant mortality differences by intensity level. The study found no significant difference in 5-year mortality between intervention groups, which consisted of high intensity exercise training, moderate intensity exercise training (both under some supervision), and a “control” advised to perform at least 30 minutes of moderate level PA almost every day. The only significant difference was found in unadjusted comparisons between the high-intensity training and “control”. The “control” group reported high levels of moderate- to high-intensity PA levels which diluted differences between groups. The authors also reported likely selection bias towards healthier, more active volunteers. As such, the study does not provide a direct comparison between low-PA and high-PA groups, but does provide insight into (a lack of) differences by PA intensity in older adults.

#### Findings for CRF

Similar to findings for PA, higher levels of CRF were significantly associated with lower all-cause mortality risk [32, 38, 44, 47]. In the study by Phan et al. [44], with a sample of 14499 participants in the USA, participants greater than 80 years old with high fitness levels (assessed with treadmill test) had comparable or even lower risk of mortality compared to their younger counterparts with moderate or low fitness levels. Finally, among 1242 older adult participants, Lu et al. [42] found that those physically inactive participants who also had low CRF had the highest risk of all-cause mortality.

#### Findings by age subgroup

Lastly, three (3) included studies compared age-subgroups [38, 40, 44]; LaMonte et al. [40] found no notable difference in results between older or younger subgroups; similarly, Phan et al. [44] found that low fitness was associated with greater risk of mortality within each age group (60–70, 70–80, vs. 80–90 years). In fact, the 80–90 year-old group with the highest fitness had similar or lower mortality risk compared to younger individuals with moderate or low fitness [44]. On the other hand, Kokkinos et al. [38] found that, while the association between PA and mortality was significant in both groups, the effect size was lower in the older group (Supplementary Table 3).

### Cardiovascular disease (CVD) risk

#### Descriptive summary of included studies

Sixteen (16) studies have been included to examine the effect of PA and CRF on CVD or CVD risk (Table 3**) [**41, 45, 48–61]. In these studies, CVD events included myocardial infarction, stroke/TIA, heart failure, acute coronary syndrome, coronary heart disease, or peripheral or pulmonary embolism incident cases [45, 49, 54, 60]. Studies assessing CVD risk using CVD surrogate markers (such as pulse wave velocity [PWV], intima media thickness [IMT] and augmentation index [AIx]) as well as 10-year clinical risk scores were also included. In order to determine 10-year CVD risk scores, two (2) studies utilised the Framingham Cardiovascular Disease Risk calculation [41, 50], one (1) study used the Reynolds Risk Score (RRS) [57], while another study employed the CHA₂DS₂-VASc Score for Atrial Fibrillation Stroke Risk [45]. In terms of the overall RoB, the three (3) RCT studies on CVD showed some concerns. For all observational studies (cohort and cross-sectional), eight (8) were good quality, two (2) were fair quality, and three (3) were rated as poor quality, due to the manner in which the participants were selected [58, 59] and/or the lack of comparability between the cases and controls [53].

#### Overall findings for PA

Fourteen (14) included studies found at least some evidence that both MVPA and LPA were inversely associated with CVD risk, arterial stiffness, or CVD-related death, and that participants with CVD, chronic heart disease (CHD) or heart failure presented with a lower level of MVPA [45, 49, 51, 55–57, 59, 60], including in admitted and outpatient participants with existing atrial fibrillation [45]. For instance, in the large, longitudinal EPIC Norfolk study, even moderately inactive participants aged > 65 years were found to have a lower risk of incident CVD events, compared to completely inactive participants (adjusted HR 0.86, 95% CI 0.78–0.96) [56]. Target population may be important to consider; while two (2) of the identified intervention studies consisted of aquatic exercise interventions [52, 61], Wong et al. [61] found a significant difference in CVD risk among women with pre-existing stage 2 hypertension, while the study conducted by Farinha et al. [52] (which had a 32% drop out rate and only included those completing at least 50% of exercise sessions in analysis [per protocol analysis]) showed no significant difference between IMT after the intervention. Lastly, results suggest possible effect modification of the association by adiposity. In the study by Länsitie et al. [41], statistically significant associations with CVD risk score were lost after adjusting for WC. Similarly, Lind et al. [58] stratified CVD risk by both obesity and PA status, and found that PA did not fully correct the increased CVD risk associated with obesity.

#### Findings by PA intensity

Other included studies suggest that the type of PA may impact the association of interest. For example, Emerson & Gay [50] and Jefferis et al. [54] found that only MVPA, and not LPA was inversely associated with the risk of CVD (ß for MVPA: -0.04, *p* = 0.041, and HR for MVPA: 0.34, 95% CI 0.16–0.74, *p* < 0.05, respectively). The Generation 100 Randomised Trial (described under “All-cause mortality”) [48] also did not find a significant association in CVD events/death between groups, with the “control” group performing high levels of moderate- to high-intensity exercise; in fact, the "control" group performed comparatively greater levels of high intensity exercise than the moderate-intensity intervention group.

#### Findings for CRF

Only one (1) cross-sectional study, He et al. [53], reported on the association between CRF and CVD, finding that CRF was significantly inversely associated with coronary artery disease, but not after adjusting for skeletal muscle mass.

#### Findings by age subgroup

Three (3) studies explored the impact of PA by age subgroup. Although Emerson & Gay [50] found no significant difference in the effect of MVPA on CVD risk between those aged 65–74 and ≥ 75 years, two (2) other studies found CVD risk reduction by PA [49] or light PA alone [60] was limited to younger age groups (< 75 years or < 80 years) (Supplementary Table 3).

### Obesity and adiposity

#### Descriptive summary of included studies

Twenty-eight (28) studies investigated measures of adiposity or obesity, as summarised in Table 4 [57, 59, 61–86]. The most common outcome measure was BMI (and BMI-derived categories including overweight and obesity), followed by WC, FM, and BF%. For the randomised intervention studies on adiposity/obesity, five (5) studies were rated as having some concerns, one (1) study had some concerns/high risk due to concerns with randomisation, potential deviation from the intended intervention, and selection of the reported result [74]; while one (1) other study was low risk for the overall RoB. The seven (7) intervention studies evaluated widely varying PA interventions, including swimming, dance training, and walking-based interventions. For the twenty-one (21) observational studies (which were all cross-sectional), three (3) were good quality, nine (9) were fair quality, and nine (9) were rated as poor quality, due to the manner in which the participants were selected [59, 82, 86] and/or the lack of comparability between the cases and controls [64, 68, 77, 78, 81], and/or how the outcome was assessed [85].

#### Overall findings for PA

The results from both intervention and observational studies were inconsistent, with five (5) intervention studies, reporting walking, swimming, and aerobic interval training interventions, found to positively impact at least one adiposity measure compared to a "control" group. Fifteen (15) observational studies found an association between PA (including walking) and lower adiposity measures, particularly in studies using adiposity measures other than weight and BMI, such as WC, FM, and BF% [64, 67, 68, 76, 77, 82]. Moreover, in an isotemporal substitution study, Lai et al. [71] found that replacing 30 minutes of LPA with MVPA was associated with a lower WC (but not BMI or BF%).

In contrast, two (2) intervention studies [79, 80], including a music-based intervention with a low RoB rating [80], showed no statistically significant effect on BMI or FM. Similarly, several observational studies, including three (3) using objectively measured aerobic PA [63, 66, 78, 83, 85], did not find an association between PA and adiposity/obesity. The 5-year Generation 100 Randomized Trial (Letnes et al. [72]) found a sustained impact of supervised high-intensity aerobic training on BMI through 5 years of training and follow-up compared to a “control” group advised to follow national PA guidelines and performing relatively high levels of moderate-to-high intensity exercise; however, no significant group differences were found between this “control” and WC.

#### Findings by PA intensity

The impact of LPA vs. MVPA remains unclear; of the studies exploring LPA in addition to MVPA, two (2) found that LPA but not MVPA was inversely associated with obesity/adiposity [65, 82], and two (2) found that both were associated with the adiposity outcome [66, 69]; whereas one (1) showed an association with MVPA, but not LPA [73].

#### Findings by age subgroup

In one (1) study, by Lai et al. [71], the association between PA and adiposity outcomes was significant in those aged 65–74, but not ≥ 75 years, suggesting that the association may weaken with age [71] (Supplementary Table 3).

### Dysglycaemia and glucose metabolism

#### Descriptive summary of included studies

Sixteen (16) studies [52, 55, 57, 64, 67, 72, 73, 75–77, 82, 86–90] investigated dysglycaemia or measures of glucose metabolism as an outcome of interest (Table 5). In terms of the overall RoB, the four (4) RCT studies showed some concerns, specifically due to concerns in the “deviation from intended intervention” and “randomisation” domains. For the twelve (12) observational studies (only cross-sectional), two (2) were rated as good quality, five (5) were fair quality, and five (5) were rated as poor quality, due to the manner in which the participants were selected [82, 86] or the lack of comparability between the cases and controls [64, 77], or how the outcome was assessed [90]. Six (6) of the observational studies used objectively measured accelerometery data to quantify PA exposure, while the other six (6) relied on self-reported PA measures.

#### Overall findings for PA

Twelve (12) included studies, both intervention and observational, found some association between PA and glucose metabolism outcomes. Of the intervention studies, a walking-based intervention by Molina-Sotomayor et al. [75] found a significant between-group difference in HbA1c, comparing the walking and "control" group. Two (2) other interventions (an aquatic exercise intervention [52], and an aerobic exercise training intervention [89]) reported significant pre- to post-intervention differences), for HbA1c, fasting glucose, and insulin sensitivity (Matsuda index) after 5.5-7 months, respectively, but did not report between-group differences. When looking at T2DM as an outcome, two observational (2) studies [86, 90] showed a significant reduction in the odds of T2DM as a result of exercise.

#### Findings by PA intensity

However, the results by activity intensity were seemingly contradictory, regardless of whether this was determined by self-report or accelerometery. McGregor et al. [73] and LaMonte et al. [57] found that MPA or MVPA, but not LPA, had a significant negative association with fasting glucose or odds of diabetes. On the other hand, Zeng et al. [90] found an association between self-reported T2DM and LPA, but only at high volume (> 1260 min/week) and frequency; additionally, Umegaki et al. [82] found that LPA and total daily steps were negatively associated with insulin resistance (HOMA-IR), but MVPA was not when also adjusting for LPA. Two (2) observational studies (including one (1) rated high quality [87]) using categorical cut-offs (≥ 60 min of aerobic training at least twice per week, and at least 150 minutes of MVPA per week, respectively) did not find a significant association between PA and (fasting) glucose levels after adjusting for covariates [64, 87]. Lastly, Länsitie et al. [88] stratified their results by WC, and found that MVPA was beneficial for insulin sensitivity (Matsuda index) and serum insulin levels in those with lower WC, while for those in the highest WC tertile, LPA but not MVPA was statistically associated with lower fasting insulin levels, HOMA-IR, and HOMA-β [88]. As results may vary by type of activity and population characteristics (e.g. adiposity), the association between LPA and hyperglycaemia risk may therefore require further study.

### Dyslipidaemia

#### Descriptive summary of included studies

Fourteen (14) studies investigated markers of dyslipidaemia as an outcome of interest (Table 6) [52, 57, 64, 67, 68, 72, 73, 76, 77, 81, 82, 86, 87, 91]. Among the eleven (11) observational studies, five (5) used objectively measured accelerometery data to quantify PA exposure, while six (6) relied on self-reported PA measures. All of these studies had a cross-sectional study design. In terms of the overall RoB, the three (3) RCT studies showed some concerns. For all observational studies (only cross-sectional), one (1) was good quality, four (4) were fair quality, and six (6) were rated as poor quality, due to the way the participants were selected [82, 86] or the lack of comparability between the cases and controls [64, 68, 77, 81].

#### Overall findings for PA

The significance of the association between PA and dyslipidaemia for the included studies varied, with four (4) studies finding no association, and ten (10) finding varied associations, depending on the lipid measure. For example, among the observational studies, those using TC [57, 87] or categorical dyslipidaemia [86] as outcomes did not tend to find significant associations with aerobic PA, while results for studies exploring HDL-c, LDL-c, and TG individually were more varied. A number of studies found significantly higher HDL-c and lower LDL-c and TG in participants with higher levels of aerobic PA (high LPA and MVPA) [57, 64, 67, 68, 76, 77, 81, 82], including in participants who walked regularly (Kim & Kim) [67]. One (1) study found no significant association between time spent in MVPA and HDL-c/LDL-c, but did find an association with TG (*p* = 0.006) [73].

Results for the intervention studies were similarly inconsistent. Two (2) intervention studies [52, 72], including the Generation 100 Randomized Trial [72] (which compared to a “control” advised to follow PA guidelines and performing high levels of PA), found no significant differences between intervention groups. However, a gardening intervention by Park et al. [91] showed a significant increase in TC and HDL-c levels following the intervention, while no significant difference was found in the "control" group [91]. For this study; however, there was no significant difference observed between pre- and post-test LDL-c.

#### Findings by PA intensity

In terms of PA intensity, no clear benefit of either MVPA or LPA over the other was identified. A few of the observational studies identified associations only for MVPA, not LPA (e.g. McGregor et al. [73] for the association between MVPA and TG); on the other hand, Umegaki et al. [82] found that LPA was positively associated only with TG after adjustment for potential confounders and variables including MVPA level (-0.128, *p* = 0.07), but no significant associations with MVPA was found after full adjustment. Still another observational study found similar effect of low-light PA, high-light PA, and MVPA on TG and HDL-c [57].

### Blood pressure and hypertension

#### Descriptive summary of included studies

Eighteen (18) studies investigated blood pressure and/hypertension as an outcome of interest [52, 55, 57, 61, 64, 67, 68, 72, 73, 76, 82, 86, 87, 91–95]. The most common measure for blood pressure was automated oscillometric blood pressure machine (*n* = 10) followed by manual sphygmomanometer (*n* = 4). Table 7 provides an overview of the findings. There were eight (8) studies describing the effect of various PA interventions on blood pressure, including swimming, dance training, and walking-based interventions, with sample sizes ranging from *n* = 18 to 1567. In terms of the overall RoB, the eight (8) RCT studies showed some concerns; this was due to concerns with measurement of the outcome (e.g. self-reported hypertension), selection of reported results, possible deviations from the intended interventions, or concerns/missing information around the randomisation procedure (Fig. 3A). For all observational studies (only cross-sectional), one (1) was good quality, five (5) were fair quality, and four (4) were rated as poor quality, due to the way participants were selected [82, 86] or the lack of comparability between the cases and controls [64, 68].

#### Overall findings for PA

The evidence for the association between PA (interventions) and SBP/DBP or presence of hypertension was inconsistent. Among intervention studies, a beneficial difference in SBP or presence of hypertension between "control" vs. intervention groups for SBP or the presence of hypertension was found in two (2) studies [61, 95]. In addition, while the 5-year supervised exercise intervention study (the Generation 100 Randomized Trial [72]) found significantly improved mean arterial pressure in the combined medium- and high-intensity exercise group at 3-years follow-up compared to the “control” group (receiving advice to follow national PA recommendations and performing high levels of PA), this was no longer significant at year 5, and no difference was found for SBP or DBP. Among the other intervention studies, results showed a lack of significant between-group comparisons/only reported significant pre- vs. post-test differences (3) [52, 91, 92], or reported only on immediate post-intervention effect from a single exercise session, instead of longer-term impact (2) [93, 94]. The latter two (2) studies did show a direct post-intervention hypotensive effect, with Cunha et al. [93] identifying the greatest difference in SBP and DBP 11–13 h post-exercise.

Among the cross-sectional observational studies, results were similarly inconsistent. Six (6) observational studies found no significant association after adjusting for confounders [57, 67, 73, 76, 82, 87], while Kong et al. [68] found an association between level of PA and SBP, but not DBP. Two (2) observational studies did find a significant association [55, 86]; Yu et al. [86] found a significant association between moderate levels of PA and the odds of hypertension, whereas in a large study (*n* = 779) using self-reported hypertension, MVPA was significantly lower in those with hypertension compared to those without (29 [IQR 26–33] vs. 39 [IQR 35–44], *p* < 0.001) [55]. Lastly, Cho et al. [64] found an association between performing sufficient MVPA (150 minutes/week) and odds of having high BP, although the association was only found to be significant among women, not men.

#### Findings by PA intensity

Three (3) studies reporting on results by PA intensity found similar (negative) results for LPA and MVPA [57, 73, 76]. Although Umegaki et al. [82], found that LPA, but not MVPA or total steps, negatively associated with SBP and DBP, these associations were no longer significant after adjusting for relevant confounders. The impact of LPA vs. MVPA therefore remains unclear.

### Metabolic syndrome (MetS)

#### Descriptive summary of included studies

Seven (7) studies investigated the effect of PA and CRF on MetS (Table 8) [67, 68, 72, 76, 81, 96, 97]. This included one (1) intervention study and six (6) cross-sectional studies. Various criteria were used to define MetS, including the 2001 National Cholesterol Education Program-Adult Treatment Panel III (NCEP ATP-III), self-defined MetS scores, and the updated harmonised International Diabetes Federation (IDF), American Heart Association (AHA), and National Heart, Lung and Blood Institute (NHLBI) guidelines. In terms of the overall RoB, the one (1) RCT study showed some concerns. [72] For all observational studies (only cross-sectional), four (4) were fair quality, and two (2) were rated as poor quality, due to the lack of comparability between the cases and controls [68, 81].

#### Overall findings for PA

Four (4) studies presenting findings on the association between PA and MetS found inconsistent results, with the significance of the association seeming to depend on the degree of PA performed [67, 68, 72, 81].

#### Findings by PA intensity

In the Generation 100 Randomized Trial by Letnes et al. [72], comparing supervised PA exercise with a “control” (advised to follow national recommendations and performing high levels of PA), high-intensity training was only inversely associated with a score of MetS components at 3 years, (-0.34, 99% CI -0.66, -0.02); this association was not maintained after 5 years (-0.32, 99% CI -0.64, 0.01). No significant association was found for moderate-intensity training (although this may have been due to the high level of training found in the "control" group). Nevertheless, this seems to be in agreement with some of the cross-sectional observational results, with Kong et al. [68] and Sung et al. [81] finding that only the high PA or “very active” groups, and not the “middle active” or “somewhat active” groups, had a significantly lower odds of having MetS. Kim & Kim [67] found, among 3554 participants, that there was not a statistically significant difference in MetS prevalence between the regular and non-regular walking groups (30.7% vs. 35.8%, *p* = 0.468), possibly linked to the relatively low intensity of this activity (at least 30 minutes of walking per day). Among women, Nilsson et al. [76] also found that replacing 10-minute blocks of MVPA with LPA increased metabolic risk score, suggesting benefits of MVPA compared to LPA for MetS risk.

#### Findings for CRF

In the study exploring CRF, Sandbakk et al. [97] found that there is a 57% and a 67% increased odds of MetS in both women (OR 1.57; 95% CI 1.34–1.84) and men (OR 1.67; 95% CI 1.44–1.95), respectively, when there is a decrease in VO2max.

#### Findings by age subgroup

In a cross-sectional, unadjusted analysis of 477 participants, Gallardo-Alfaro et al. [96] found that total PA was lower in MetS participants compared to non-MetS participants, with no notable difference in results between those aged 65–69 years and ≥ 70 years old (Supplementary Table 3).

### Depression

#### Descriptive summary of included studies

Twenty (20) studies investigated the association between PA or CRF and depression (Table 9) [33, 65, 70, 80, 92, 95, 98–111]. The most common instruments used to measure depression were the Geriatric Depression Scale (GDS, *n* = 10), followed by the Center for Epidemiological Studies Depression Scale (CES-D) and the Hospital and Anxiety Depression Scale (*n* = 3 each). There were five (5) studies looking at the effect of PA interventions on depression, including a stepper walking program, combined PA and music therapy, and aerobic dance training with sample sizes from 36 to 122 participants and follow-up time of 2 to 9 months. For the randomised intervention studies on depression, four (4) studies had some concerns, while one (1) study was low risk for the overall RoB. For all observational studies (cohort and cross-sectional), eight (8) were good quality, seven (7) were fair quality, and three (3) were rated as poor quality, due to the lack of comparability between the cases and controls [108, 109], and how the outcome was assessed [33]. Of the fourteen (14) non-intervention studies, nine (9) used objectively measured accelerometery data to determine PA exposure, whereas five (5) used self-reported PA measures, and one (1) used a combination of both accelerometery and a PA diary.

#### Overall findings for PA

The results for an association between PA and depression were inconsistent, with four (4) prospective observational studies finding no significant association [101, 103, 106, 107]. Generally, participants in the PA intervention groups showed improved depression measures compared to "control" groups, except for two (2) interventions (a stepper-walking [70] and aerobic dance programme [95], which had 2- and 9-months follow-up). In line with this, a multi-country longitudinal cohort study by Lima et al. [105] among participants older than 70 years, found that both light- to moderate-PA and VPA protected against incident depression, regardless of weekly frequency (incidence rate ratio [IRR] 1/week vs. never light to moderate PA: 0.561, 95% CI 0.527–0.598; IRR 1/week vs. never VPA: 0.609, 95% CI 0.569–0.652). While the cross-sectional studies also supported this association [102, 104, 110, 111], the rest of the prospective observational studies did not find significant associations between baseline PA and prospective depression. In fact, the three (3) studies that had both a cross-sectional and prospective component, found significant cross-sectional associations but no prospective associations between measures of PA and depression [101, 106, 107].

#### Findings for CRF

In a cross-sectional study, Monteiro-Junior et al. [108] found that poor CRF, measured using a step-test, was positively associated with depressive symptoms (OR 1.06, 95% CI 1.02–1.10, *p* = 0.01) after adjusting for covariables such as age and sex.

#### Findings by age subgroup

Although Marques et al. [106] found that being active at least 1 time per week was associated with lower odds of depression across age groups (65–79 vs. 80), a cross-sectional study among 1151 participants [107] found a significant association in participants aged 70–79 years, but not among those ≥ 80 years.

### Frailty and/or falls

#### Descriptive summary of included studies

Twenty-five (25) studies explored the association between PA or CRF and frailty and/or falls (Table 10) [80, 112–135]. Frailty was determined using various frailty indices, which included criteria such as weight loss, exhaustion, slowness, weakness, and aerobic capacity, whereas falls were determined based on self-report. For the randomised intervention studies on frailty and/or falls, one (1) study had some concerns (in the domain referring to deviations from the intended interventions), while two (2) studies were low risk for the overall RoB. For all observational studies (cohort and cross-sectional), nine (9) were good quality, five (5) were fair quality, and nine (9) were rated as poor quality, due to the manner in which the participants were selected [117, 121, 126] and/or the lack of comparability between the cases and controls [113, 116, 119, 122, 128, 134].

#### Overall findings for PA

For frailty, walking, LPA, and MVPA tended to be significantly inversely associated with frailty, although only limited evidence for a sustained impact off exercise interventions was identified. A home-based exercise intervention was found to significantly reduce the frailty index but lost this impact at 12 months follow-up [118]. Sun et al. [80] found a significantly reduced frailty score in the group with the aerobic music intervention, but the follow-up period was only for 3 months. Although twelve (12) observational studies found some statistically significant inverse association between frailty and PA, a smaller (*n* = 122) cross-sectional study by Netz et al. [128] found no significant association between the Frailty Index and IPAQ responses. On the other hand, the longitudinal *Toledo Study for Healthy Ageing* found that increased MVPA alleviated the increased risk of frailty associated with sedentary time [123]. Moreover, in this study’s longitudinal analysis, lower MVPA level at baseline predicted later frailty (-0.126, 95% CI -0.231, -0.021, *p* < 0.05), but frailty at baseline did not predict future MVPA at 4 years follow-up (*p* = 0.18), indicating that less time in MVPA unidirectionally impacts frailty [124].

In contrast, the association between falls and PA was less evident; three (3) of the included studies found either no association [131, 132] or a positive association [115] between history of falling, risk of falling, or self-reported falls and PA. Moreover, a supervised, indoor aerobic fitness intervention reduced falls at 12 months [115], but the identified reduction in falls was lost after 24 months, except in those achieving 150 minutes of MVPA/week. Among observational studies, Yuan et al. [132] and Scott et al. [131] found no association between falls and total PA, LPA or MVPA. These results may be because of the more complicated causal relationship between falling (risk) and PA, with more time spent in PA potentially increasing the risk of falling (particularly in cold, snowy climates) [120], and history of falling as well as fear of falling also influencing participants’ level of PA [126]. Therefore, any beneficial impact of aerobic PA on the risk of falling may be difficult to determine, particularly in cross-sectional studies, due to the lack of a simple cause-and-effect relationship.

#### Findings for CRF

CRF was found to be inversely associated with frailty in cross-sectional studies reported on this relationship [119, 128], although only significant among younger (65–75 years) women for one of these studies [128]. Meanwhile, a history of falling was similarly found to be associated with lower levels of fitness [122].

#### Findings by age subgroup

Among studies reporting subgroup analyses by groups, the overall results were consistent with those within age subgroups, suggesting no notable difference by age, with the exception of the cross-sectional study by Netz et al. [128], who only found a significant association for CRF and frailty among women aged 65–75 years, compared to women ≥ 75 years and men of any age.

## Discussion

The aim of this systematic review was to evaluate recent evidence relating to the association between aerobic PA and CRF and all-cause mortality, NCDs (CVD, obesity and adiposity, dysglycaemia and T2DM, high blood pressure, dyslipidaemia, MetS, depression), and frailty and falls among older adults aged 65 years and older. Aerobic PA and CRF were consistently negatively associated with mortality risk, suggesting that aerobic PA should be encouraged among older adults to support longevity and healthy ageing. However, the existing evidence for the associations between PA/CRF and individual metabolic outcomes such as obesity, lipid profile, and hypertension is less conclusive. This is in line with findings from the previously published review of aerobic PA interventions by Bouaziz et al. [14], signalling unresolved research challenges (including significant heterogeneity in study design and PA measurement methods), and knowledge gaps for future exploration. These gaps include the absence of any studies from the Africa or the South East Asia region, despite most people with NCDs living in low- and middle-income countries [136].

For all-cause mortality, all the observational studies found an association between either LPA, MVPA and/or CRF and a reduced risk, despite there being heterogeneity in the measurement of PA and CRF (using self-report/survey data [33, 34, 36, 39, 42, 43, 45, 46], accelerometer data [35, 37, 40, 41], 6MWT/distance, 6-minute walk speed, peak oxygen uptake using the cycle ergometer, or the exercise tolerance test and treadmill speed and grade [32, 38, 44, 47]). Our results are similar to findings from the reviews and meta-analyses by Hupin et al. [137] and Fukushima et al. [138] in adults ≥ 60 years old. In fact, Fukushima et al. [138] found that even minimal doses (+/-7.5 MET-h/week) of PA levels showed a significant reduction in mortality risk by 19% to 22%. Similarly, our study identified evidence that both LPA and higher intensity PA were significantly associated with a reduction in all-cause mortality in adults ≥ 65 years. In one (1) case [41], an association was found for LPA but not MVPA, likely due to sample size constraints combined with lower likelihood of achieving MVPA thresholds in this age group, for whom LPA may be more achievable. Nevertheless, this aligns well with current PA guidelines for the general adult population. For example, for both the general adult population and older adults > 65 years, the WHO recommends replacement of sedentary time with any intensity of PA (including LPA) and regular weekly PA of at least 150–300 minutes at a moderate intensity level, 75–150 minutes at a vigorous intensity level, or a comparable combination of MPA and VPA [19]. This is extrapolated from moderate-certainty evidence of a curvilinear dose-response relationship between PA volume and all-cause and CVD mortality in the general adult population.

The current review of evidence in older adults consisted mostly of observational studies, with the single intervention study highlighting the challenges of identifying a suitable control group and of avoiding selection bias towards more active older adults in RCTs of this nature [48]. As such, older adults who participate in PA may differ from those who do not in terms of underlying health, early life factors, social determinants, and other risk factors [42, 44]. Nevertheless, our study findings support the association between PA and improved survival.

Similarly, the large majority of included studies revealed a significant, inverse association between either LPA, MVPA, or CRF (VO2max) and a variety of CVD-related outcomes, from arterial stiffness to CVD-related deaths. Such an association between PA/CRF and CVD risk is not unexpected considering that PA can help protect against the development of disease by increasing cardiorespiratory function, boosting immune function and response, and lowering other risk factors, including high-risk behaviours such as smoking [139]. In line with our results, a meta-analysis by Xie et al. [140] that investigated accelerometer-measured LPA and CVD risk, found that increased LPA produced a 10% reduction in CVD risk adults in ≥ 60 years, indicating that even low intensity levels of PA can produce benefits towards cardiovascular health. However, the RCTs exploring the impact of exercise programmes on CVD risk showed inconsistent results [48, 52, 61]. This may have been due to differences in the population group (existing type 2 hypertension) [52], limited follow-up period [61], high drop-out rates [61], or lack of PA difference between exposure and control groups [48]. Reverse causality (where existing health conditions may impact PA level, particularly in older adults who are more likely to have existing conditions) and confounding limit our ability to establish a causal relationship in terms of CVD risk in studies of older adults [38].

Two (2) included studies in our review [49, 60] only found a significant association between PA/LPA and CVD risk in “younger” participants aged 65–75 years or 65–80 years, respectively. Among the studies reporting on different age-subgroups, we additionally identified a trend of smaller effect sizes or lack of significance among older sub-groups for outcomes including BMI, MetS, and depression (Supplementary Table 3). This may indicate that the impact of PA/LPA on CVD is more limited in the very old (80 + years old), although this remains to be further studied, taking into account selection bias among an ageing population, sample size requirements, and PA type and duration.

Studies assessing the association between aerobic PA and obesity/adiposity reported inconsistent results. In a previous systematic review conducted by Kuhle et al. [141], aerobic and resistance-based exercise interventions revealed a statistically significant, but minor, reduction in WC and BMI for participants aged over 60 years from Brazil, Iceland, Japan, Great Britain, and the USA. The extent to which such findings can translate to (clinically) meaningful differences in adiposity among older adult populations in real-world, non-intervention settings is unclear. In fact, as concluded in the 2020 WHO guidelines for the general adult population [19], epidemiological evidence in the general adult population on the impact of objectively measured PA on weight change in adults is heterogenous and less well established than for other outcomes [142–144]. In our review, studies reporting on measures of adiposity other than BMI or body weight, such as WC, FM, and BF%, tended to show a stronger association with PA. In older adults, BMI is not able to (1) distinguish between the loss of skeletal muscle mass and increase in fat mass as one ages, (2) track changes in fat distribution/accumulation throughout the body, and (3) differentiate between normal (intended) weight loss vs. weight loss due to bone density loss or disease; all of which could potentially lead to an underdiagnosis or misdiagnosis of health risks, as reflected on by Khanna et al. [145]. As such, especially in older adults, BMI may not adequately reflect actual changes in adiposity and body composition, which could contribute to the heterogeneity in the reported findings.

Most studies on dysglycaemia reported a significant association between aerobic PA and measures of glucose metabolism, which is in line with the evidence base in the general adult population informing the 2020 WHO recommendations showing an inverse association between PA and risk of T2DM [19]. However, we found less consistent evidence for the impact of aerobic PA on other metabolic outcomes of interest, particularly blood pressure, lipid profiles, and the composite MetS classification. This seems to align with findings from the previous review by Bouaziz et al. [14], and may indicate that these metabolic risk factors, at an older age, are less sensitive to the benefits of PA, possibly due to the complex interaction of other environmental, behavioural, and comorbidity risk factors, including those determined earlier in life. For example, comorbidities and polypharmacy in older adults may weaken the associations between PA and these metabolic risk factors. Given our results for the CVD and mortality outcomes, this does not negate the clinical importance of PA, but indicates the complexity of measuring this impact using these metabolic risk outcomes. A better understanding of the role of PA in the reversibility of (life-course) risk in older adults, and of how PA interventions can be tailored to those more likely to respond, is needed to more comprehensively determine the potential benefit of PA interventions for metabolic health in this age group.

Other considerations include the use of self-reported PA (instead of objective measurement, which, for example, was not identified in any of the included studies on blood pressure), heterogeneity due to differences in intensity and duration of PA, and smaller within-sample differences with age as the available pool of potential participants converges towards those who are healthier and more active at baseline [146]. For hypertension, our results contrast with both the WHO 2020 guidelines for the general adult population [19], and a systematic review and meta-analysis by Kazeminia et al. [147], which reported a substantial reduction in blood pressure following exercise in adults with hypertension > 60 years. The intervention studies included in the review by Kazeminia et al. [147], in contrast to our review, included younger participants (60 + vs. 65+) with pre-existing hypertension, which may be a group particularly responsive to increased exercise; and included any type of exercise rather than solely aerobic exercise [147]. For lipid profiles, significant associations with PA were only observed when assessing individual lipid markers such as HDL-c, LDL-c, and TG [57, 64, 67, 68, 73, 76, 77, 81, 82], and not TC [57, 87] or categorical dyslipidaemia outcomes [86], possibly because more subtle changes in individual lipid markers (e.g. increase in HDL-c and decrease in LDL-c) are lost when using composite measures (e.g. TC) [148]. Inconsistencies around the impact of LPA vs. MVPA may also indicate that the optimal type or duration of aerobic PA may differ by individual or population group, requiring further exploration.

The final three outcomes evaluated in this review were frailty, depression, and falls. In line with the WHO 2020 PA recommendations for older adults [19], the *Toledo Heart Study’s* longitudinal analysis suggests that MVPA is unidirectionally associated with frailty, as frailty at baseline was not found to predict future MVPA [124], thereby challenging the impact of reverse causality for this outcome. From an implementation point of view, our results suggest that at-home-based exercise interventions may be less effective for reducing frailty when compared to community-based interventions [115, 118]. This may be due to a lack of adequate oversight (supervision) and support [115, 149].

Regarding depression, aerobic PA interventions were generally found to be beneficial, but one (1) RCT showed no significant effect [70]. This may have been due to a shorter follow-up period (2 months), a smaller sample size (*n* = 36), or lower intensity of PA (30 minutes of light-moderate- to moderate-intensity twice per week for 8 weeks). In fact, previous systematic review evidence in the general adult population suggests that exercise intensity (e.g. in terms of daily step count) may determine the impact of PA on depression [150]. Inconsistent results were found for the observational studies reviewed. Notably, three (3) studies that had both cross-sectional and prospective components found significant cross-sectional associations, but not prospective. This highlights the complex relationship between depression and PA, as reduced PA by itself is also a symptom of depression [151].

Similarly, there is likely a bidirectional relationship between aerobic PA and (risk of) falls, where individuals with a history of falling or functional limitations may be less inclined to participate in aerobic PA. In high-risk environments (such as participants who live alone, in winter climates, etc.), aerobic PA may also increase the risk of falls. Therefore, while the WHO 2020 guidelines on PA and sedentary behaviour provide high certainty evidence that PA combining balance, strength, gait, and functional training is beneficial for the risk of falls, the evidence for aerobic PA seems to be less well established [19, 152].

### Research gaps

This review highlights a significant geographical research gap, with no studies identified from the African or South East Asian regions, despite the majority of the global NCD burden occurring in low- and middle-income countries [136]. This lack of evidence limits the generalisability of current findings to these settings and emphasises the need for greater research investment, context-specific studies, and community-engaged approaches to better understand the role of aerobic PA and CRF in reducing NCD risk among older adults in these regions.

Our results also identified limited evidence on the effects of PA on CVD among the oldest-old (≥ 80 years). Notably, several studies reported significant associations only in “younger” older adults, with weaker or non-significant effects observed in older subgroups. This pattern may reflect the cumulative burden of life-course risk, reverse causality (whereby declining health limits PA participation), or differences in the type, intensity, feasibility and suitability of PA for the oldest-old. Further age-stratified research in this population is needed to clarify these relationships.

Further research is also needed to identify which population subgroups benefit most from PA, particularly individuals with multimorbidity, medication use, or co-existing infectious diseases, for whom evidence on the safety, feasibility, and effectiveness of tailored PA interventions remains limited.

### Strengths and limitations

A strength of this review is the inclusion of additional outcomes, above those that were included in a previous review [14], to broaden the body of evidence and perspective regarding the association with aerobic PA in the older adult population and across different settings. We also included both intervention and observational studies, to allow for a deeper understanding of the available literature. As a result, we noted a need for more longitudinal studies, as most of the included manuscripts were cross-sectional studies. This precludes establishing causality, particularly due to the possibility of reverse causality between the exposure and outcome variables. Among older adults, whose PA levels might be substantially affected by adverse health events, single timepoint measures of PA might not fully capture the complex associations between PA and health outcomes.

Our results should be interpreted in the context of several limitations. In general, the quality of the evidence (individual study findings per outcome) of the included RCTs are inconsistent, ranging from low to moderate, with only one study (1) demonstrating high quality evidence [118]. Similarly, the observational studies included in this review (rated using the NOS) ranged from poor (*n* = 25), fair (*n* = 27), to good (*n* = 39) quality. A large number of studies included self-reported data, which is prone to recall bias and may lead to over- or understating the actual PA status of participants, with self-reporting of LPA being particularly challenging [146, 153]. Although considered an accurate free-living method of objective PA measurement, accelerometer-derived data has its own limitations, such as being unable to distinguish between leisure-time vs. other PA, which may have contributed to differences in findings between studies using self-report vs. accelerometery [154, 155].

Another limitation of this review is that during the screening process, we did not specifically exclude studies that included participants with pre-existing conditions (co-morbidities), which could introduce variability into the study population and could pose a challenge when interpreting the results. However, this allowed us to gain a more inclusive insight into the contexts in which PA may be associated with NCDs. Due to the already broad scope of our review, we did not include studies on sarcopenia (loss of skeletal muscle mass), as it is not generally categorised as a core NCD. However, sarcopenia is an important risk factor for multi-morbidity and NCD morbidity in older adults, and its lack of inclusion in this review is a limitation. Muscle mass loss is accounted for indirectly in the frailty indices and scores.

By limiting the scope of the review to aerobic PA, we were also not able to evaluate the impact of other types of PA (such as strength and balance training) on outcomes of interest, such as falls (although this association was the subject of a 2020 systematic review) [152]. The heterogeneity among the included studies also precluded the use of meta-analyses for composite summary estimates of the impact of PA on the outcomes of interest, due to varying lengths of the interventions and follow-up times, large variations in the exposure or comparator groups, and a lack of consistency in terms of measuring the dose of PA (frequency, duration, and intensity). Lastly, the possibility of publication bias cannot be excluded, whereby studies reporting negative or null findings may be underrepresented, thereby potentially influencing the overall conclusions of the review.

### Conclusion

This review provided a comprehensive overview of the available evidence on the association between aerobic PA and CRF, all-cause mortality, and several NCDs. We found inconsistent evidence for obesity, dyslipidaemia, hypertension, Mets, and depression. However, despite the heterogeneity across studies, the evidence suggests that regular aerobic PA (at varying intensities) may be beneficial in terms of all-cause mortality, CVDs, frailty, and dysglycaemia outcomes. Given the findings for these outcomes, and in line with recommendations for the general adult population under the age of 65 years [19], public health initiatives should continue to promote the incorporation of regular aerobic PA into daily routines for older individuals. Importantly, given the inconsistent findings in the evidence for key metabolic outcomes, such as obesity, dyslipidaemia, hypertension, and MetS, future research is needed to address outstanding and understudied questions around risk reversibility and time to benefit for PA interventions, as well as identifying target groups who benefit more from PA and tailoring interventions to these groups. In addition, further high-quality longitudinal studies focusing on aerobic PA and CRF and its ideal frequency, duration, and intensity are required to improve the body of evidence and identify the most effective interventions for older adults.

## Electronic Supplementary Material

Below is the link to the electronic supplementary material.

Supplementary Material 1: Supplementary File 1: PRISMA 2020 Checklist. Supplementary Table 1: Search Strategy and articles retrieved from all databases searched. Supplementary Table 2: Data extraction tool. Supplementary Table 3: Synthesis of results by within-study comparison of age groups ≥65 years.

## Data Availability

Data not presented in the manuscript can be requested from the corresponding author upon reasonable request.

## Abbreviations

AHA: American Heart Association
AIx: Augmentation index
BDI: Beck Depression Inventory
BF%: Body fat percentage
BIA: Bioelectrical impedance analysis
BMI: Body mass index
BP: Blood pressure
BW: Body weight
CES-D: Center for Epidemiological Studies Depression Scale
CHD: Chronic heart disease
CIMT: Carotid intima-media thickness
CRF: Cardiorespiratory fitness
CVD: Cardiovascular disease
DBP: Diastolic blood pressure
DEXA: Dual-energy X-ray absorptiometry
EF: Ejection fraction
FI: Frailty index
FM: Fat mass
GADS: Goldberg Anxiety and Depression Scale
GDS: Geriatric Depression Scale
GPAQ: Global Physical Activity Questionnaire
HbA1c: Haemoglobin A1c
HC: Hip circumference
HDL-c: High-density lipoprotein cholesterol
HOMA-IR: Homeostatic Model Assessment for Insulin Resistance
HR: Hazard ratio
IDF: International Diabetes Federation
IMT: Intima media thickness
IPAQ: International Physical Activity Questionnaire
IRR: Incidence rate ratio
LDL-c: Low-density lipoprotein cholesterol
LPA: Light-intensity physical activity
LTCF: Long-term care facilities
LVMI: Left ventricle mass index
MeSH: Medical subject headings
MET: Metabolic equivalent of task
MetS: Metabolic syndrome
MPA: Moderate-intensity physical activity
MVPA: Moderate- and vigorous-intensity physical activity
NCD: Non-communicable disease
NCEP ATP-III: US National Cholesterol Education Programme Adult Treatment Panel-III
NHLBI: National Heart, Lung, and Blood Institute
NOS: Newcastle-Ottawa Scale
OR: Odds ratio
PA: Physical activity
PASE: Physical Activity Scale for the Elderly
PHQ-9: Patient Health Questionnaire-9
PWV: Pulse wave velocity
RCT: Randomised controlled trial
RoB: Risk of bias
RR: Risk ratio
RRS: Reynolds Risk Score
SBP: Systolic blood pressure
SD: Standard deviation
SMI: Skeletal muscle index
TC: Total cholesterol
TG: Triglycerides
TIA: Transient ischemic attack
T2DM: Type 2 diabetes mellitus
UK: United Kingdom
USA: United States of America
VPA: Vigorous-intensity physical activity
WC: Waist circumference
WHO: World Health Organization
WtHR: Weight to height ratio
6MWT: 6-minute walk test
95% CI: 95% confidence interval

## Declarations

### Additional information

This review was not prospectively registered. The protocol can be requested from the corresponding author upon reasonable request.
