# Supplementary material for: Aerobic physical activity, cardiorespiratory fitness, and non-communicable diseases risk in older adults: a systematic review
Source: BMC Geriatr. 2026 Apr 30;26:848. doi: 10.1186/s12877-026-07541-4 (PMC13277166; doi:10.1186/s12877-026-07541-4)
Supplement: Supplementary file 1 — Supplementary Material 1: Supplementary File 1: PRISMA 2020 Checklist. Supplementary Table 1: Search Strategy and articles retrieved from all databases searched. Supplementary Table 2: Data extraction tool. Supplementary Table 3: Synthesis of results by within-study comparison of age groups ≥65 years. [file 12877_2026_7541_MOESM1_ESM.docx]

**Aerobic physical activity, cardiorespiratory fitness, and non-communicable diseases risk in the elderly: a systematic review**

**Supplementary Material**

**Table of Contents**

| **Table Number** | **Description** | **Page Number** |
| --- | --- | --- |
| Supplementary File 1 | PRISMA 2020 Checklist | Page 2-4 |
| Supplementary Table 1 | Search Strategy and articles retrieved from all databases searched | Page 5-8 |
| Supplementary Table 2 | Data extraction tool | Page 9 |
| Supplementary Table 3 | Synthesis of results by within-study comparison of age groups ≥65  years | Pages 10-12 |
|  | | |


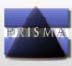
**Supplementary File 1: PRISMA 2020 Checklist**

| **Section and Topic** | **Item #** | **Checklist item** | **Location where item is reported** |
| --- | --- | --- | --- |
| **TITLE** | | |  |
| Title | 1 | Identify the report as a systematic review. | Title page |
| **ABSTRACT** | | |  |
| Abstract | 2 | See the PRISMA 2020 for Abstracts checklist. | Page 2 |
| **INTRODUCTION** | | |  |
| Rationale | 3 | Describe the rationale for the review in the context of existing knowledge. | Page 5 |
| Objectives | 4 | Provide an explicit statement of the objective(s) or question(s) the review addresses. | Pages 2, 5 |
| **METHODS** | | |  |
| Eligibility criteria | 5 | Specify the inclusion and exclusion criteria for the review and how studies were grouped for the syntheses. | Page 6 |
| Information sources | 6 | Specify all databases, registers, websites, organisations, reference lists and other sources searched or consulted to identify studies. Specify the date when each source was last searched or consulted. | Pages 6-7 |
| Search strategy | 7 | Present the full search strategies for all databases, registers and websites, including any filters and limits used. | Pages 6-7  Supplementary Table 1 |
| Selection process | 8 | Specify the methods used to decide whether a study met the inclusion criteria of the review, including how many reviewers screened each record and each report retrieved, whether they worked independently, and if applicable, details of automation tools used in the process. | Pages 6-7 |
| Data collection process | 9 | Specify the methods used to collect data from reports, including how many reviewers collected data from each report, whether they worked independently, any processes for obtaining or confirming data from study investigators, and if applicable, details of automation tools used in the process. | Page 7 |
| Data items | 10a | List and define all outcomes for which data were sought. Specify whether all results that were compatible with each outcome domain in each study were sought (e.g. for all measures, time points, analyses), and if not, the methods used to decide which results to collect. | Pages 6-7 |
|  | 10b | List and define all other variables for which data were sought (e.g. participant and intervention characteristics, funding sources). Describe any assumptions made about any missing or unclear information. | Pages 6-7 |
| Study risk of bias assessment | 11 | Specify the methods used to assess risk of bias in the included studies, including details of the tool(s) used, how many reviewers assessed each study and whether they worked independently, and if applicable, details of automation tools used in the process. | Page 7 |
| Effect measures | 12 | Specify for each outcome the effect measure(s) (e.g. risk ratio, mean difference) used in the synthesis or presentation of results. | Page 7 |
| Synthesis methods | 13a | Describe the processes used to decide which studies were eligible for each synthesis (e.g. tabulating the study intervention characteristics and comparing against the planned groups for each synthesis (item #5)). | Pages 6-7 |
|  | 13b | Describe any methods required to prepare the data for presentation or synthesis, such as handling of missing summary statistics, or data conversions. | N/A |
|  | 13c | Describe any methods used to tabulate or visually display results of individual studies and syntheses. | Pages 6-7 |
|  | 13d | Describe any methods used to synthesize results and provide a rationale for the choice(s). If meta-analysis was performed, describe the model(s), method(s) to identify the presence and extent of statistical heterogeneity, and software package(s) used. | Pages 6-7 |
|  | 13e | Describe any methods used to explore possible causes of heterogeneity among study results (e.g. subgroup analysis, meta-regression). | N/A |
|  | 13f | Describe any sensitivity analyses conducted to assess robustness of the synthesized results. | N/A |
| Reporting bias assessment | 14 | Describe any methods used to assess risk of bias due to missing results in a synthesis (arising from reporting biases). | Page 7 |
| Certainty assessment | 15 | Describe any methods used to assess certainty (or confidence) in the body of evidence for an outcome. | Page 7 |
| **RESULTS** | | |  |
| Study selection | 16a | Describe the results of the search and selection process, from the number of records identified in the search to the number of studies included in the review, ideally using a flow diagram. | Page 8 |
|  | 16b | Cite studies that might appear to meet the inclusion criteria, but which were excluded, and explain why they were excluded. | Page 8  Figure 1 |
| Study characteristics | 17 | Cite each included study and present its characteristics. | Table 1 |
| Risk of bias in studies | 18 | Present assessments of risk of bias for each included study. | Figures 3A-C |
| Results of individual studies | 19 | For all outcomes, present, for each study: (a) summary statistics for each group (where appropriate) and (b) an effect estimate and its precision (e.g. confidence/credible interval), ideally using structured tables or plots. | Pages 8-17  Tables 2-10 |
| Results of syntheses | 20a | For each synthesis, briefly summarise the characteristics and risk of bias among contributing studies. | Pages 8-17 |
|  | 20b | Present results of all statistical syntheses conducted. If meta-analysis was done, present for each the summary estimate and its precision (e.g. confidence/credible interval) and measures of statistical heterogeneity. If comparing groups, describe the direction of the effect. | Pages 8-17  Tables 2-10 |
|  | 20c | Present results of all investigations of possible causes of heterogeneity among study results. | N/A |
|  | 20d | Present results of all sensitivity analyses conducted to assess the robustness of the synthesized results. | N/A |
| Reporting biases | 21 | Present assessments of risk of bias due to missing results (arising from reporting biases) for each synthesis assessed. | Figure 3A |
| Certainty of evidence | 22 | Present assessments of certainty (or confidence) in the body of evidence for each outcome assessed. | Figures 3A-C |
| **DISCUSSION** | | |  |
| Discussion | 23a | Provide a general interpretation of the results in the context of other evidence. | Pages 17-20 |
|  | 23b | Discuss any limitations of the evidence included in the review. | Pages 20-21 |
|  | 23c | Discuss any limitations of the review processes used. | Pages 20-21 |
|  | 23d | Discuss implications of the results for practice, policy, and future research. | Pages 20-21 |
| **OTHER INFORMATION** | | |  |
| Registration and protocol | 24a | Provide registration information for the review, including register name and registration number, or state that the review was not registered. | Page 22 |
|  | 24b | Indicate where the review protocol can be accessed, or state that a protocol was not prepared. | Page 22 |
|  | 24c | Describe and explain any amendments to information provided at registration or in the protocol. | N/A |
| Support | 25 | Describe sources of financial or non-financial support for the review, and the role of the funders or sponsors in the review. | Page 22 |
| Competing interests | 26 | Declare any competing interests of review authors. | Page 22 |
| Availability of data, code and other materials | 27 | Report which of the following are publicly available and where they can be found: template data collection forms; data extracted from included studies; data used for all analyses; analytic code; any other materials used in the review. | Page 22 |

*From:*  Page MJ, McKenzie JE, Bossuyt PM, Boutron I, Hoffmann TC, Mulrow CD, et al. The PRISMA 2020 statement: an updated guideline for reporting systematic reviews. BMJ 2021;372:n71. doi: 10.1136/bmj.n71

**Supplementary Table 1: Search Strategy and articles retrieved from all databases searched (n=11,794)**

| **#** | **Search Terms** | **PubMed** |
| --- | --- | --- |
| 1 | “elderly” OR “elders” OR “old adults” OR “>65 years old” OR “old age” OR "ageing population" OR "aging population" OR "aged population" OR "ageing cohort" OR "aging cohort" OR "aged cohort" OR "aging group" OR "ageing group" OR "aged group" OR "aging people" OR "ageing people" OR "aged people" OR "aging patients" OR "ageing patients" OR "aged patients" OR "aged participants" OR "aging participants" OR "ageing participants" OR "ageing community" OR "aging community" OR "aged community" OR “retiree” OR “retired” OR “seniors” OR “pensioners” OR “old people” OR “older people” OR “over 65 years” OR “senior citizens” OR “elderly population” OR “elderly people” OR “geriatric” OR “elderly patients” OR “elderly participants” | 543,472*  **(All Fields filter)** |
| 2 | “obesity” OR “obese” OR “overweight” OR “weight gain” OR “body mass index” OR “BMI” OR “excess weight” OR “adiposity” OR “waist circumference” OR “body fat” OR “body fat percentage” OR “visceral fat” OR “body composition” OR “hypertension” OR “high blood pressure” OR “raised blood pressure” OR “raised BP” OR “anti-hypertensive” OR “systolic blood pressure” OR “diastolic blood pressure” OR “diabetes” OR “diabetes mellitus” OR “type 2 diabetes” OR “T2D” OR “T2DM” OR “T2 diabetes” OR “adult-onset diabetes” OR “insulin resistance” OR “non-insulin dependent diabetes” OR “high blood glucose” OR “hyperglycaemia” OR “hyperglycemia” OR “dysglycaemia” OR “dysglycemia” OR “cholesterol” OR “hyperlipidaemia” OR “hyperlipidemia” OR “dyslipidaemia” OR “dyslipidemia” OR “hypercholesterolemia” OR “LDL” OR “HDL” OR “metabolic syndrome” OR “mental health” OR “mental well-being” OR “depression” OR “distressed” OR “depressed” OR “frailty” OR “frail” OR “falls” OR “balance disorders” OR “gait disorders” OR “imbalance” OR “cardiovascular diseases” OR “CVD” OR “heart disease” OR “heart disorders” OR “cardiovascular disorders” OR "ischemic" OR "ischaemic" OR "stroke" OR "cerebrovascular accident" OR "coronary artery disease" OR "myocardial infarction" OR “cardiovascular conditions” OR “heart conditions” OR “cardiovascular events” OR “cardiovascular risk” OR “cardiovascular risk score” OR “cancer” OR “carcinoma” OR “mortality” OR “mortalities” OR “death” OR “loss of life” OR “deceased” OR “survival rate” OR “fatality” OR “fatalities” OR “fatal” | 8,878,596*  **(All Fields filter)** |
| 3 | “cardiorespiratory fitness” OR “CRF” OR “aerobic fitness” OR “aerobic exercise” OR “aerobic capacity” OR “physical activity” OR “aerobic physical activity” OR “moderate-to-vigorous physical activity” OR “MVPA” OR “light intensity physical activity” OR “LIPA” OR “moderate physical activity” OR “vigorous physical activity” OR “aerobic capacity” OR “maximum oxygen uptake” OR “cardiorespiratory endurance” OR “cardiopulmonary fitness” OR “oxygen consumption” OR “VO2 max” OR “endurance capacity” OR “cardiopulmonary exercise testing” OR “CPET” OR “cardiovascular fitness” OR “moderate exercise” OR “vigorous exercise” | 191,310*  **(All Fields filter)** |
| 4 | #1 AND #2 AND #3 | **2,508** |

| **#** | **Search Terms** | **Cochrane** |
| --- | --- | --- |
| 1 | “elderly” OR “elders” OR “old adults” OR “>65 years old” OR “old age” OR "ageing population" OR "aging population" OR "aged population" OR "ageing cohort" OR "aging cohort" OR "aged cohort" OR "aging group" OR "ageing group" OR "aged group" OR "aging people" OR "ageing people" OR "aged people" OR "aging patients" OR "ageing patients" OR "aged patients" OR "aged participants" OR "aging participants" OR "ageing participants" OR "ageing community" OR "aging community" OR "aged community" OR “retiree” OR “retired” OR “seniors” OR “pensioners” OR “old people” OR “older people” OR “over 65 years” OR “senior citizens” OR “elderly population” OR “elderly people” OR “geriatric” OR “elderly patients” OR “elderly participants”  (Word variations have been searched) | **All Text filter** |
| 2 | “obesity” OR “obese” OR “overweight” OR “weight gain” OR “body mass index” OR “BMI” OR “excess weight” OR “adiposity” OR “waist circumference” OR “body fat” OR “body fat percentage” OR “visceral fat” OR “body composition” OR “hypertension” OR “high blood pressure” OR “raised blood pressure” OR “raised BP” OR “anti-hypertensive” OR “systolic blood pressure” OR “diastolic blood pressure” OR “diabetes” OR “diabetes mellitus” OR “type 2 diabetes” OR “T2D” OR “T2DM” OR “T2 diabetes” OR “adult-onset diabetes” OR “insulin resistance” OR “non-insulin dependent diabetes” OR “high blood glucose” OR “hyperglycaemia” OR “hyperglycemia” OR “dysglycaemia” OR “dysglycemia” OR “cholesterol” OR “hyperlipidaemia” OR “hyperlipidemia” OR “dyslipidaemia” OR “dyslipidemia” OR “hypercholesterolemia” OR “LDL” OR “HDL” OR “metabolic syndrome” OR “mental health” OR “mental well-being” OR “depression” OR “distressed” OR “depressed” OR “frailty” OR “frail” OR “falls” OR “balance disorders” OR “gait disorders” OR “imbalance” OR “cardiovascular diseases” OR “CVD” OR “heart disease” OR “heart disorders” OR “cardiovascular disorders” OR "ischemic" OR "ischaemic" OR "stroke" OR "cerebrovascular accident" OR "coronary artery disease" OR "myocardial infarction" OR “cardiovascular conditions” OR “heart conditions” OR “cardiovascular events” OR “cardiovascular risk” OR “cardiovascular risk score” OR “cancer” OR “carcinoma” OR “mortality” OR “mortalities” OR “death” OR “loss of life” OR “deceased” OR “survival rate” OR “fatality” OR “fatalities” OR “fatal”  (Word variations have been searched) |  |
| 3 | “cardiorespiratory fitness” OR “CRF” OR “aerobic fitness” OR “aerobic exercise” OR “aerobic capacity” OR “aerobic physical activity” OR “moderate-to-vigorous physical activity” OR “MVPA” OR “light intensity physical activity” OR “LIPA” OR “moderate physical activity” OR “vigorous physical activity” OR “aerobic capacity” OR “maximum oxygen uptake” OR “cardiorespiratory endurance” OR “cardiopulmonary fitness” OR “oxygen consumption” OR “VO2 max” OR “endurance capacity” OR “cardiopulmonary exercise testing” OR “CPET” OR “cardiovascular fitness” OR “moderate exercise” OR “vigorous exercise”  (Word variations have been searched) |  |
| 4 | #1 AND #2 AND #3 | **2,098** |

| **#** | **Search Terms** | **Scopus** |
| --- | --- | --- |
| 1 | “elderly” OR “elders” OR “old adults” OR “>65 years old” OR “old age” OR "ageing population" OR "aging population" OR "aged population" OR "ageing cohort" OR "aging cohort" OR "aged cohort" OR "aging group" OR "ageing group" OR "aged group" OR "aging people" OR "ageing people" OR "aged people" OR "aging patients" OR "ageing patients" OR "aged patients" OR "aged participants" OR "aging participants" OR "ageing participants" OR "ageing community" OR "aging community" OR "aged community" OR “retiree” OR “retired” OR “seniors” OR “pensioners” OR “old people” OR “older people” OR “over 65 years” OR “senior citizens” **AND** “obesity” OR “obese” OR “overweight” OR “weight gain” OR “body mass index” OR “BMI” OR “excess weight” OR “adiposity” OR “waist circumference” OR “body fat” OR “body fat percentage” OR “visceral fat” OR “body composition” OR “hypertension” OR “high blood pressure” OR “raised blood pressure” OR “raised BP” OR “anti-hypertensive” OR “systolic blood pressure” OR “diastolic blood pressure” OR “diabetes” OR “diabetes mellitus” OR “type 2 diabetes” OR “T2D” OR “T2DM” OR “T2 diabetes” OR “adult-onset diabetes” OR “insulin resistance” OR “non-insulin dependent diabetes” OR “high blood glucose” OR “hyperglycaemia” OR “hyperglycemia” OR “dysglycaemia” OR “dysglycemia” OR “cholesterol” OR “hyperlipidaemia” OR “hyperlipidemia” OR “dyslipidaemia” OR “dyslipidemia” OR “hypercholesterolemia” OR “LDL” OR “HDL” OR “metabolic syndrome” OR “mental health” OR “mental well-being” OR “depression” OR “distressed” OR “depressed” OR “frailty” OR “frail” OR “falls” OR “balance disorders” OR “gait disorders” OR “imbalance” OR “cardiovascular diseases” OR “CVD” OR “heart disease” OR “heart disorders” OR “cardiovascular disorders” OR "ischemic" OR "ischaemic" OR "stroke" OR "cerebrovascular accident" OR "coronary artery disease" OR "myocardial infarction" OR “cardiovascular conditions” OR “heart conditions” OR “cardiovascular events” OR “cardiovascular risk” OR “cardiovascular risk score” OR “cancer” OR “carcinoma” OR “mortality” OR “mortalities” OR “death” OR “loss of life” OR “deceased” OR “survival rate” OR “fatality” OR “fatalities” OR “fatal” **AND** “cardiorespiratory fitness” OR “CRF” OR “aerobic fitness” OR “aerobic exercise” OR “aerobic capacity” OR “aerobic physical activity” OR “moderate-to-vigorous physical activity” OR “MVPA” OR “light intensity physical activity” OR “LIPA” OR “moderate physical activity” OR “vigorous physical activity” OR “aerobic capacity” OR “maximum oxygen uptake” OR “cardiorespiratory endurance” OR “cardiopulmonary fitness” OR “oxygen consumption” OR “VO2 max” OR “endurance capacity” OR “cardiopulmonary exercise testing” OR “CPET” OR “cardiovascular fitness” OR “moderate exercise” OR “vigorous exercise” | 5,534*  **(Title/Abstract filter)** |

| **#** | **Search Terms** | **Web of Science** |
| --- | --- | --- |
| 1 | “elderly” OR “elders” OR “old adults” OR “>65 years old” OR “old age” OR "ageing population" OR "aging population" OR "aged population" OR "ageing cohort" OR "aging cohort" OR "aged cohort" OR "aging group" OR "ageing group" OR "aged group" OR "aging people" OR "ageing people" OR "aged people" OR "aging patients" OR "ageing patients" OR "aged patients" OR "aged participants" OR "aging participants" OR "ageing participants" OR "ageing community" OR "aging community" OR "aged community" OR “retiree” OR “retired” OR “seniors” OR “pensioners” OR “old people” OR “older people” OR “over 65 years” OR “senior citizens” OR “elderly population” OR “elderly people” OR “geriatric” OR “elderly patients” OR “elderly participants” | 571,666*  **(All Fields filter)** |
| 2 | “obesity” OR “obese” OR “overweight” OR “weight gain” OR “body mass index” OR “BMI” OR “excess weight” OR “adiposity” OR “waist circumference” OR “body fat” OR “body fat percentage” OR “visceral fat” OR “body composition” OR “hypertension” OR “high blood pressure” OR “raised blood pressure” OR “raised BP” OR “anti-hypertensive” OR “systolic blood pressure” OR “diastolic blood pressure” OR “diabetes” OR “diabetes mellitus” OR “type 2 diabetes” OR “T2D” OR “T2DM” OR “T2 diabetes” OR “adult-onset diabetes” OR “insulin resistance” OR “non-insulin dependent diabetes” OR “high blood glucose” OR “hyperglycaemia” OR “hyperglycemia” OR “dysglycaemia” OR “dysglycemia” OR “cholesterol” OR “hyperlipidaemia” OR “hyperlipidemia” OR “dyslipidaemia” OR “dyslipidemia” OR “hypercholesterolemia” OR “LDL” OR “HDL” OR “metabolic syndrome” OR “mental health” OR “mental well-being” OR “depression” OR “distressed” OR “depressed” OR “frailty” OR “frail” OR “falls” OR “balance disorders” OR “gait disorders” OR “imbalance” OR “cardiovascular diseases” OR “CVD” OR “heart disease” OR “heart disorders” OR “cardiovascular disorders” OR "ischemic" OR "ischaemic" OR "stroke" OR "cerebrovascular accident" OR "coronary artery disease" OR "myocardial infarction" OR “cardiovascular conditions” OR “heart conditions” OR “cardiovascular events” OR “cardiovascular risk” OR “cardiovascular risk score” OR “cancer” OR “carcinoma” OR “mortality” OR “mortalities” OR “death” OR “loss of life” OR “deceased” OR “survival rate” OR “fatality” OR “fatalities” OR “fatal” | 3,336,149*  **(Keyword Plus filter)** |
| 3 | “cardiorespiratory fitness” OR “CRF” OR “aerobic fitness” OR “aerobic exercise” OR “aerobic capacity” OR “physical activity” OR “aerobic physical activity” OR “moderate-to-vigorous physical activity” OR “MVPA” OR “light intensity physical activity” OR “LIPA” OR “moderate physical activity” OR “vigorous physical activity” OR “aerobic capacity” OR “maximum oxygen uptake” OR “cardiorespiratory endurance” OR “cardiopulmonary fitness” OR “oxygen consumption” OR “VO2 max” OR “endurance capacity” OR “cardiopulmonary exercise testing” OR “CPET” OR “cardiovascular fitness” OR “moderate exercise” OR “vigorous exercise” | 150,600*  **(All Fields filter)** |
| 4 | #1 AND #2 AND #3 | **1,649** |

**Supplementary Table 2: Data extraction tool**

| **Interventions** | | | | | | | | | | | |
| --- | --- | --- | --- | --- | --- | --- | --- | --- | --- | --- | --- |
| **Author** | **Year** | **Country** | **Age** | **Follow-up (months)** | **Sample size (N)** | **N women** | **N men** | **Description of intervention OR exposure measure** | **Outcome measured** | **Main findings** | **Notes** |
|  |  |  |  |  |  |  |  |  |  |  |  |
|  |  |  |  |  |  |  |  |  |  |  |  |
|  |  |  |  |  |  |  |  |  |  |  |  |
| **Observational studies: Physical activity** | | | | | | | | | | | |
| **Author** | **Year** | **Country** | **Age** | **Follow-up (months)** | **Sample size (N)** | **N women** | **N men** | **Description of intervention OR exposure measure** | **Outcome measured** | **Main findings** | **Notes** |
|  |  |  |  |  |  |  |  |  |  |  |  |
|  |  |  |  |  |  |  |  |  |  |  |  |
|  |  |  |  |  |  |  |  |  |  |  |  |
| **Observational studies: Cardiorespiratory fitness (CRF)** | | | | | | | | | | | |
| **Author** | **Year** | **Country** | **Age** | **Follow-up (months)** | **Sample size (N)** | **N women** | **N men** | **Description of intervention OR exposure measure** | **Outcome measured** | **Main findings** | **Notes** |
|  |  |  |  |  |  |  |  |  |  |  |  |
|  |  |  |  |  |  |  |  |  |  |  |  |
|  |  |  |  |  |  |  |  |  |  |  |  |

**Supplementary Table 3: Synthesis of results by within-study comparison of age groups ≥65 years**

| **Articles that stratify association results by** | **Outcome** | **Age** | **Difference by age group** |
| --- | --- | --- | --- |
| LaMonte et al., 2018 | Mortality | 70-79 vs 80-89 vs ≥90 | NONE  “No difference in results for the subgroup aged ≥90 vs 80-89-year age group.” |
| Kokkinos et al., 2022 | Mortality | ≥70; 70-79 vs 80-95 | SOME  Statistically significant in both groups, but lower effect size in older group. |
| Phan et al., 2022 | Mortality | ≥70; 70-79 vs 80-90 | NONE  Low fitness is associated with greater mortality risk across all age groups. In addition, patients aged 80-90 with low fitness had the highest risk of all-cause mortality (HR 10.6, 95% CI 7.8-14.4, p<0.001); those with the highest fitness had comparable or even lower HR risk (HR 2.9, 95% CI 1.2-7.2, p=0.02) compared with their younger counterparts aged 70-80 years with moderate or low fitness, and better survival compared with patients aged 60-70 years with low fitness. |
| Barbiellini Amidei et al., 2022 | CVD | ≥65; PA at 70 vs 75 vs 80 vs 85 | YES  This risk reduction in men was significant for PA measured at 70-75 years, but not at ≥80 years. |
| Emerson & Gay, 2017 | CVD | ≥65; 65-74 vs ≥75 | NONE  No significant difference in the effect of MVPA on CVD risk by age subgroup (65-74 vs ≥75). |
| Soares-Miranda et al., 2016 | CVD | ≥65; 65-74 vs ≥75 | SOME  Results were generally similar when stratified by sex and age group (65-74 vs ≥75), although in the ≥75 group only moderate and high (not low) exercise intensity significantly reduced risk of stroke compared to no exercise.  In the ≥75 group, only the highest walking distance (≥49 blocks/week) was associated with lower coronary heart disease compared to 0-5 blocks/week. |
| Lai et al., 2022 | BMI | 65-98; 65-74 vs ≥75 | YES  These associations were only significant in those aged 65-74, not ≥75 years. |
| Gallardo-Alfaro et al., 2019 | MetS | ≥65; 65-69 vs ≥70 | NONE  Total activity was lower in MetS participants compared to non-MetS participants in those aged: 65-69 (median 59.6 [IQR 73.3] vs 81.2 [IQR 43.1], p=0.037) and ≥70 (median 51.3 [IQR 50.8] vs 82.8 [IQR 42.5], p<0.001). |
| Marques et al., 2020 | Depression | ≥65; 65-79 vs ≥80 | NONE  For moderate and vigorous PA for both men and women aged 65-79 or ≥80 years, being active at least 1 time/week in the present was consistently associated with significantly lower odds of having depressive symptoms. |
| McDowell et al., 2018 | Depression | ≥70; 70-79 vs ≥80 | YES  Those aged 70-79 years (n=896) meeting PA guidelines had significantly lower odds of depression (OR 0.35, 95% CI 0.18-0.68, p<0.001, p<0.01); this was not found to be significant in those aged >80 years (n=255). |
| Netz et al., 2021 | Frailty and falls | ≥65;  Women: 65-74 vs ≥75  (men were all older) | NONE for PA but YES for CRF  Among women 65-75 years old, predicted VO2 was associated with frailty. No significant association with men or women >75 years old. |
| Lin et al., 2022 | Frailty and falls |  | NONE |
| Rogers et al., 2017 | Frailty and falls | ≥65; 65-69 vs 70-74 vs 75-79 vs ≥80 | NONE  Moderate PA at least once a week was associated with improved frailty progression of the cohorts aged:  65-69: (B = -0.034, 95% CI -0.047, -0.021, p<0.0001);  70-74: (B = -0.028, 95% CI -0.044, -0.012, p=0.001);  75-79: (B = -0.024, 95% CI -0.042, -0.007, p=0.005); and  80+: (B = -0.039, 95% CI -0.054, -0.023, p<0.0001). |
